# Multi-omics analysis reveals the core microbiome and biomarker for nutrition degradation in alfalfa silage fermentation

**DOI:** 10.1128/msystems.00682-24

**Published:** 2024-10-23

**Authors:** Yuan Wang, Yunlei Sun, KeXin Huang, Yu Gao, Yufan Lin, Baojie Yuan, Xin Wang, Gang Xu, Luiz Gustavo Nussio, Fuyu Yang, Kuikui Ni

**Affiliations:** 1 College of Grassland Science and Technology, China Agricultural University, Beijing, China; 2 Frontier Technology Research Institute, China Agricultural University, Shenzhen, China; 3 Department of Animal Science, University of São Paulo, Piracicaba, Brazil; 4 College of Animal Science, Guizhou University, Guiyang, China; China University of Geosciences, Wuhan, Hubei, China

**Keywords:** silage, metagenomics, culturomics, ammonia-N, butyric acid

## Abstract

Alfalfa (*Medicago sativa* L.) is one of the most extensively cultivated forage crops globally, and its nutritional quality critically influences the productivity of dairy cows. Silage fermentation is recognized as a crucial technique for the preservation of fresh forage, ensuring the retention of its vital nutrients. However, the detailed microbial components and their functions in silage fermentation are not fully understood. This study integrated large-scale microbial culturing with high-throughput sequencing to thoroughly examine the microbial community structure in alfalfa silage and explored the potential pathways of nutritional degradation via metagenomic analysis. The findings revealed an enriched microbial diversity in silage, indicated by the identification of amplicon sequence variants. Significantly, the large-scale culturing approach recovered a considerable number of unique microbes undetectable by high-throughput sequencing. Predominant genera, such as *Lactiplantibacillus*, *Leuconostoc*, *Lentilactobacillus*, *Weissella*, and *Liquorilactobacillus*, were identified based on their abundance and prevalence. Additionally, genes associated with Enterobacteriaceae were discovered, which might be involved in pathways leading to the production of ammonia-N and butyric acid. Overall, this study offers a comprehensive insight into the microbial ecology of silage fermentation and provides valuable information for leveraging microbial consortia to enhance fermentation quality.

**IMPORTANCE:**

Silage fermentation is a microbial-driven anaerobic process that efficiently converts various substrates into nutrients readily absorbable and metabolizable by ruminant animals. This study, integrating culturomics and metagenomics, has successfully identified core microorganisms involved in silage fermentation, including those at low abundance. This discovery is crucial for the targeted cultivation of specific microorganisms to optimize fermentation processes. Furthermore, our research has uncovered signature microorganisms that play pivotal roles in nutrient metabolism, significantly advancing our understanding of the intricate relationships between microbial communities and nutrient degradation during silage fermentation.

## INTRODUCTION

The annual global production of silage surpasses 1 billion tons, serving as a critical component in the diets of approximately 140 million dairy cows and other ruminant animals worldwide, according to the Food and Agriculture Organization of the United Nations (1–7). Among various forages, alfalfa is notably esteemed as the "king of forage" due to its superior protein content and high rumen digestibility (8–10). Ensiling, a prevalent method for preserving fresh forages like whole-plant corn, alfalfa, and forage oats, employs lactic acid fermentation under anaerobic conditions to not only preserve the nutrients in raw materials but also enhance their quality and palatability (11, 12). Extensive research has underscored the pivotal role of microbes in maintaining the quality of silage fermentation (9, 13–15). Consequently, a thorough analysis of the diversity, composition, and functionality of silage microbes is vital to understanding their impact on fermentation quality during the ensiling process (16). Nevertheless, previous research often focused on isolated batches of experiments conducted at specific sites or a limited number of samples under controlled laboratory conditions (17). Additionally, the strong specialization of microbial niches and the limited adaptability of traditional culture methods have led to the prevalence of numerous unclassified and uncultured entities within the "microbial dark matter" (18–20), impeding the advancement of microbial ecology studies in silage fermentation environments.

High-throughput sequencing technology has substantially enhanced our understanding of the composition and diversity of silage microbiota (2, 8, 11, 21, 22). Nevertheless, functional characterization and verification at biological and molecular levels still rely predominantly on culture-based experiments (23–26). The cultivation of microorganisms that harbor unknown genes or produce specific metabolites remains crucial (25, 27). Culturomics, a pioneering approach in microbiome research, integrates various isolation and culture conditions with 16S rRNA gene sequencing or other advanced technologies (24, 28, 29), significantly enriching our knowledge of the diversity of culturable microorganisms. However, culture-based studies often focus on microorganisms that are easily culturable under laboratory conditions, potentially overlooking important taxa within the community (17, 21, 22, 30, 31). Therefore, a combined approach that leverages both high-throughput sequencing and culturomics offers a comprehensive method to explore the composition and diversity of silage microbiota, providing fresh insights into the so-called microbial dark matter within silage fermentation.

The diverse microorganisms present in silage fermentation play a pivotal role in the degradation of crude protein or amino acids in feed, as well as in carbohydrate metabolism (9, 32, 33). Gaining a deep understanding of the genomic and functional components of the microbial community is essential for effectively managing silage fermentation and preserving the nutrients in raw materials (4, 14, 16, 30). Previous studies on silage microbiota have primarily utilized 16S rRNA gene amplicon methods (17, 22, 31). To control feed fermentation quality through microbial insights, it is imperative to understand the interactions and metabolic pathways involving silage microbiota (9, 16, 31). Thus, the application of metagenomics to explore the functional characteristics of these microorganisms promises to enhance our understanding of the complex interactions within the microbial communities in silage (18, 30, 34, 35). This approach not only expands our knowledge of the microbial diversity but also provides insights into the functional composition and metabolic capabilities of the microorganisms involved in silage fermentation.

**Fig 1:**
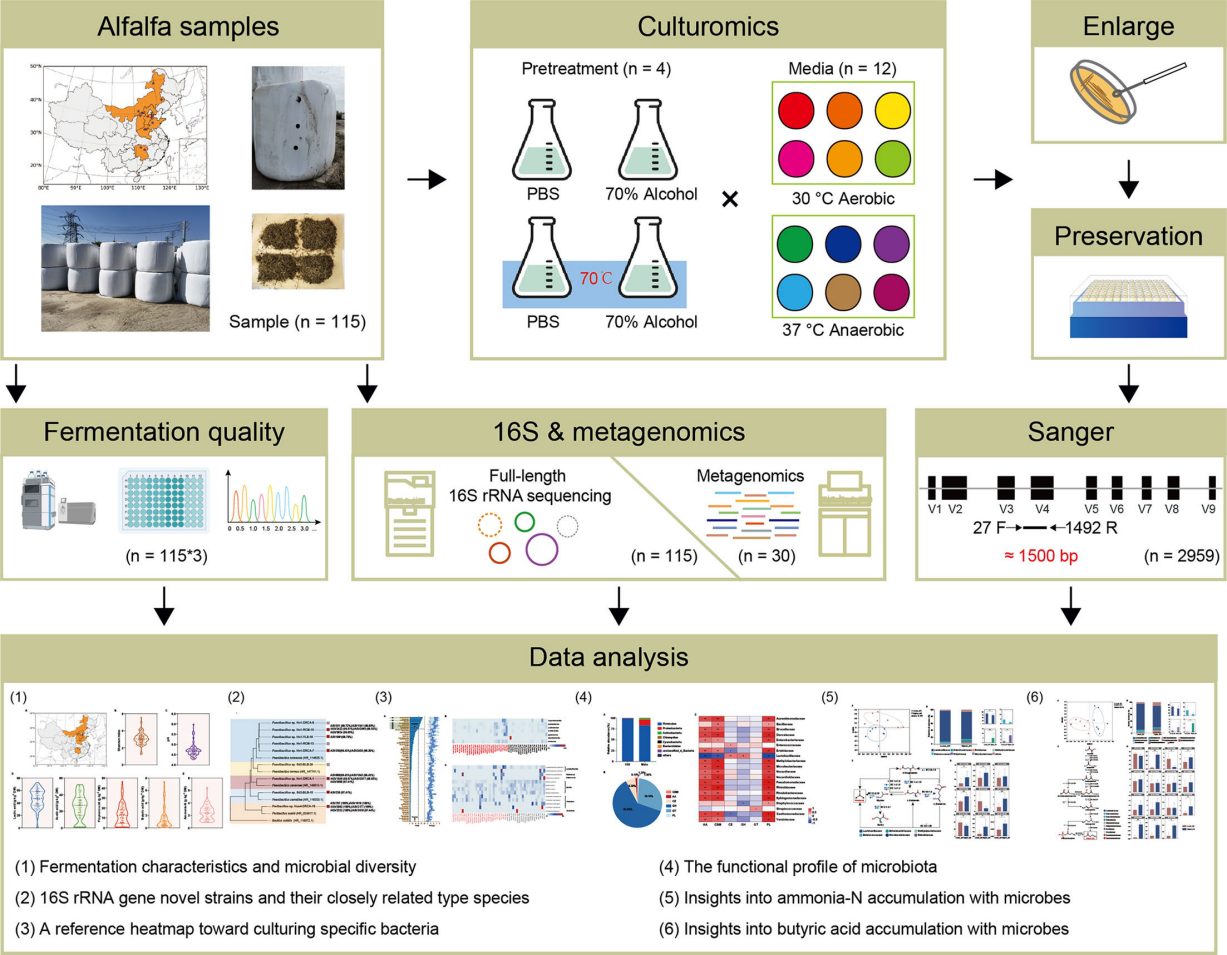
Schematic diagram of the experiment. Geographic distribution of sampling sites across different farms. Large-scale isolation and cultivation of microbes by 48 different microbial culture conditions, including four pretreatment methods and 12 types of medium. The isolates (*n* = 2,959) were sequenced by Sanger sequencing, then all isolates were stocked in 20% (vol/vol) glycerol broth at −80°C. Also, we detected the fermentation quality and chemical components, and PacBio SMRT full-length 16S rRNA gene sequencing was conducted on the original alfalfa samples, and metagenomic sequencing was performed on the different fermentation quality of alfalfa samples.

Current research lacks a systematic and in-depth understanding of the microbial community structure and metabolic functions in alfalfa silage. In this study, we collected 115 samples of alfalfa silage from 13 different farms (Fig. 1) and conducted a comprehensive analysis of the bacterial community composition, diversity, and core microbiota within the silage. The objective of this study was to identify the key microbes involved in silage and elucidate the mechanisms by which they regulate nutrient metabolism. This research aims to lay the foundation for precisely controlling silage fermentation quality using microbial information.

## RESULTS

### An overview of fermentation characteristics and microbial diversity

**Fig 2:**
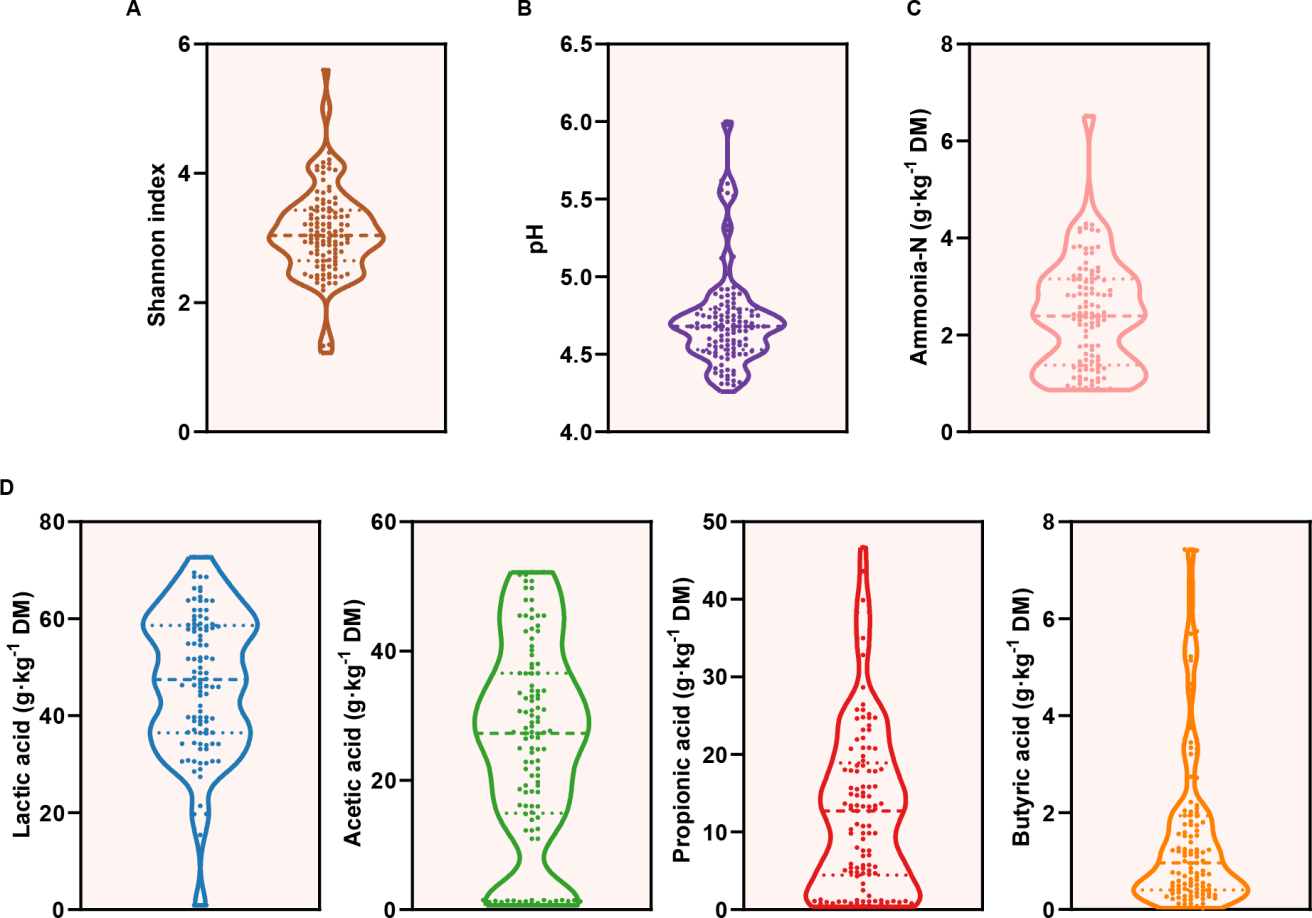
Taxonomic diversity and fermentation index in alfalfa samples. (**A**) Alpha diversity from each sample is based on the Shannon index. (**B**) The pH values of the alfalfa sample. (**C**) Ammonia-N contents. (**D**) The content of organic acids was detected by a high-performance liquid chromatograph.

To investigate the characteristics and functions of the microbiota in silage, a comprehensive collection of 115 samples was obtained from 13 different farms across China. The microbial diversity within these samples was assessed using the Shannon index, revealing significant variations among them (Fig. 2A). The pH of the silage, which is strongly linked to its organic acid content, plays a critical role in the overall quality of the silage (9, 13). Organic acids, such as lactic acid and acetic acid, are beneficial as they contribute to a rapid decline in pH, enhancing the preservation quality. Conversely, butyric acid, produced by less desirable microbial activities, emits a strong odor and can significantly decrease the dry matter intake of the silage by animals (36, 37). Furthermore, ammonia-N, which is produced by microorganisms, like clostridia and enterobacteria, through protein degradation, has been shown to negatively impact animal production efficiency when present at high levels (1, 32, 33, 38, 39). This study observed marked differences in the levels of pH (Fig. 2B), ammonia-N (Fig. 2C), and organic acids (Fig. 2D) across the silage samples. The correlation analysis indicated a significant positive correlation between pH and butyric acid content (Fig. S1). These results not only confirm the representativeness of the samples collected but also provide a solid foundation for further exploration into the structural and functional dynamics of microbial communities under different fermentation quality.

### Construction of cultivable microbial resource library

We conducted a study on microbial diversity by utilizing 48 different microbial culture conditions, which consisted of 12 culture media (Table S1) and four pretreatment methods (Table S2). The results of the isolation process are presented in Fig. S2. Following isolation and purification, microbial colonies on solid culture media were collected for 16S rRNA amplification (>1.4 kb). These isolates were then classified into 231 bacterial taxa using EZBioCloud and NCBI 16S rRNA databases, with a 97% sequence clustering threshold. To further classify the 2,959 bacterial strains (alBM), we utilized the UniProt reference gene database. Consequently, these strains were assigned to 231 species, spanning across three phyla and 55 genera. A detailed classification information and the full-length 16S rRNA gene sequence of each colony can be found in PRJNA983448. Furthermore, we constructed a phylogenetic tree based on the distance between sequence pairs (Fig. S3). This tree provides valuable insights into the evolutionary relationship of alBM microbial species and serves as a foundation for future investigations on their biological characteristics.

### The microbial taxa in silage fermentation

**Fig 3:**
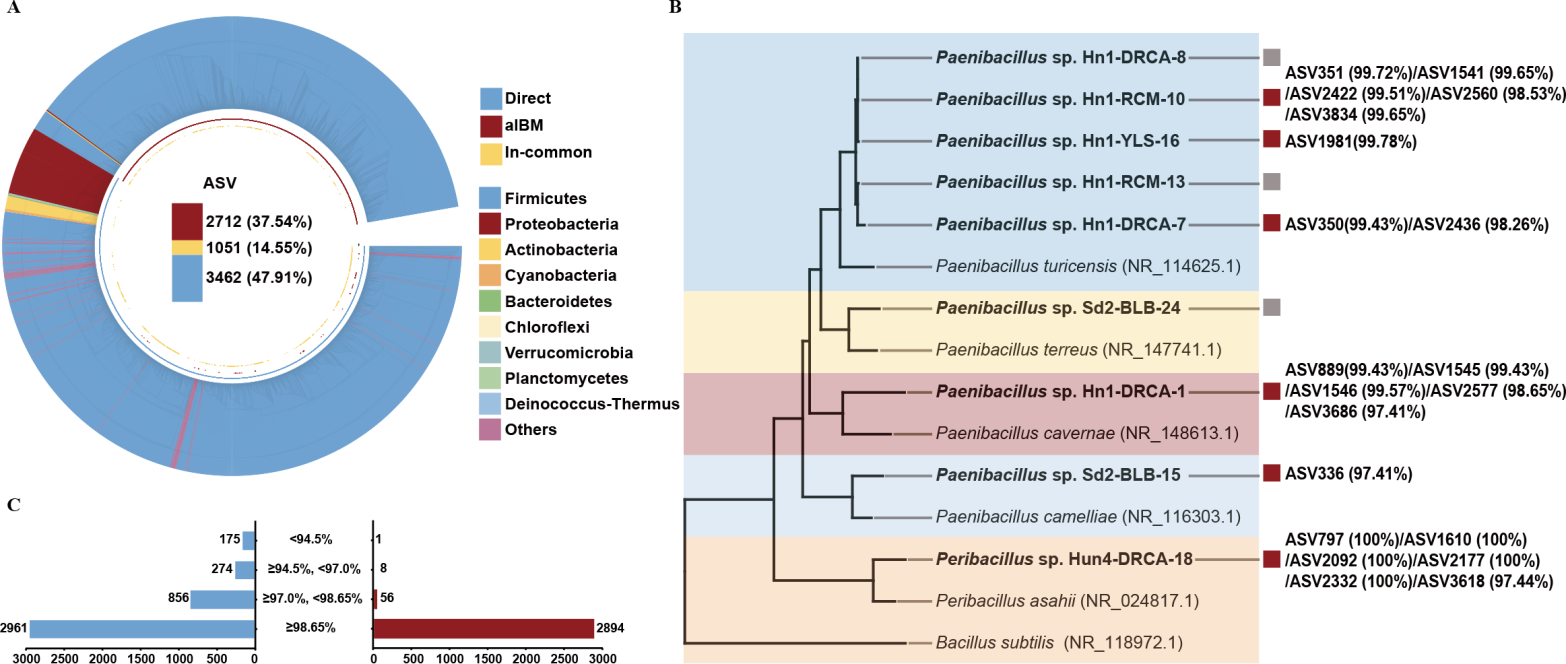
Numerical and novel taxonomic comparisons of ASVs detected by direct sequencing and alBM. (**A**) ASV-based neighbor-phylogenetic tree by direct sequencing and alBM. Direct (blue, 47.91%): the proportion of ASVs by direct sequencing. alBM (red, 37.54%): the proportion of strains by the cultivated. In-common (yellow,14.55%): the proportion of ASVs shared by both collections. (**B**) The ASV number of potentially novel strains detected by direct sequencing (blue) and cultivated (red), based on the classification thresholds at species levels (97.00%). (**C**) A maximum-likelihood tree based on the 16S rRNA gene novel strains and their closely related type species. *Bacillus subtilis* (NR_118972.1) was used as an outgroup. Red solid squares indicate these strains that can match the ASVs exceed 97% of the direct sequencing. Gray solid squares indicate these strains that cannot match ASVs less than 97% generated in this study.

We undertook a detailed analysis of the microbial structure and potential novel taxa in silage by integrating direct sequencing with culturomics. After the exclusion of adapters, barcodes, low-quality reads, and chimeras from the raw data, we obtained 4,580,090 high-quality clean reads, averaging 39,827 clean reads per sample. The amplicon sequence variants (ASVs) were assigned at single-nucleotide resolution using DADA2, resulting in 4,266 distinct ASVs. It is important to note that direct sequencing methods may detect both viable and non-viable members of the microbial community. A combined approach of direct sequencing and culturomics yielded a total of 7,225 ASVs. The potential novelty of these ASVs was assessed through a high-throughput BLAST search against the NCBI 16S rRNA database (Fig. 3A). Among these, 458 ASVs (6.34%) were identified as potential novel taxa, whereas 6,767 ASVs (93.66%) matched known species (Fig. 3B).

Through culturomics, we isolated 2,959 strains, of which 247 were successfully correlated with those identified via direct sequencing, achieving an identification rate exceeding 97% (Table S3). Notably, we discovered that 20 potential novel ASVs had over 97% sequence identity with the 16S rRNA gene sequences of the potential novel strains cultured in this study (Table S4), potentially filling gaps in the "microbial dark matter" unidentifiable by direct sequencing alone.

Furthermore, a classification analysis at both the genus and species levels was conducted for the 7,225 ASVs. Results illustrated in Fig. S4 revealed that 299 genera were detected exclusively by direct sequencing, six genera solely by culturomics, and 49 genera by both methods. At the species level, 920 species were specific to direct sequencing, 92 to culturomics, and 139 were identified by both techniques.

These findings underscore that while direct sequencing is adept at capturing a more comprehensive spectrum of ASVs and a diverse range of taxa, it may fall short in identifying certain taxa from environmental samples. Crucially, we must also remain vigilant about the potential presence of dead or inactive cells, which can be overlooked by high-throughput sequencing methods. This insight underscores the importance of a nuanced approach in interpreting sequencing data, taking into account the complexity of the samples and the limitations of sequencing technologies to ensure the accuracy and reliability of our research outcomes. Therefore, the combined use of direct sequencing and culturomics offers a robust, complementary approach to advancing our understanding of bacterial diversity and addressing the cultivation challenges associated with environmental samples.

### The identification of core- and pan-microbiota

**Fig 4:**
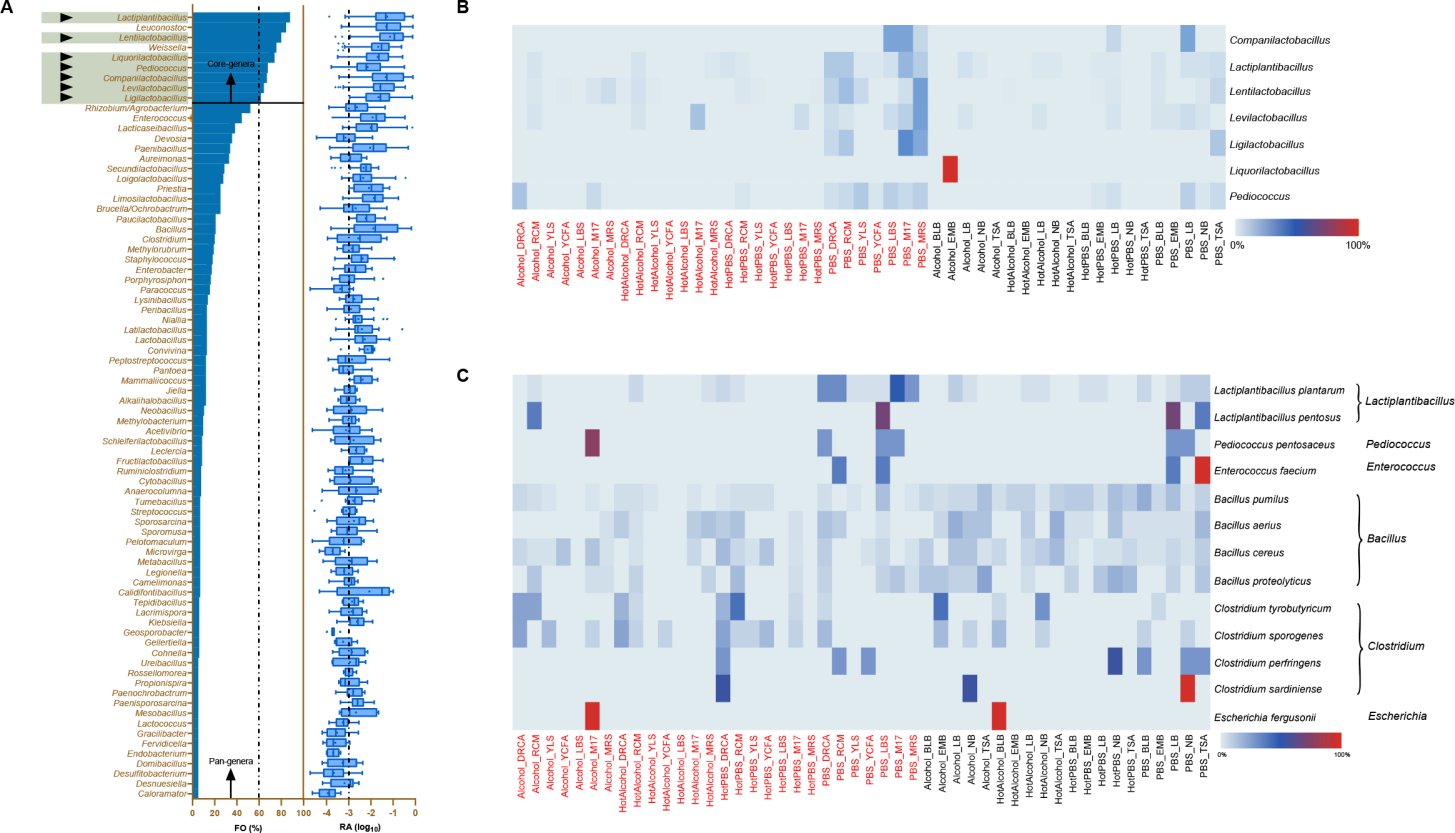
The core- and pan-microbiota and associated cultures. (**A**) The alBM coverage of the core- and pan-genera of the bacteria. The bar chart shows the FO (frequency of occurrence) of each genus in the analyzed samples (definition: FO = 100% is defined when a genus is present in all samples, while FO = 0 is defined when a genus is present in none of all samples); the box-and-whiskers plot shows the RA (relative abundance) of each taxon, center line: median, bounds of box: quartile, whiskers: Tukey extreme. The RA is exhibited in the percentage value logarithm. Core-genera: genera with FO >60% and an average RA >0.1% (log_10_ RA > −3); pan-genera: genera with FO >5%. The cutoff values for core- and pan-genera are marked with vertical dashed lines in the panel; black triangle markers: core-genera for alBM. (**B**) Heatmap of core-genera bacteria of silage microbiota under each of the 48 culture methods (red, aerobic condition; blue, anaerobic condition). (**C**) Heatmap of enriched specific bacteria of microbiota under each of the 48 culture methods.

To determine if alBM accurately represents the microbial community in alfalfa silage, we investigated its coverage of the potential core- and pan-microbiota involved in fermentation processes. The microbial composition of the collected samples was analyzed using 16S rRNA gene amplicon sequencing technology for this purpose. The core-genera were defined as microbial with a frequency of occurrence (FO) >60% and a relative abundance (RA) >0.1%, while the pan-genera were defined as microbial species with an FO >5% (40). Analysis of the amplicon data from the silage samples identified a total of nine core-genera and 57 pan-genera (Fig. 4A). The proportion of core-genera was 80.45%, while the proportion of pan-genera was 98.33%. Figure 4A illustrates that alBM consists of seven core-genera and 28 pan-genera, with reading proportions of 66.12% and 79.22%, respectively. These findings indicate that alBM effectively captures the diversity of the microbial community in alfalfa silage, serving as a foundation for further investigation into its microbial functional characteristics.

In this study, we identified a total of nine core genera based on FO >60% and RA >0.1% criteria and successfully cultivated seven core genera under our cultivation conditions. The optimal cultivation conditions for each genus are shown in Fig. 4B. Among these genera, the core bacterial genus *Liquorilactobacillus* could be enriched exclusively on the EMB medium after pretreatment with alcohol, whereas *Ligilactobacillus* could grow solely under anaerobic cultivation conditions. The genus *Bacillus* constitutes a significant 25.08% of the cultivable microorganisms. In contrast, its relative abundance as detected through direct sequencing techniques was notably lower, at 3.34%. This genus is well known for its strong adaptability to diverse environmental conditions, enabling it to thrive in a wide range of microbial culture conditions (Fig. 4C).

*Clostridium* and *Enterobacter* are commonly implicated in the formation of ammonia-N and butyric acid during ensiling processes that contribute to the degradation of crude protein and a consequent reduction in nutritional value. In our study, we observed the proliferation of *Clostridium* species, such as *Clostridium tyrobutyricum*, *Clostridium sporogenes*, *Clostridium perfringens*, and *Clostridium sardiniense*, in DRCA medium (Fig. 4C). Notably, DRCA medium was not optimal for cultivating *Clostridium*, except for *Clostridium sardiniense*. Instead, RCM medium showed the highest enrichment ratio (31.58%) for *Clostridium butyricum*. On the other hand, *Enterobacter fergusonii*, from the *Enterobacter* genus, could only be enriched on NB or EMB medium following alcohol treatment. As we know, *Enterobacter* is usually detectable and relatively abundant in the early stages of silage fermentation. Regrettably, our study did not yield a significant amount of *Enterobacter*, highlighting the necessity to refine our experimental protocols to enrich better and explore suitable culture conditions for these bacteria.

### The functional profile of microbiota

We conducted metagenomic sequencing using the Illumina platform to explore the correlation between microbial community and fermentation quality (Table S5). The sequencing produced 0.31 Tb raw reads and 0.30 Tb clean reads. Following quality control, 91.66% of the bases had a quality score of 30 or higher, and over 97.06% had a quality score of 20 or higher, indicating the high data accuracy of the clean reads. The samples were assembled using the Megahit software, with an average utilization of 63.07% of the reads for contig assembly. The contigs were subsequently clustered into 3.57 million non-redundant genes (unigenes).

**Fig 5:**
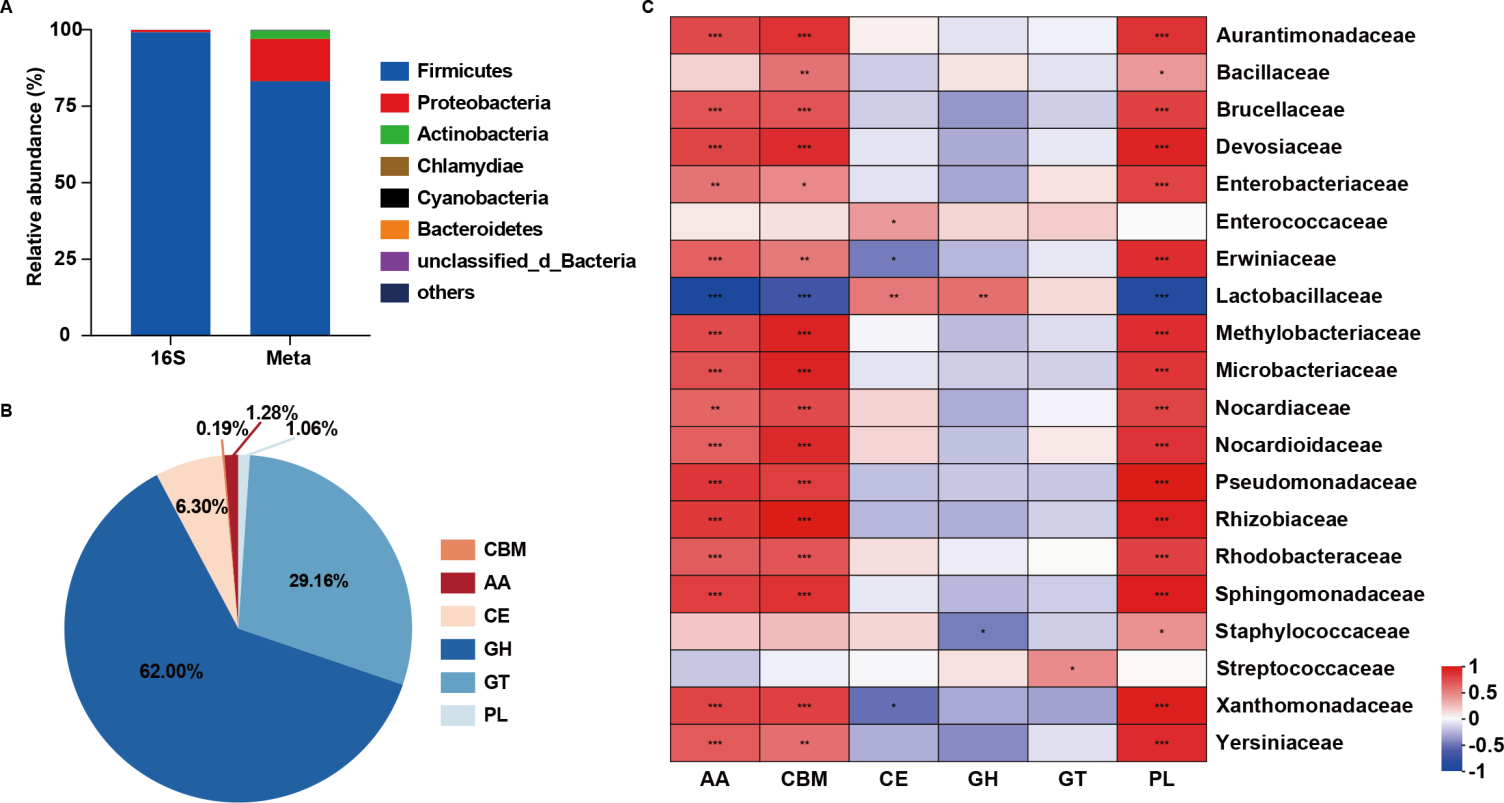
Taxonomic distribution and functional annotation of the microbiota. (**A**) Phylum-level distributions in samples based on 16S amplicon and metagenomic data. (**B**) Functional annotations of the non-redundant gene. Annotation results obtained using CAZy. (**C**) Relationship between the abundance of Cazys and microbiome. GH, glycoside hydrolase; GT, glycosyltransferase; PL, polysaccharide lyase; CE, carbohydrate esterase; AA, auxiliary activity; CBM, carbohydrate-binding modules.

Taxonomic annotations were assigned to 46.96% of the unigenes. Of these, 45.00% were identified as prokaryotes (bacteria and archaea), accounting for 97.75% of the total annotated unigenes. Eukaryotic unigenes, including fungi, protozoa, algae, and plants, accounted for only 0.86% of the total annotated unigenes. Viral genes comprised a mere 0.40% of the annotated unigenes, indicating an extremely low proportion. Additionally, a comparative analysis of amplicon and metagenomic data confirmed a high degree of agreement in the microbial composition of the samples (Fig. 5A). Next, we conducted an analysis of the proteomic content and function of the silage microbiota by searching against the KEGG and CAZy databases. Our findings revealed that most carbohydrate enzymes in the silage microbiome were glycoside hydrolase (GH; 62.00%), followed by glycosyl transferase (GT; 29.16%) (Fig. 5B). We then identified the different relationships between protein contents and the microbial taxa (Fig. 5C), and found positive correlations between CE and the genus belonging to the Lactobacillaceae, as well as between GH and the genus belonging to the Enterococcaceae. Furthermore, we observed significant positive correlations between AA, CBM, and PL with the Enterobacteriaceae, Rhizobacteriaceae, Microbacteriaceae, and Methylobacteriaceae.

### Insights into ammonia-N and butyric acid accumulation with microbes

**Fig 6:**
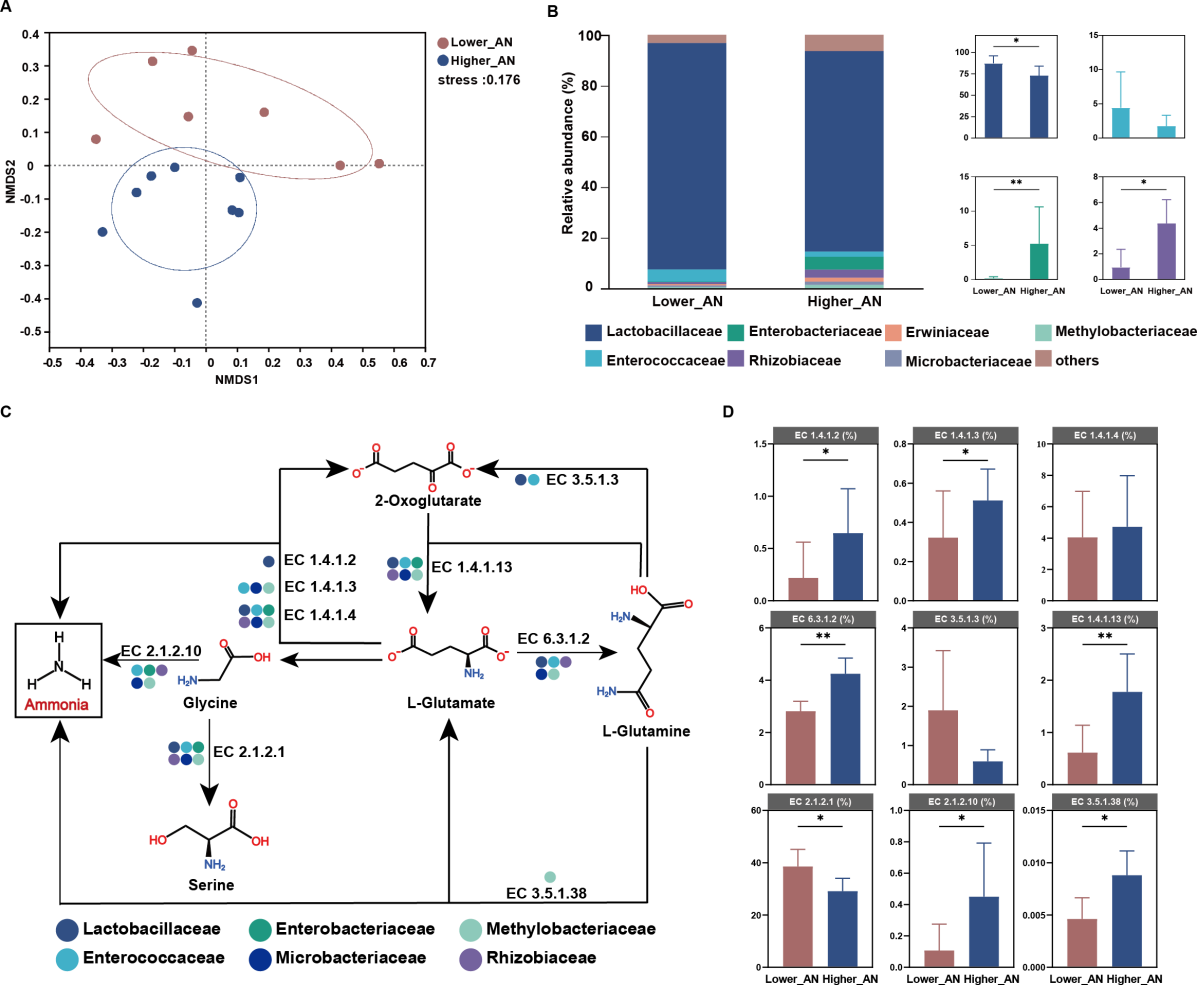
Insights into nitrogen metabolism with microbes. (**A**) Differences in the silage microbiota of the lower_AN and higher_AN were visualized by NMDS. (**B**) Microbiota difference between lower_AN and higher_AN at the family level. (**C**) Bacteria enriched in the fermentation contained key enzymes in each step of the nitrogen metabolism pathway. (**D**) The bar chart shows the differences in key enzymes involved in the nitrogen metabolism pathway between lower_AN and higher_AN. The EC numbers were obtained from the KEGG database. lower_AN, lower ammonia-N content; higher_AN, higher ammonia-N content; NMDS, non-metric multidimensional scaling; *P < 0.05, **P < 0.01, ***P < 0.001.

The content of crude protein is closely related to the levels of ammonia-N. During ensiling, the activities of plant and microbial proteases lead to the breakdown of proteins into peptides and amino acids, which are further deaminated to form ammonia-N (1). High levels of ammonia-N in silage are indicative of protein degradation, which not only reduces the nutritional quality of the silage by decreasing the availability of amino acids for ruminants but also impacts palatability and feed intake (33). The samples were divided into two groups based on their ammonia-N content: the lower ammonia-N content group (lower_AN; *n* = 7, ammonia-N content <1.00 g/kg DM) and the higher ammonia-N content group (higher_AN; *n* = 8, ammonia-N content >4.00 g/kg DM). Non-metric multidimensional scaling (NMDS) analysis demonstrated a clear separation in microbial composition between lower_AN and higher_AN (Fig. 6A). The analysis suggests that ammonia-N content may influence the microbial dynamics within the silage.

It is noteworthy that Lactobacillaceae, typically predominant in well-fermented silage, were significantly more abundant in the lower_AN group, as shown in Fig. 6B and Fig. S5. This suggests their beneficial role in promoting desirable fermentation outcomes. Conversely, the higher_AN group was notably enriched with Enterobacteriaceae. This family might contribute to undesirable fermentation by metabolizing water-soluble carbohydrates, ultimately leading to increased production of fermentation byproducts like ammonia-N. Rhizobacteriaceae was also enriched in the samples, and we propose two potential reasons for this phenomenon. First, the slow or insufficient acidification resulting from sluggish silage fermentation may lead to residual DNA of Rhizobacteriaceae. Furthermore, while metagenomic analysis can identify genes associated with metabolic pathways in Rhizobacteriaceae, their expression may remain inactive. Therefore, employing transcriptomics to ascertain the actual expression of these genes becomes crucial in the future. To delve deeper into the functional capabilities of these microbiomes, we examined the metabolic pathways differentially expressed between the lower_AN and higher_AN groups, as depicted in Fig. 6C and Fig. S6. Notably, key enzymes involved in the ammonia-N formation pathway, including EC 1.4.1.2, EC 1.4.1.3, EC 1.4.1.13, EC 2.1.2.10, EC 3.5.1.38, and EC 6.3.1.2, were significantly overrepresented in the higher_AN group (Fig. 6D). This enrichment suggests a propensity for these bacteria to convert available nitrogen into ammonia-N, potentially decreasing the feed’s nitrogen utilization efficiency, as evidenced in Fig. S7. However, further research is required to confirm the expression of these enzyme genes within these microbial communities and to fully understand their impact on silage fermentation quality.

**Fig 7:**
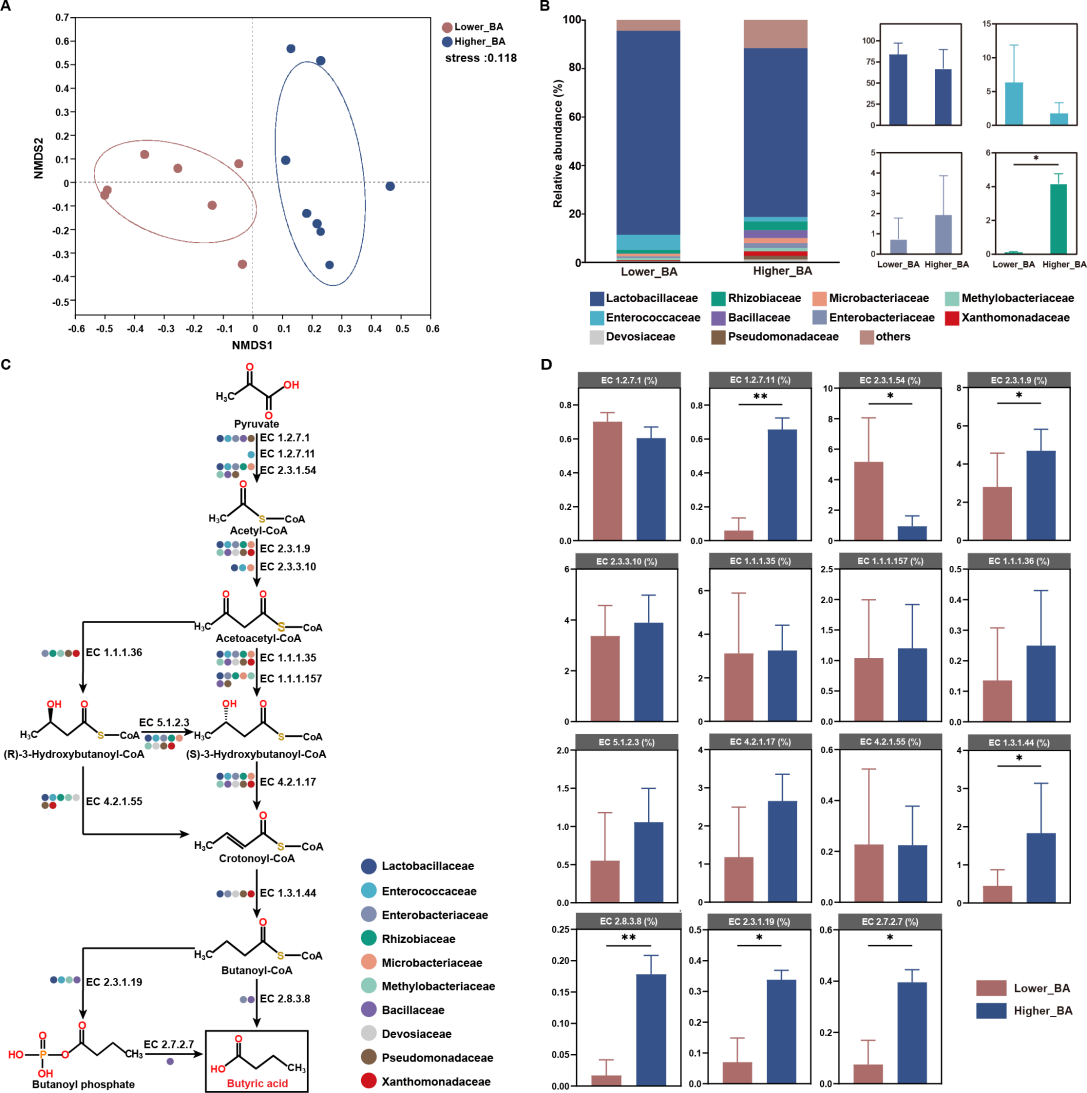
Insights into butyric acid accumulation with microbes. (**A**) Differences in the microbiota of lower_BA and higher_BA were visualized by NMDS. (**B**) Microbiota difference between lower_BA and higher_BA at the family level. (**C**) Bacteria enriched in the fermentation contained key enzyme in each step of the butyric acid synthesis pathway. (**D**) Bar chart shows the differences in key enzymes involved in the butyric acid synthesis pathway between lower_BA and higher_BA. EC numbers were obtained from the KEGG database. lower_BA, lower butyric acid content; higher_BA, higher butyric acid content; NMDS, non-metric multidimensional scaling; *P < 0.05, **P < 0.01, ***P < 0.001.

The formation of butyric acid in silage is generally undesirable as it indicates that the silage has undergone clostridial fermentation rather than the more beneficial lactic acid fermentation. This type of fermentation not only leads to a loss in the nutritional value of the crude protein but also results in the production of odorous compounds and potentially harmful biogenic amines. The presence of butyric acid suggests extensive protein breakdown, which reduces the availability of essential amino acids necessary for the optimal nutrition of ruminants (14, 36, 37). The samples were categorized into two groups based on butyric acid content: the lower butyric acid content group (lower_BA; *n* = 7, butyric acid content <0.50 g/kg DM) and the higher butyric acid content group (higher_BA; *n* = 8, butyric acid content >5.00 g/kg DM). NMDS analysis revealed a significant difference in microbial composition between the lower_BA and higher_BA groups (Fig. 7A).

Further analysis revealed that microorganisms from the Lactobacillaceae, particularly *Lactiplantibacillus* (22, 38, 41), were notably enriched in the lower_BA group (Fig. 7B; Fig. S8). To further explore the functions of these differentially abundant bacteria, we analyzed the metabolic pathways of the microbiome between the two groups (Fig. 7C; Fig. S9). The key enzyme EC 1.3.1.44, which is responsible for the conversion of crotonoyl-CoA to butanoyl-CoA, showed significantly higher enrichment in the higher_BA group compared to the lower_BA group (Fig. 7D; Fig. S10). Notably, butanoyl-CoA is utilized exclusively as a precursor for butyric acid production in this study. The enzymes involved in converting butanoyl-CoA to butyric acid, specifically EC 2.8.3.8, EC 2.3.1.19, and EC 2.7.2.7, were found in higher abundance in the higher_BA group, potentially explaining the elevated levels of butyric acid observed in these samples.

## DISCUSSION

In this study, we conducted a systematic investigation of the classification and functional characteristics of the microbial community in alfalfa silage. Previous researchers have primarily employed high-throughput sequencing techniques to explore the microbial community structure during silage fermentation (2, 8, 11, 12, 14, 17, 21, 22, 30, 42). However, due to the complexity of bacterial communities in an ensiling environment, it is often challenging to capture rare but important microorganisms through direct sequencing (25, 43). This does not diminish the value of direct sequencing methods as direct sequencing without cultivation typically provides better coverage of phylogenetic diversity compared to culture-based approaches (44, 45). Combining uncultivated techniques with microbiological cultivation is a valuable complementary approach that allows for a better understanding of bacterial community diversity and unculturability (20, 23, 40). Furthermore, we should acknowledge the significance of comprehensive culture-based approaches for enriching diverse and particularly difficult-to-culture microorganisms since samples obtained from single or limited culture conditions are insufficient to reflect most features of cultivable communities (46, 47). It is vital to isolate key strains from the microbial community in the environment, as pure cultures of microorganisms continue to serve as the cornerstone for comprehensive studies on gene expression, protein function, and metabolic pathways in microbiology (48, 49). Various pretreatment methods have been demonstrated to enhance the diversity of microbial isolation and cultivation (23, 29, 50). In this study, the cultivation methods obtained nine potential novel strains, and eight were derived from hot or alcohol pretreatment, suggesting that different pretreatment methods can selectively amplify signals from specific taxa enriched under distinct culture conditions thereby enhancing the diversity of cultivable microorganisms. Similar phenomena have been observed in the microbiota of mice (40), pigs (24, 29), and human intestines (18, 23). Overall, in this study, we conducted a systematic large-scale microbial culture and high-throughput sequencing, which obtained a total of 7,225 ASVs, including 458 potential novel strains. This not only enhanced the richness and diversity of the silage microbial community but also shed light on the undiscovered potential novel microbial resources in the silage environment.

We should prioritize the microorganisms with shared functions, and characterizing their microbial communities based on their functional properties could facilitate the manipulation of these communities for specific purposes (51, 52). Through an in-depth high-throughput sequencing of the silage microbial community, we can further delineate the core microbiota and their functional attributes (17, 22, 30). Notably, some of the core-genera identified in this study overlapped with those previously discovered in whole-plant corn and oat silage research, suggesting that numerous factors driving community assembly may be common among different plant species (17, 31). Moreover, several of these core silage microorganisms exhibit beneficial properties for fermentation. For instance, *Lactiplantibacillus*, *Leuconostoc*, *Lentilactobacillus*, *Weissella*, and *Liquorilactobacillus* have been observed to inhibit the growth of detrimental microorganisms in fermentation environments (21, 22, 38). The establishment of a core-genera provides a valuable foundation for future research, which can leverage synthetic communities to elucidate the interactions between microorganisms and fermentation. In addition, it is necessary not only to determine the microbial assembly associated with silage fermentation but also to ascertain their stability before establishing their connection with acid production and antimicrobial properties, potentially enabling their utilization for further development.

Ammonia-N is a significant indicator of proteolysis during fermentation (53, 54). In the proteolysis pathway, since the deamination of amino acids can be catalyzed by only a handful of enzymes, glutamate deamination by glutamate dehydrogenase is central in amino acid catabolism (55, 56). The functional genomics enabled us to propose a catabolism pathway for amino acids to ammonia-N during ensiling. The pathway primarily comprises three distinct routes that result in the production of ammonia-N. Specifically, in the degradation pathway of glycine, it can be degraded to ammonia-N or transformed into serine by EC 2.1.2.1 or EC 2.1.2.10, respectively. The gene abundance of encoding EC 2.1.2.10 was higher during the conversion of glycine to serine. Conversely, in the pathway of ammonia-N production, the gene abundance of EC 2.1.2.1 was higher in the higher_AN group. Comparative analysis of the microbial revealed that Lactobacillaceae was significantly enriched in the serine synthesis pathway, but it was not present in the ammonia-N-producing pathway. This result suggested that Lactobacillaceae may promote serine synthesis from glycine to mitigate ammonia-N production. Butyric acid biosynthesis typically takes place during the reduction process of butanoyl-CoA, with EC 2.8.3.8 playing a crucial role as an enzyme in this process (57, 58). Both Bacillaceae and Enterobacteriaceae possess highly expressed genes associated with EC 2.8.3.8 production. Enterobacteriaceae is well known for its association with butyric acid production (32). Overall, these findings indicated the involvement of a diverse consortium of microbes in substrate degradation during silage fermentation. Further transcriptomic studies are necessary to validate these observations.

### Conclusions

In conclusion, this study integrated culturomics and metagenomics to provide valuable information for deeply understanding the microbiome composition and its potential function in silage fermentation. Our results identified the core microbes involved in silage fermentation, and most of the microbes can be recovered by culturomics including those with low abundance, which provide a useful reference for guiding the culture of specific microbes in silage fermentation. Importantly, we found that Enterobacteriaceae with the enzyme genes was related to the metabolic pathways of ammonia-N and butyric acid, supplementing a new perspective for understanding the relationship between the microbiome and nutrition degradation in silage fermentation. These findings significantly expand our understanding of the microbial composition and function associated with silage fermentation. This knowledge provides a foundational basis for the development of microbiome strategies, such as creating inoculants, aimed at enhancing the quality of fermentation.

## MATERIALS AND METHODS

### Sample collection

In 13 different farms across seven provinces in China (Hebei, Inner Mongolia, Tianjin, Henan, Shandong, Hunan, Shanxi), a total of 115 bales (one ton per bag) of alfalfa silage were collected, and 1-kg samples were taken from each bale. Detailed information regarding the collected samples can be found in Table S6, and detailed information on the locations is shown in Table S7. Using a sampler, samples were obtained from the upper, middle, and lower sections per alfalfa silage bale, respectively. Following thorough mixing, DNA extraction samples were immediately placed on dry ice and transported to the laboratory for further processing; culture samples were stored at 4°C on ice until isolation and cultivation; after transportation back to the laboratory, samples for determining fermentation quality and nutritional components were promptly analyzed.

### Determine fermentation quality

The 20-g sample was thoroughly mixed with 180 mL of sterile water, followed by homogenization at 4°C and 100 r/min for a duration of approximately 3 h. The resulting filtrate was collected through four layers of sterile gauze and a 0.45-µm filter membrane. A portion of this filtrate was utilized for direct measurement of pH value (PHS-3C, INESA Scientific Instrument, Shanghai, China) and ammonia-N content with phenol–sulfuric acid colorimetric method (59). Another portion filtration was performed through a 0.22-µm filter membrane before being analyzed via high-performance liquid chromatography to determine organic acid content (60) (lactic acid, acetic acid, propionic acid, butyric acid) (Showa Denko K.K., Kawasaki Japan).

Approximately 200 g of sample was dried at 60°C for 48 h to a constant weight, and then, the dry matter content was determined. After grinding the dried samples through a 1-mm sieve, their nutritional components were analyzed. Total nitrogen content was measured using the Kjeldahl method (FOSS Kjeltec 2300), and crude protein content in the samples was calculated by multiplying total nitrogen by 6.25 (61). Neutral detergent fiber and acid detergent fiber contents (62) were determined using an Ankom 2000 Fiber Analyzer (Ankom Technology, Fairport, NY), based on the method described by Van Soest et al. Water-soluble carbohydrate content (63) was measured using anthrone colorimetry as described by Murphy in 1958.

### Microbial isolation and cultivation

To enhance the diversity of cultured microorganisms and reduce workload, we categorized the samples into eight groups based on their geographical locations and collection times. Each group underwent microbial isolation and cultivation thrice, resulting in a total of 24 operations for isolation and cultivation. The 48 methods for microbial cultivation comprised 12 culture media (Table S1) and four pretreatment methods (Table S2), including alcohol pretreatment and heat treatment techniques derived from previous studies (27, 40, 50). The gas composition within the anaerobic workstation consisted of 80% N_2_, 10% CO_2_, and 10% H_2_.

In the anaerobic workstation, the samples were divided into four groups. The first group was subjected to pretreatment with PBS solution at 180 rpm for 60 min (PBS). The second group underwent pretreatment with 70% alcohol at 180 rpm for 40 min (Alcohol). The third group received pretreatment with PBS solution at 70°C for 20 min (HotPBS). The fourth group was subjected to pretreatment with 70% alcohol at 70°C for 20 min (HotAlcohol). Subsequently, the large insoluble particles in suspension were removed using a cell strainer (BD Falcon, USA). The suspension was then sequentially diluted in gradients of dilution ranging from 10^−1^ to 10^−6^. Under gas conditions consisting of 80% N_2_, 10% CO_2_, and 10% H_2_, each gradient of dilution liquid (100 µL) was spread onto various culture media and incubated under aerobic or anaerobic conditions at temperatures of either 30°C or 37°C. The single colonies appearing on the agar plates after incubation for 2, 3, 4, and 7 days were picked, based on morphological indicators such as colony size, shape, color, presence of halo formation, and clarity or fuzziness of colony outlines on each culture medium. Morphology and corresponding cultivation conditions for each colony were recorded. To avoid collecting duplicate colonies repeatedly at different times, once a colony was picked, it would be immediately marked on the backside of the petri dish accordingly. The picked colonies were inoculated onto respective solid cultures to observe their growth rate, further purified (dilution streaks), and expanded in cultivation. Then, single colonies were collected from each culture medium, resuspended in 2 µL of NaOH/SDS lysis buffer, diluted with 100 µL of deionized water, and lysed under 95°C for 30 min (27 F: 5′-AGAGTTTGATCCTGGCTCAG-3′; 1492 R: 5′-GGTTACCTTGTTACGACTT-3′). The 16S RNA gene sequences amplified by PCR were identified through Sanger sequencing, conducted by Beijing Liuhe Huada Genomics Institute. Using EZBioCloud and the NCBI 16S ribosomal RNA sequence database, we conducted Blast analysis on the microbial 16S rRNA gene sequences cultured in our study (date: 11 May 2023; sequence count: 2,959). The most optimal match for each sequence was determined based on the lowest e-value. Subsequently, a phylogenetic tree of the strains was constructed using MEGA (v11) employing linkage methods, and the results were visualized using Interactive Tree of Life online software (iTOL; v6.7.6). Following purification and expansion in culture, individual colonies were preserved at −80°C with 20% glycerol for subsequent analysis.

### The 16S rRNA gene amplicon sequencing and analysis

To gain a deeper understanding of the microbial community composition in alfalfa silage, we utilized the FastDNASPIN for soil kit (MP Biomedicals, Solon, USA) to amplify the 16S rRNA gene from prokaryotic DNA in a total of 115 samples of alfalfa silage (64). The concentration and purity of DNA were determined using NanoDrop 2000 (Thermo Scientific, USA). The full-length 16S rRNA gene was amplified through PCR utilizing the following barcoded primers: 27 F (5′-AGRGTTTGATYNTGGCTCAG-3′) and 1,492 R (5′- TASGGHTACCTTGTTASGACTT-3′). Following 27 cycles of amplification, PCR products were purified using AMPure PB beads (Pacific Biosciences, CA, USA) and quantified with Qubit 4.0 (Thermo Fisher Scientific, USA). Subsequently, as per the PacBio manual guidelines, SMRT bell prep kit 3.0 (Pacific Biosciences, CA, USA) was employed to generate sequencing libraries. Sequencing was carried out on a Pacbio Sequel IIe System (Pacific Biosciences, CA, USA). Circular consensus sequencing was then performed using SMRT Link (v11.0) to obtain high-fidelity HiFi reads from the raw data. DADA2 within the Qiime2 pipeline (v2020.2) was utilized for denoising optimization of HiFi reads with recommended parameters and assigning amplicon sequence variants (ASVs) at single-nucleotide resolution based on error profiles within each sample (65). A total count of 4,266 ASVs was obtained. All ASV sequences underwent analysis via Blast against EZBioCloud and NCBI’s ribosomal RNA sequence database for bacterial isolation culture analysis as well as identification purposes.

### Metagenomic sequencing, processing, and analysis

To further investigate the functions of microorganisms in alfalfa silage, genomic DNA was extracted from 30 samples of alfalfa silage using the aforementioned method. The purity and concentration of the extracted DNA were determined using TBS-380 and NanoDrop 2000, respectively. Additionally, the quality assessment of the extracted DNA was conducted through electrophoresis on a 1% agarose gel. Subsequently, a DNA library was constructed utilizing NEXTFLEX Rapid DNA-Seq (Bioo Scientific, USA), followed by sequencing on an Illumina NovaSeq platform (Illumina, USA). A total of 0.31-Tbp raw reads were generated from this experiment. To preprocess the original sequencing data, fastp software (https://github.com/OpenGene/fastp) was employed for adapter sequence removal as well as the elimination of low-quality bases, N bases, and short sequences (66). Specifically, (i) adapter sequences at both ends of reads were trimmed, and (ii) reads with lengths less than 50 bp or average quality values lower than 20 or containing N bases were discarded. The resulting trimmed reads were aligned against host DNA sequences using BWA software (v0.7.9), and contaminating reads with high similarity were removed to obtain clean reads totaling 0.30 Tbp (67). Assembly based on different sequencing depths was performed using Megahit software, employing the succinct de Bruijn graph method with iterative assembly starting from small k-mers up to large k-mers (68). In the process of assembly, contigs with a minimum length of 300 bp were selected as the final assembly result, resulting in a total of 10,974,927 contigs with a combined length of 8.26 Gbp. The selected contigs were then subjected to open reading frame (ORF) prediction using Prodigal software (69). Gene sequences with nucleotide lengths equal to or greater than 100 bp were chosen and translated into amino acid sequences. CD-HIT (v4.6.1) was utilized to construct a non-redundant gene catalog based on sequence consistency and coverage over than 90%, where the longest gene sequence was designated as the representative for each species, resulting in a total of 3.57 million non-redundant genes (unigenes) (70). Subsequently, SOAPaligner (v2.21) was employed for aligning high-quality reads from each sample against the non-redundant gene catalog (with an identity threshold set at 95%), enabling quantification of gene abundance information within corresponding samples (71). Next, we utilized Diamond (v0.8.35) to perform amino acid sequence comparisons of the non-redundant gene set against the NR database (using BLASTP with an expected value e-value threshold of 10^−5^) (72). Subsequently, species annotations were obtained by referencing the taxonomic information database corresponding to the NR library. The abundance of each species was then calculated based on their respective gene abundances. To further acquire functional insights into genes, we employed diamond to compare the amino acid sequences of unigene sets with KEGG databases (using BLASTP with an expected value e-value threshold of 10^−5^. This allowed us to obtain KEGG functions for each gene and calculate the abundance of corresponding functional categories based on KO, Pathway, EC, and Module-associated gene abundances. Finally, we utilized hmmscan tool in the CAZy database to compare amino acid sequences from unigenessets against the CAZy database (using a comparison parameter setting with an expected value e-value threshold of 10^−5^), enabling us to obtain carbohydrate-active enzyme annotation information for each gene and subsequently calculate its abundance based on carbohydrate-active enzyme-associated gene abundances.

### Statistical analysis

The analysis methods in this study were conducted using IBM SPSS v21.0 (SPSS Inc., Chicago, IL, USA). Fermentation quality and nutritional components were processed in Excel 2019 and visualized using GraphPad Prism 8 to generate violin plots. All data had a minimum of three replicates. Phylogenetic trees based on the construct neighbor-joining tree method for analyzing evolutionary relationships between strains were constructed using MEGA.11 software and then visualized with the online software Interactive Tree of Life (iTOL; v6.7.6). Spearman correlation analysis was employed to explore the interactions between microorganisms and Cazys, as well as key enzymes in nitrogen metabolism and butyric acid formation pathways among different bacterial species, with visualization through heatmaps. Boxplots and bar graphs displaying median values and all valid data were generated using GraphPad Prism 8.

## Data Availability

The raw data of 16S rRNA gene amplicons are deposited in NCBI by under accession number PRJNA983055. All the metagenomic data obtained in this study are available at NCBI under project number PRJNA1029236. The 16S rRNA gene sequences of alBM are available under NCBI ID PRJNA983448.

## SUPPLEMENTAL MATERIAL

Supplemental Figures msystems.00682-24-s0001.docx Figures S1-S10.

Supplemental Tables msystems.00682-24-s0002.docx Tables S1 to S7.

ASM does not own the copyrights to Supplemental Material that may be linked to, or accessed through, an article. The authors have granted ASM a non-exclusive, world-wide license to publish the Supplemental Material files. Please contact the corresponding author directly for reuse.
