# Supplementary material for: Multi-omics analysis reveals the core microbiome and biomarker for nutrition degradation in alfalfa silage fermentation
Source: mSystems. 2024 Oct 23;9(11):e00682-24. doi: 10.1128/msystems.00682-24 (PMC11575373; doi:10.1128/msystems.00682-24)
Supplement: Supplemental Figures — Figures S1-S10. [file msystems.00682-24-s0001.docx]

Supplementary figures for

**Multi-omics analysis reveals the core microbiome and biomarker for nutrition degradation in** **alfalfa silage fermentation**

Yuan Wang^1,2^, Yunlei Sun^1^, Kexin Huang^1^, Yu Gao^1^, Yufan Lin^1^, Baojie Yuan^1^, Xin Wang^1^, Gang Xu^1^, Luiz Gustavo Nussio^4^, Fuyu Yang^1,2,3^, Kuikui Ni^1^

Address correspondence to Fuyu Yang, [yfuyu@126.com](mailto:yfuyu@126.com), Kuikui Ni, [nikk@cau.edu.cn](mailto:nikk@cau.edu.cn).

^1^ College of Grassland Science and Technology, China Agricultural University, Beijing 100193, China

^2^ Frontier Technology Research Institute of China Agricultural University in Shenzhen, Shenzhen 518000, China

^3^ College of Animal Science, Guizhou University, Guiyang 550025, China

^4^ Department of Animal Science, University of São Paulo, Piracicaba 13418-900, Brazil

**Contents**

**Supplementary Figures**

**Supplementary Figure 1 |** The correlation analysis between pH and butyric acid content.

**Supplementary Figure 2 |** The workflow for the large-scale cultivation and characterization of the silage microbiota.

**Supplementary Figure 3 |** The taxonomic diversity of bacteria in the alBM.

**Supplementary Figure 4 |** The microbiota difference between the direct sequencing and alBM at the genus (A) and species (B) level.

**Supplementary Figure 5 |** The microbiota difference between Lower_AN and Higher_AN at the genus level.

**Supplementary Figure 6 |** The number of enzyme genes involved in the ammonia-N metabolic pathway.

**Supplementary Figure 7 |** The relationship between the key enzymes and microorganisms involved in the ammonia-N metabolism pathway.

**Supplementary Figure 8 |** The microbiota difference between Lower_BA and Higher_BA at the genus level.

**Supplementary Figure 9 |** The number of enzyme genes involved in the butyric acid synthesis pathway.

**Supplementary Figure 10 |** The relationship between the key enzymes and microorganisms involved in the butyric acid synthesis pathway.

**
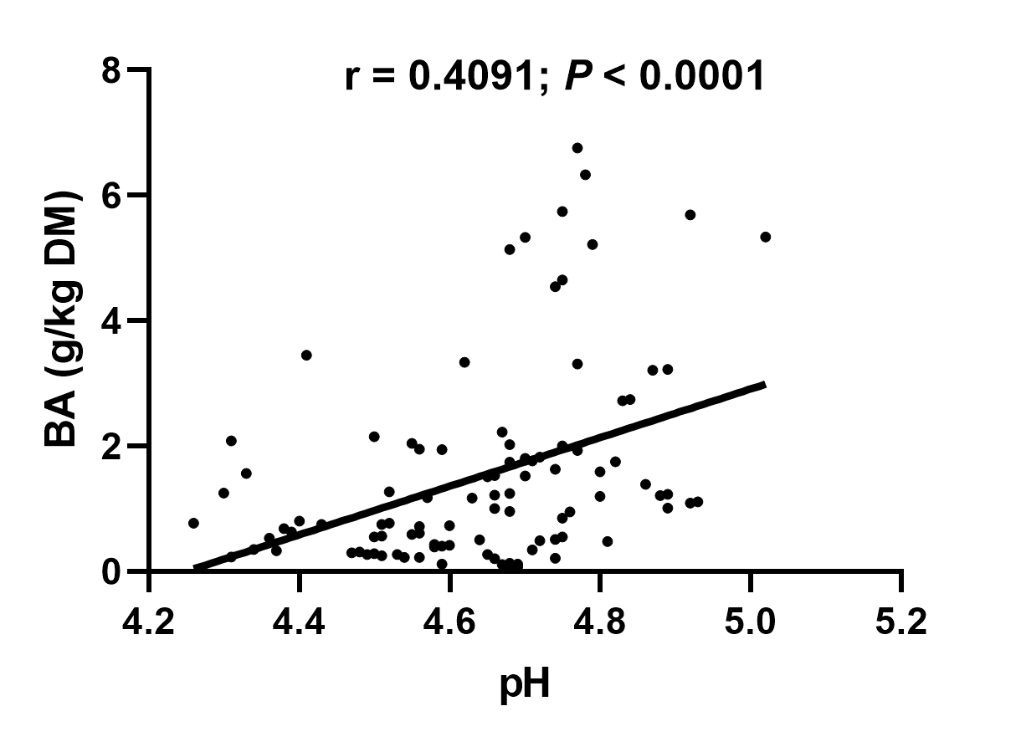
**

**Supplementary Figure 1. The correlation analysis between pH and butyric acid content**

**
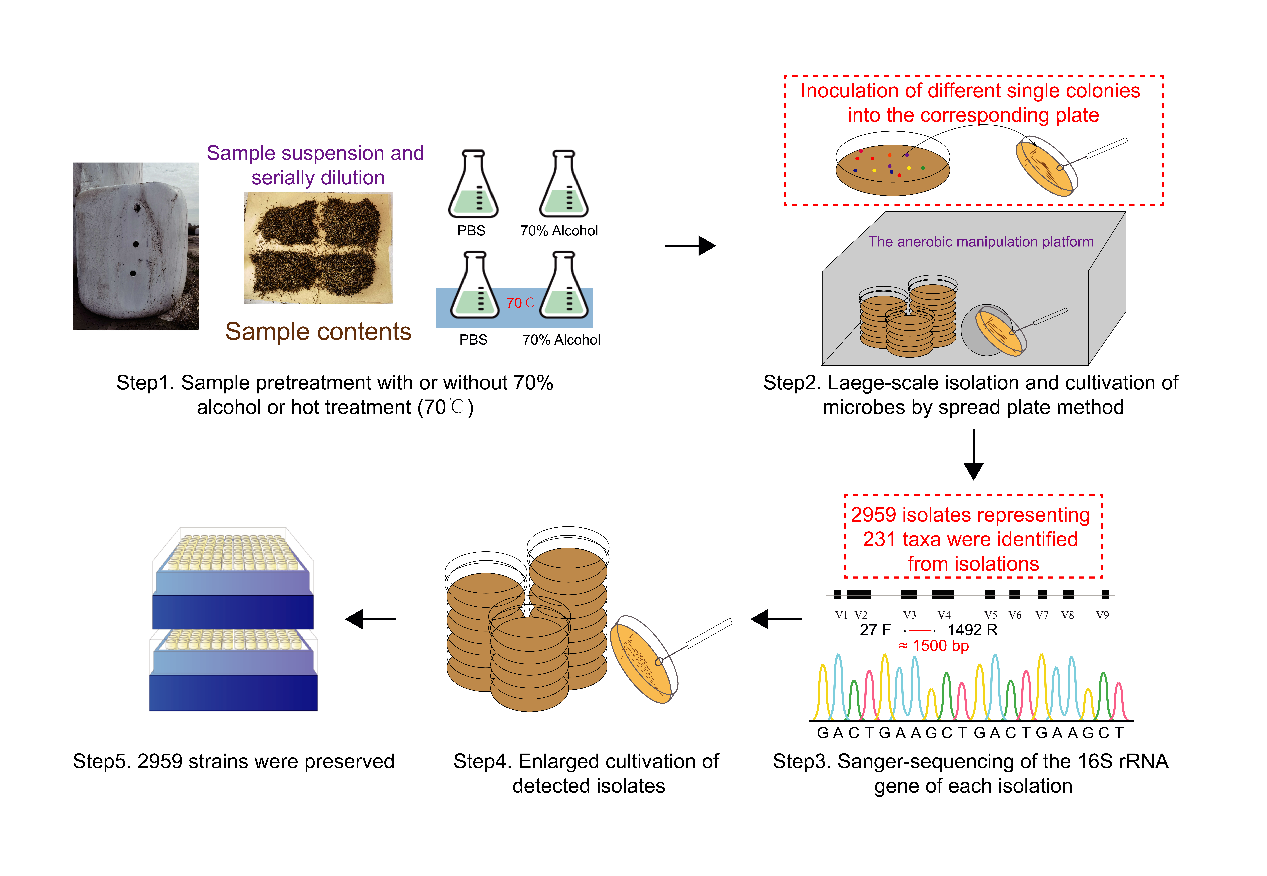
**

**Supplementary Figure 2. The workflow for the large-scale cultivation and characterization of the silage microbiota.** The working steps were numbered from Steps 1 to 5. The main outcomes were shown on top of the panel and in a red dashed box.


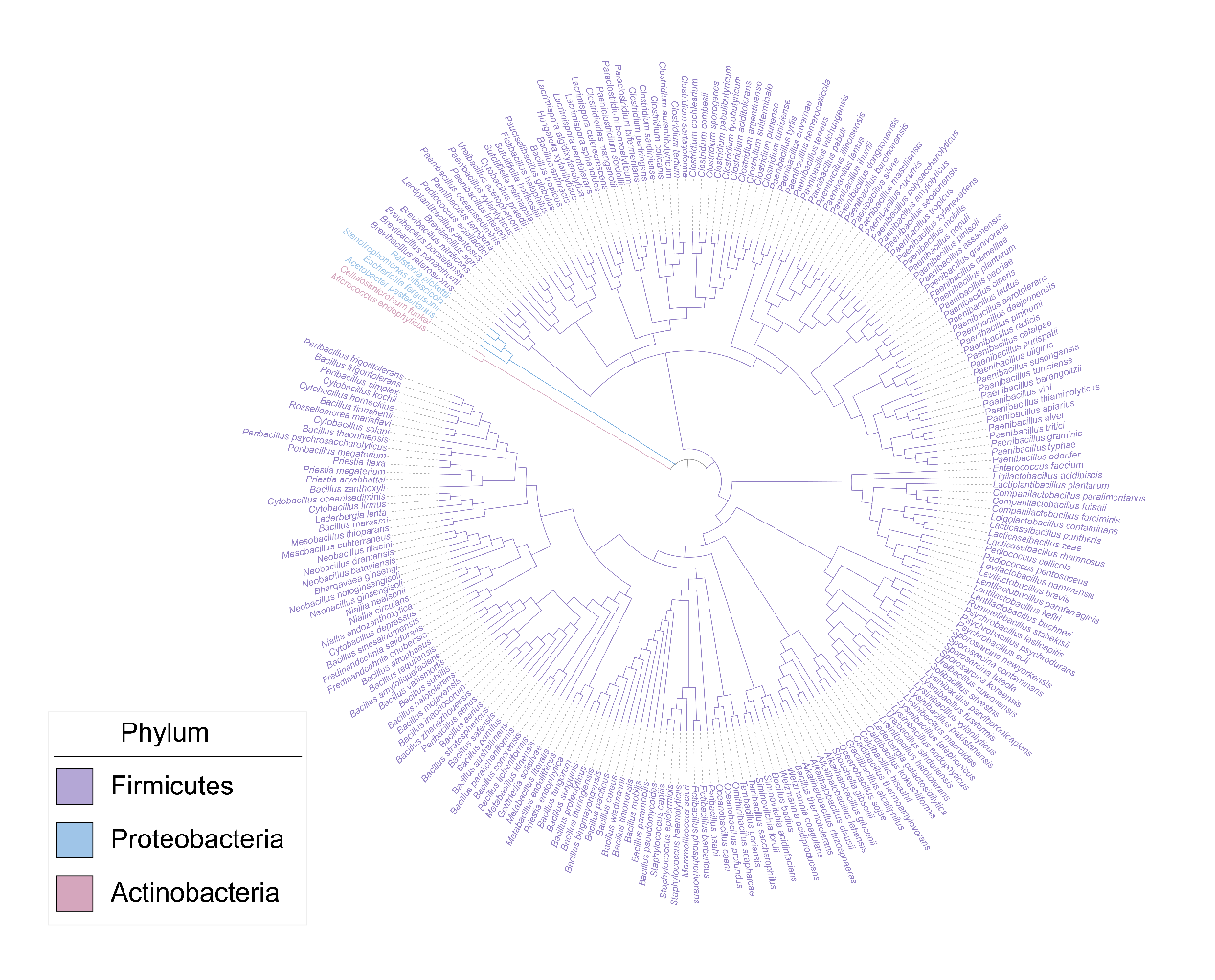


**Supplementary Figure 3. The taxonomic diversity of bacteria in the alBM.** The background is color-coded according to phyla level.


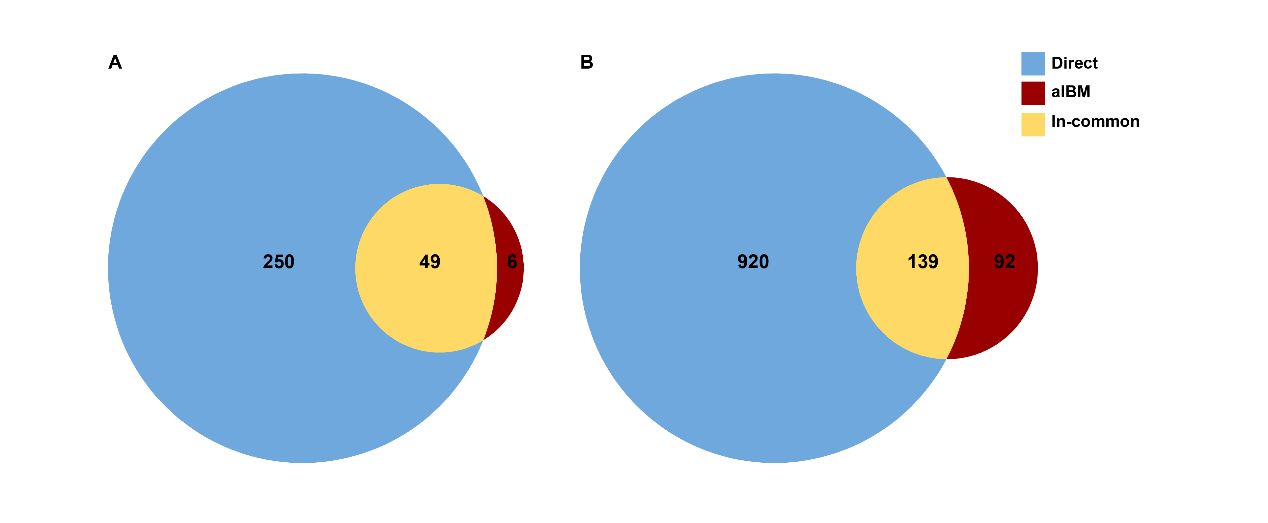


**Supplementary Figure 4. The microbiota difference between the direct sequencing and alBM at the genus (A) and species (B) level.**


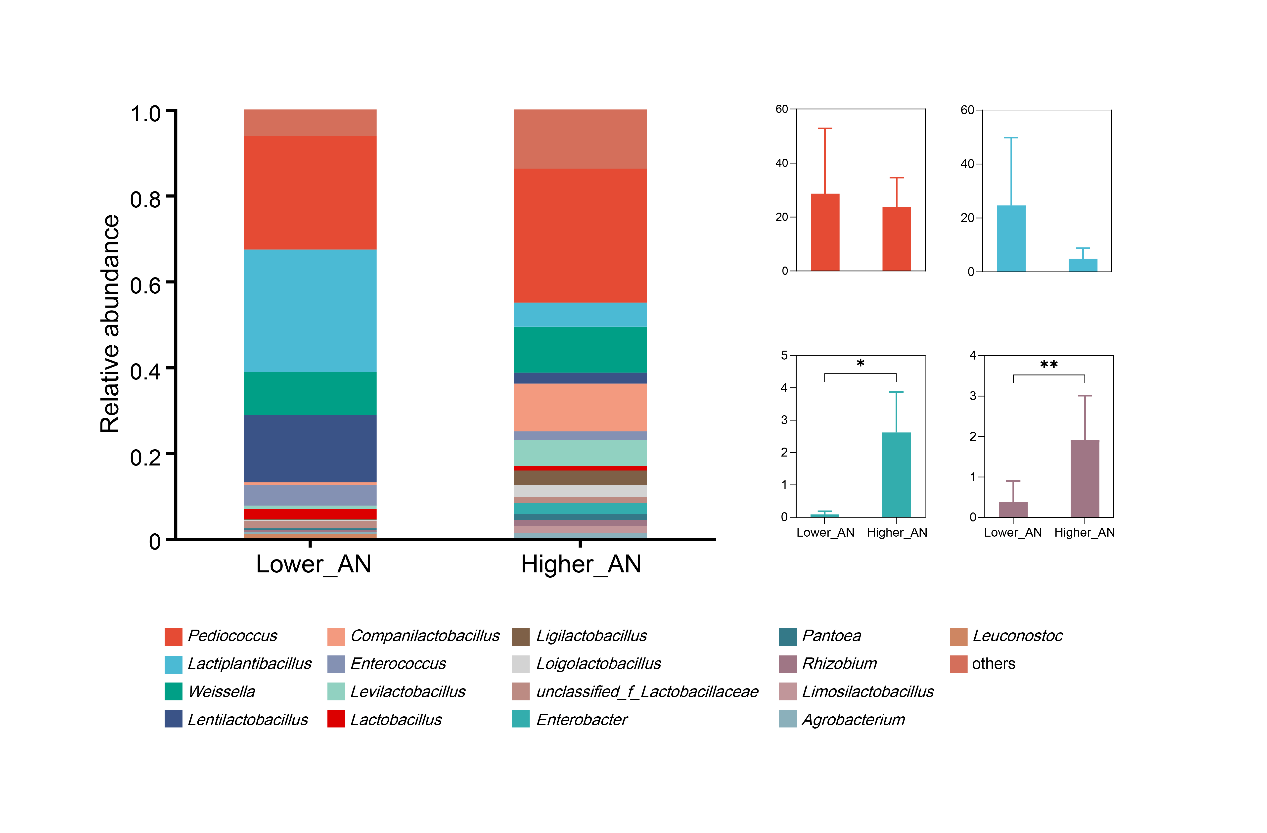
**Supplementary Figure 5. The microbiota difference between Lower_AN and Higher_AN at the genus level.** Lower_AN: lower ammonia-N content; Higher_AN: higher ammonia-N content. **P* < 0.05, ***P* < 0.01, ****P* < 0.001.


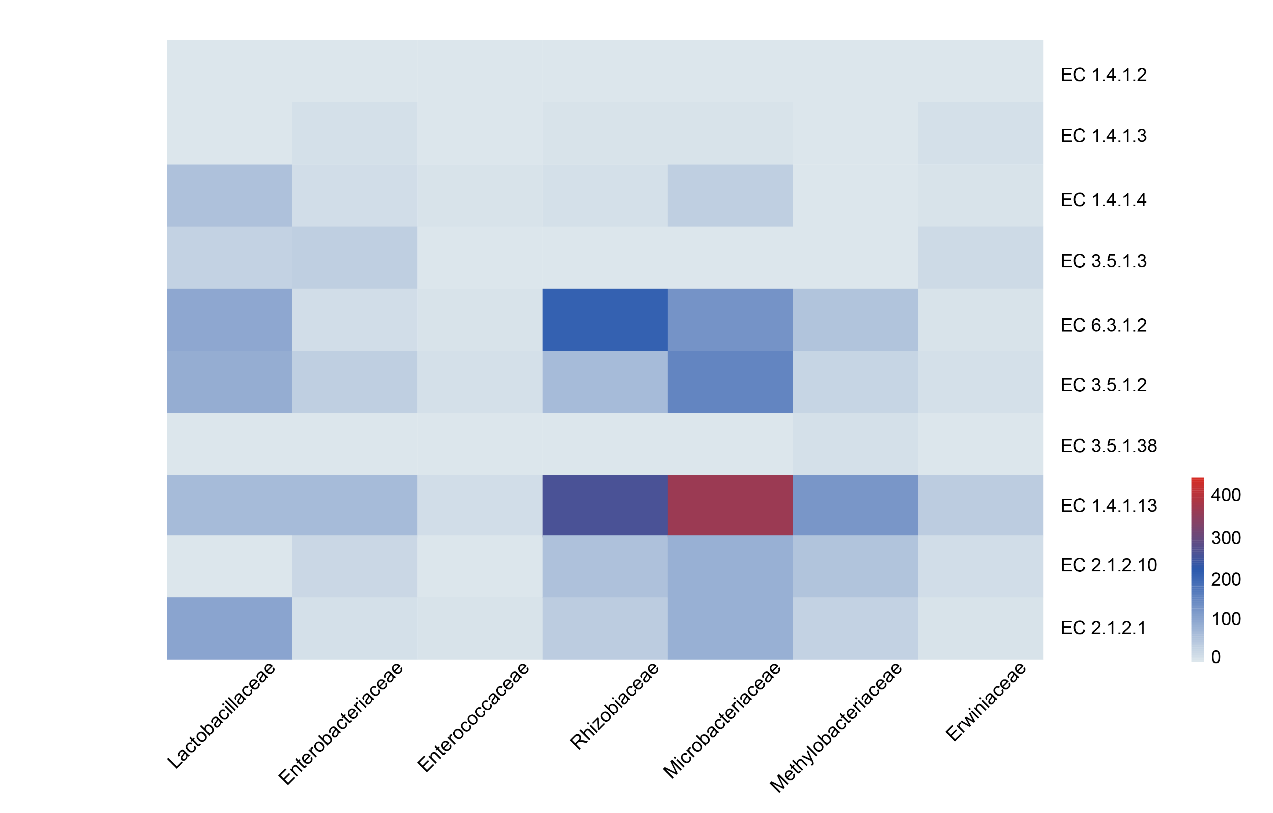
**Supplementary Figure 6. The number of enzyme genes involved in the ammonia-N metabolic pathway.**


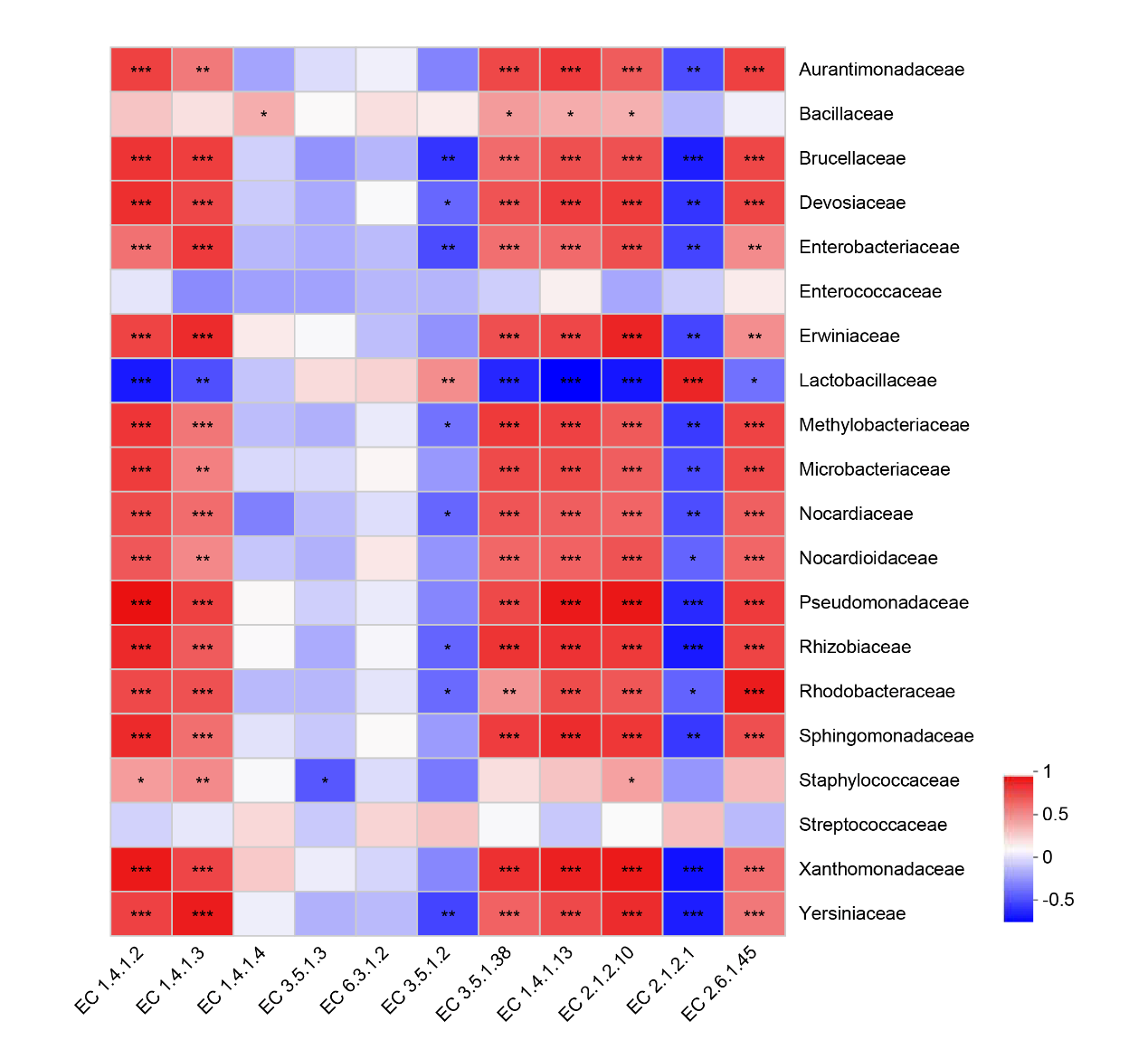


**Supplementary Figure 7. The relationship between the key enzymes and microorganisms involved in the ammonia-N metabolism pathway.** **P* < 0.05, ***P* < 0.01, ****P* < 0.001.


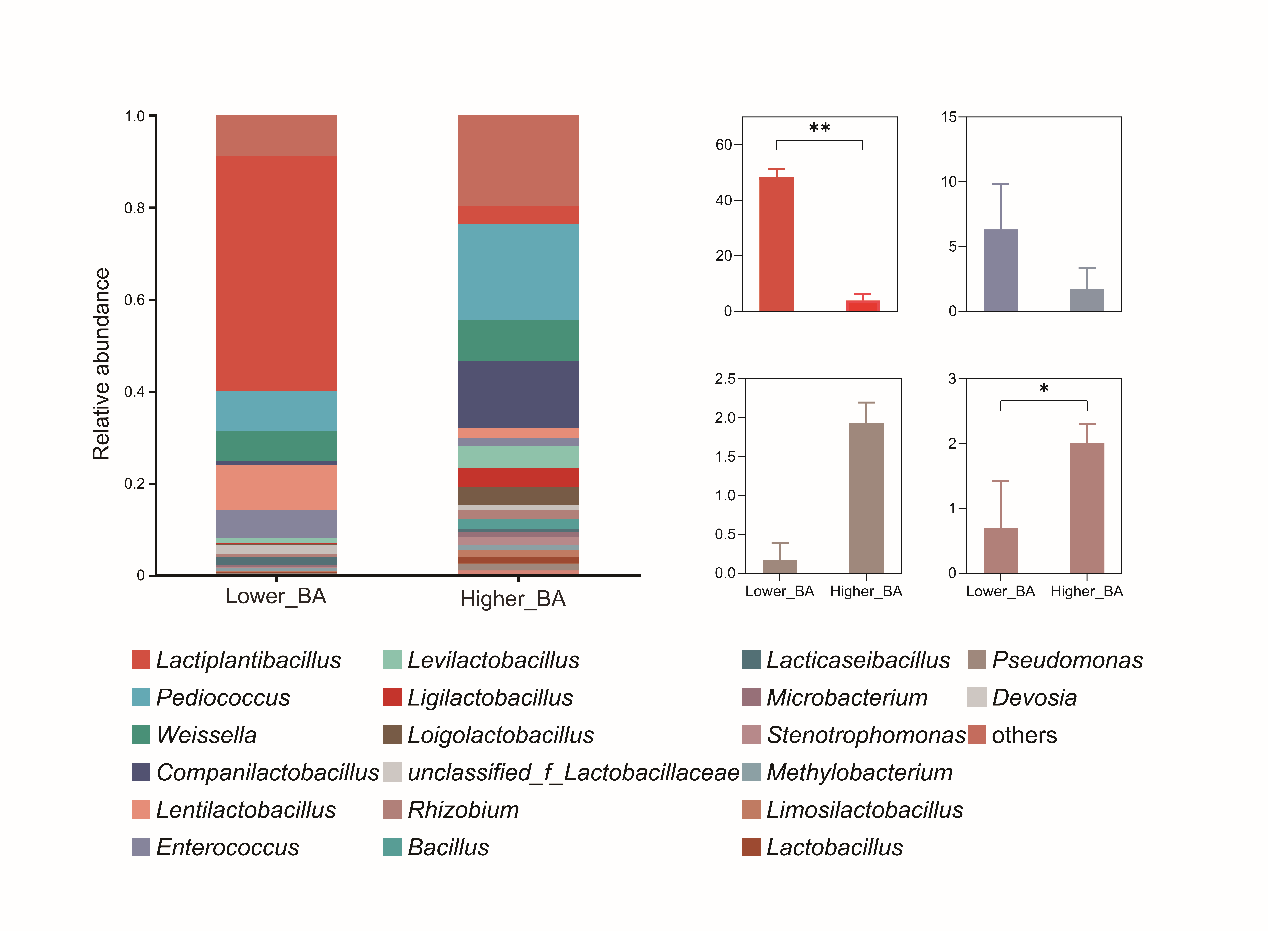


**Supplementary Figure 8. The microbiota difference between Lower_BA and Higher_BA at the genus level.** Lower_BA: lower butyric acid content; Higher_BA: higher butyric acid content. **P* < 0.05, ***P* < 0.01, ****P* < 0.001.


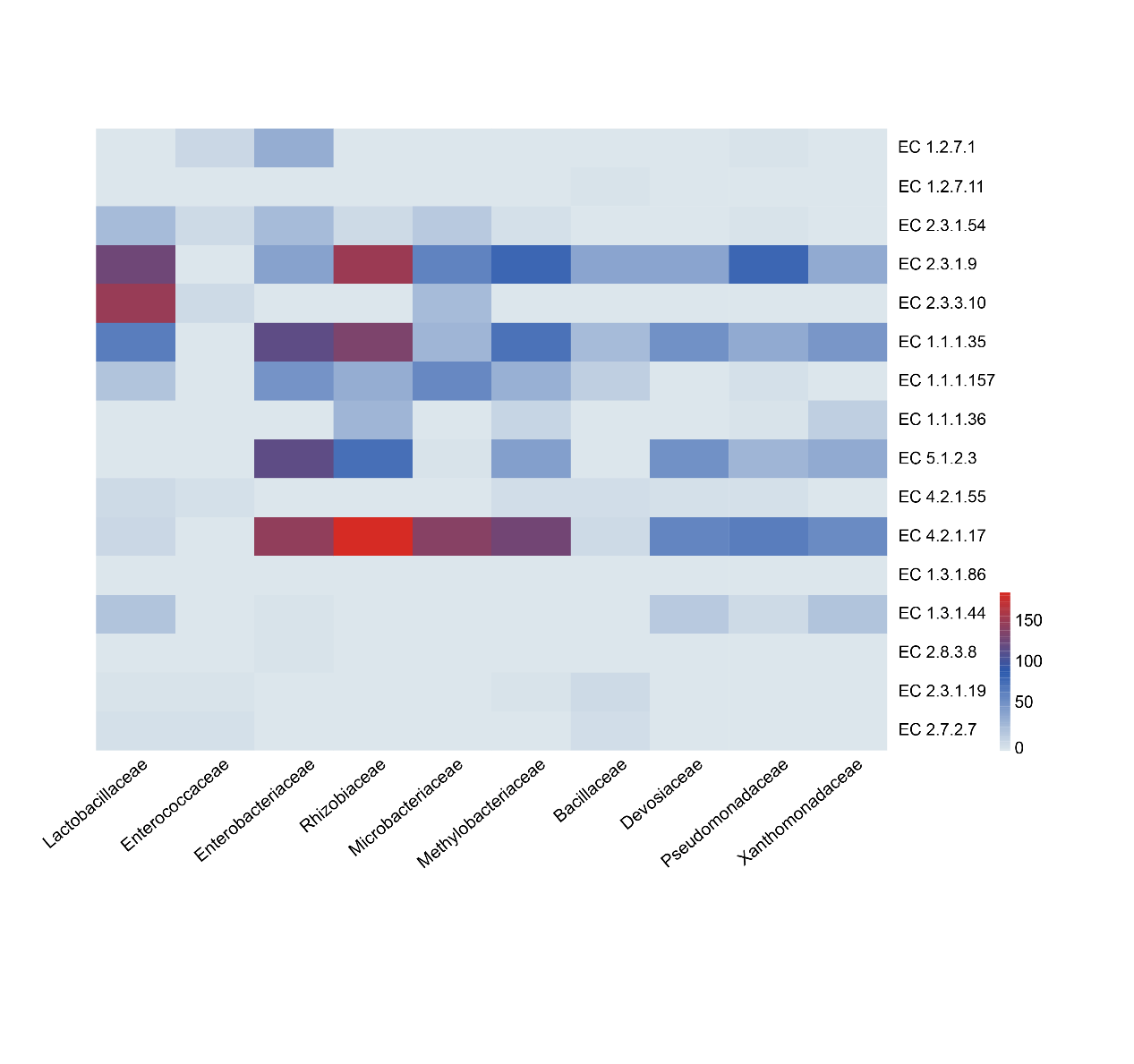


**Supplementary Figure 9. The number of enzyme genes involved in the butyric acid synthesis pathway.**


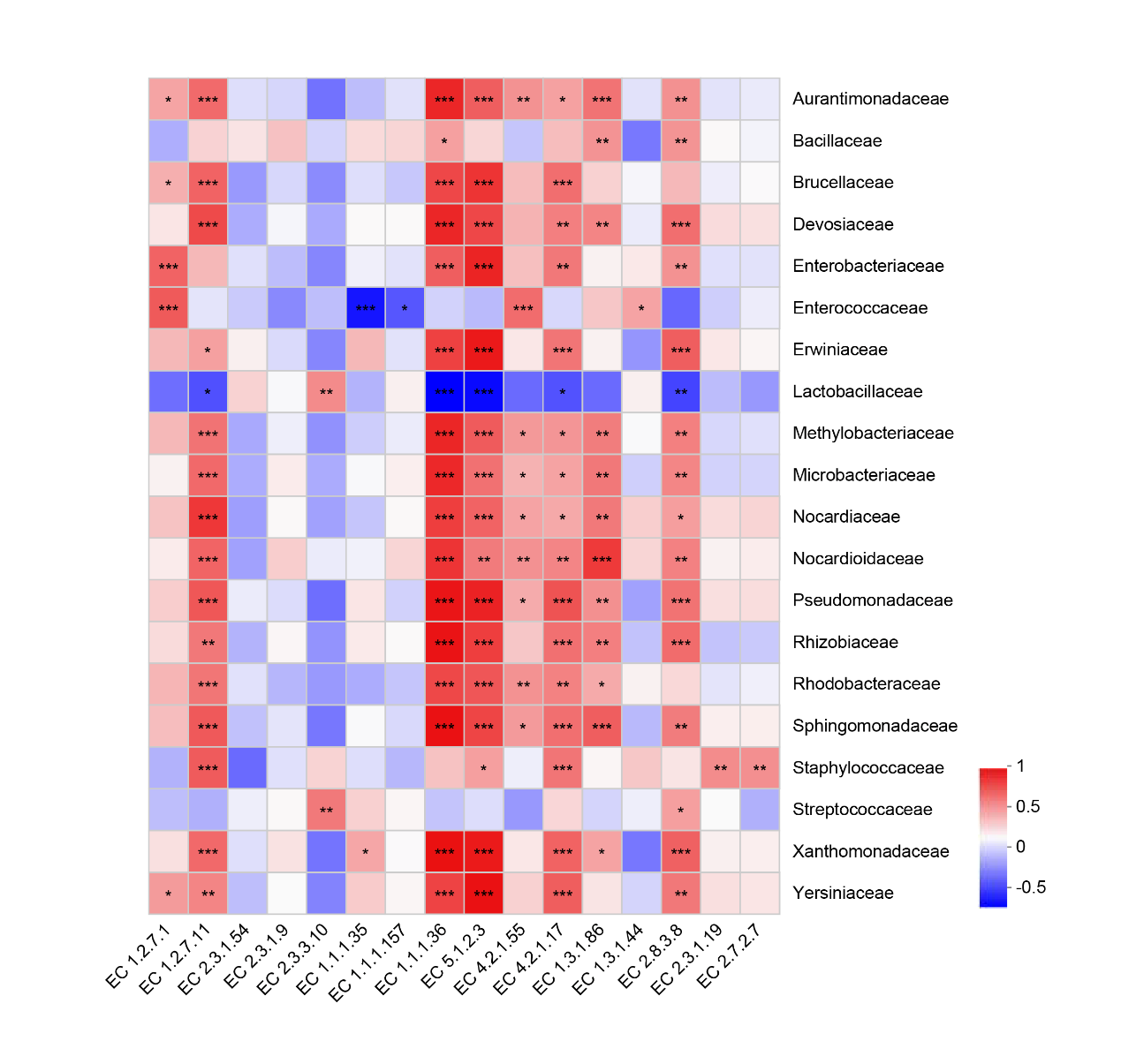


**Supplementary Figure 10. The relationship between the key enzymes and microorganisms involved in the butyric acid synthesis pathway.** **P* < 0.05, ***P* < 0.01, ****P* < 0.001.
