# Supplementary material for: Multi-omics analysis reveals the core microbiome and biomarker for nutrition degradation in alfalfa silage fermentation
Source: mSystems. 2024 Oct 23;9(11):e00682-24. doi: 10.1128/msystems.00682-24 (PMC11575373; doi:10.1128/msystems.00682-24)
Supplement: Supplemental Tables — Tables S1 to S7. [file msystems.00682-24-s0002.docx]

| **Table S1. The media and culture conditions used for large-scale microbe isolation** | | | | |
| --- | --- | --- | --- | --- |
| **Culture conditions** | **Base medium** | **Abbreviation** | **Incubation temprature** | **Incubation Gaseous environment** |
| 1 | Luria Bertani Medium | LB Medium | 30℃ | Aerobic |
| 2 | TSA Medium | TSA Medium | 30℃ | Aerobic |
| 3 | Nutrient Broth Medium | NB Medium | 30℃ | Aerobic |
| 4 | YCFA Medium | YCFA Medium | 30℃ | Anaerobic |
| 5 | MRS Medium | MRS Medium | 37℃ | Anaerobic |
| 6 | LBS Medium | LBS Medium | 37℃ | Anaerobic |
| 7 | M17 Medium | M17 Medium | 37℃ | Anaerobic |
| 8 | Eosin Methylene Blue Medium | EMB Medium | 30℃ | Aerobic |
| 9 | BLB Medium | BLB Medium | 30℃ | Aerobic |
| 10 | Differential Reinforced Clostridial Agar | DRCA Medium | 37℃ | Anaerobic |
| 11 | Ferric Sulfite Agar Medium | YLS Medium | 37℃ | Anaerobic |
| 12 | Reinforced Clostridium Medium | RCM Medium | 37℃ | Anaerobic |

| **Table S2. The pretreatments used for large-scale alfalfa silage microbe isolation** | | | | |
| --- | --- | --- | --- | --- |
| **Methods** | **Buffer or solution** | **Filteration** | **Temprature** | **Gaseous environment** |
| 1 | PBS | 70 μm | 37°C (60 min) | Anaerobic |
| 3 | 70% Alcohol | 70 μm | 37°C (40 min) | Anaerobic |
| 2 | PBS | 70 μm | 70°C (20 min) | Anaerobic |
| 4 | 70% Alcohol | 70 μm | 70°C (20 min) | Anaerobic |

| **Table S3. The identification results for alBM and Direct** | | | | | |
| --- | --- | --- | --- | --- | --- |
|  |  | **NCBI Blast** | | **Local Blast** | |
| **Direct ID** | **NCBI accession** | **16S rRNA**  **gene sequence**  **identity (%)** | **Taxonomy** | **alBM ID** | **Local identity (%)** |
| ASV9 | NR_112693.1 | 97.288 | *Ligilactobacillus acidipiscis* | Hun1-MRS-2 | 99.86 |
| ASV13 | NR_115953.1 | 96.98 | *Priestia aryabhattai* | Hn1-EMB-17 | 100 |
| ASV21 | NR_114742.1 | 98.418 | *Enterococcus faecium* | Nm2-YCFA-2 | 100 |
| ASV32 | NR_114625.1 | 98.604 | *Paenibacillus turicensis* | Hn2-TSA-9 | 98.87 |
| ASV46 | NR_114625.1 | 98.63 | *Paenibacillus turicensis* | Hn1-RCM-9 | 99.35 |
| ASV52 | NR_165702.1 | 98.086 | *Paenibacillus seodonensis* | Hun7-M17-3 | 97.94 |
| ASV53 | NR_113906.1 | 95.28 | *Enterococcus mundtii* | Nm2-YLS-1 | 99.02 |
| ASV57 | NR_113332.1 | 98.443 | *Lacticaseibacillus rhamnosus* | Nm3-LB-1 | 97.44 |
| ASV61 | NR_114398.1 | 98.433 | *Companilactobacillus farciminis* | Sx3-MRS-15 | 100 |
| ASV63 | NR_042243.1 | 97.297 | *Levilactobacillus hammesii* | Hb3-MRS-9 | 98.65 |
| ASV65 | NR_181100.1 | 98.658 | *Companilactobacillus pabuli* | Hun4-LBS-4 | 99.86 |
| ASV66 | NR_181100.1 | 97.942 | *Companilactobacillus pabuli* | Sx3-MRS-3 | 99.93 |
| ASV69 | NR_041293.1 | 98.434 | *Lentilactobacillus buchneri* | Sx2-DRCA-12 | 100 |
| ASV79 | NR_114398.1 | 96.376 | *Companilactobacillus farciminis* | Sx3-MRS-15 | 99.86 |
| ASV80 | NR_136785.1 | 97.453 | *Lactiplantibacillus plajomi* | Sx1-DRCA-11 | 98.81 |
| ASV107 | NR_043095.1 | 97.789 | *Liquorilactobacillus oeni* | Sx2-DRCA-12 | 100 |
| ASV130 | NR_041293.1 | 97.981 | *Lentilactobacillus buchneri* | Sx2-DRCA-12 | 100 |
| ASV136 | NR_118446.1 | 98.33 | *Niallia circulans* | Sd1-NB-32 | 99.57 |
| ASV157 | NR_117973.1 | 96.391 | *Companilactobacillus futsaii* | Hb3-MRS-6 | 99.01 |
| ASV180 | NR_116238.1 | 98.581 | *Levilactobacillus brevis* | Hb1-LBS-7 | 100 |
| ASV201 | NR_041830.1 | 98.042 | *Leuconostoc fallax* | Hle2-NB-16 | 100 |
| ASV214 | NR_113338.1 | 98.558 | *Lactiplantibacillus plantarum* | Sx1-DRCA-11 | 99.93 |
| ASV226 | NR_113338.1 | 98.707 | *Lactiplantibacillus plantarum* | Sx1-DRCA-11 | 99.86 |
| ASV229 | NR_041294.1 | 96.391 | *Lentilactobacillus parabuchneri* | Sx2-DRCA-12 | 100 |
| ASV235 | NR_114365.1 | 98.613 | *Loigolactobacillus iwatensis* | Nm3-DRCA-16 | 100 |
| ASV239 | NR_102783.2 | 97.502 | *Bacillus subtilis* | Sd1-LB-15 | 100 |
| ASV240 | NR_042337.1 | 98.043 | *Bacillus altitudinis* | Hn2-MRS-6 | 100 |
| ASV241 | NR_181236.1 | 98.443 | *Bacillus rugosus* | Hun4-NB-8 | 100 |
| ASV242 | NR_113945.1 | 97.153 | *Bacillus safensis* | Hun1-BLB-1 | 100 |
| ASV243 | NR_102783.2 | 97.732 | *Bacillus subtilis* | Hun4-RCM-23 | 100 |
| ASV244 | NR_180225.1 | 98.707 | *Calidifontibacillus erzurumensis* | Hle1-BLB-21 | 100 |
| ASV251 | NR_112693.1 | 98.806 | *Ligilactobacillus acidipiscis* | Hun1-MRS-2 | 100 |
| ASV256 | NR_180225.1 | 97.563 | *Calidifontibacillus erzurumensis* | Hun4-RCM-21 | 100 |
| ASV260 | NR_024931.1 | 98.443 | *Bacillus spizizenii* | Hun7-RCM-13 | 98.7 |
| ASV262 | NR_029230.1 | 98.707 | *Clostridium beijerinckii* | Hun7-TSA-20 | 97.62 |
| ASV264 | NR_112693.1 | 97.556 | *Ligilactobacillus acidipiscis* | Sd1-MRS-1 | 99.93 |
| ASV267 | NR_102783.2 | 94.089 | *Bacillus subtilis* | Sd1-LB-15 | 99.93 |
| ASV269 | NR_148787.1 | 96.379 | *Bacillus australimaris* | Hun1-YCFA-2 | 99.93 |
| ASV271 | NR_113265.1 | 97.352 | *Bacillus subtilis* | Hle2-YLS-5 | 99.79 |
| ASV272 | NR_115064.1 | 98.685 | *Peribacillus frigoritolerans* | Hn1-BLB-23 | 100 |
| ASV276 | NR_113777.1 | 98.111 | *Neobacillus niacini* | Hun7-YCFA-3 | 99.71 |
| ASV277 | NR_181111.1 | 97.974 | *Stenotrophomonas nematodicola* | Hun7-BLB-4 | 97.36 |
| ASV280 | NR_117542.1 | 97.295 | *Caloramator mitchellensis* | Sd2-YLS-3 | 97.44 |
| ASV281 | NR_116828.1 | 96.624 | *Paenibacillus prosopidis* | Hn1-NB-22 | 97.91 |
| ASV284 | NR_178248.1 | 98.619 | *Companilactobacillus formosensis* | Hn2-LBS-4 | 99.72 |
| ASV289 | NR_114742.1 | 97.841 | *Enterococcus faecium* | Nm2-YLS-1 | 100 |
| ASV292 | NR_181100.1 | 96.89 | *Companilactobacillus pabuli* | Hn2-LBS-6 | 99.71 |
| ASV293 | NR_109462.1 | 98.183 | *Paenibacillus typhae* | Hb1-YLS-9 | 99.79 |
| ASV299 | NR_145559.1 | 97.502 | *Paenibacillus populi* | Hn1-NB-21 | 99.06 |
| ASV301 | NR_025489.1 | 98.212 | *Paenibacillus agarexedens* | Hb2-TSA-6 | 99.85 |
| ASV305 | NR_134112.1 | 98.177 | *Paenibacillus dongdonensis* | Hn1-YCFA-10 | 99.37 |
| ASV306 | NR_113904.1 | 97.758 | *Enterococcus faecium* | Nm2-M17-5 | 99.93 |
| ASV307 | NR_116788.1 | 98.566 | *Paenibacillus tianmuensis* | Hn1-YLS-11 | 99.86 |
| ASV308 | NR_159230.1 | 96.393 | *Companilactobacillus musae* | Hb3-MRS-6 | 99.24 |
| ASV309 | NR_149778.1 | 98.558 | *Paenibacillus cucumis* | Hn2-LB-28 | 99.93 |
| ASV313 | NR_025490.1 | 96.499 | *Paenibacillus agaridevorans* | Hn1-NB-21 | 99.06 |
| ASV321 | NR_026490.1 | 96.322 | *Clostridium sartagoforme* | Hb1-LB-23 | 100 |
| ASV323 | NR_041381.1 | 97.222 | *Paenibacillus massiliensis* | Hn1-M17-14 | 99.65 |
| ASV325 | NR_113777.1 | 95.71 | *Neobacillus niacini* | Sd1-NB-21 | 99.57 |
| ASV326 | NR_178542.1 | 97.007 | *Paenibacillus ihuae* | Hn1-YCFA-2 | 99.79 |
| ASV329 | NR_025490.1 | 98.435 | *Paenibacillus agaridevorans* | Hn1-NB-21 | 97.98 |
| ASV330 | NR_181100.1 | 94.87 | *Companilactobacillus pabuli* | Hb3-MRS-6 | 100 |
| ASV336 | NR_025085.1 | 96.173 | *Paenibacillus granivorans* | Hn2-BLB-24 | 99.64 |
| ASV342 | NR_025085.1 | 98.512 | *Paenibacillus granivorans* | Hn2-BLB-24 | 99.49 |
| ASV345 | NR_025880.1 | 98.507 | *Lacticaseibacillus paracasei* | Sx3-YLS-3 | 99.86 |
| ASV348 | NR_025490.1 | 94.872 | *Paenibacillus agaridevorans* | Hn1-NB-21 | 99.42 |
| ASV350 | NR_114625.1 | 96.513 | *Paenibacillus turicensis* | Hn1-RCM-9 | 99.93 |
| ASV351 | NR_114625.1 | 94.437 | *Paenibacillus turicensis* | Hn2-M17-8 | 99.93 |
| ASV352 | NR_181100.1 | 98.887 | *Companilactobacillus pabuli* | Hn2-LBS-4 | 99.72 |
| ASV356 | NR_181873.1 | 97.404 | *Pseudoneobacillus rhizosphaerae* | Nm3-NB-16 | 98.32 |
| ASV361 | NR_117034.1 | 98.711 | *Paenibacillus anaericanus* | Hn1-RCM-9 | 98.41 |
| ASV367 | NR_134112.1 | 96.957 | *Paenibacillus dongdonensis* | Hn1-YLS-10 | 99.86 |
| ASV370 | NR_104277.1 | 97.374 | *Paenibacillus daejeonensis* | Sd1-YCFA-29 | 99.86 |
| ASV371 | NR_028865.1 | 98.575 | *Solibacillus silvestris* | Sd1-YLS-18 | 99.79 |
| ASV372 | NR_147397.1 | 98.722 | *Bacillus marasmi* | Hun7-YCFA-2 | 98.22 |
| ASV373 | NR_025490.1 | 95.442 | *Paenibacillus agaridevorans* | Hn1-NB-21 | 99.78 |
| ASV374 | NR_126179.1 | 96.598 | *Clostridium swellfunianum* | Nm3-TSA-21 | 97.3 |
| ASV383 | NR_180786.1 | 96.774 | *Alkalihalobacillus miscanthi* | Hb2-BLB-46 | 99.22 |
| ASV387 | NR_114398.1 | 97.222 | *Companilactobacillus farciminis* | Hn2-LBS-4 | 99.86 |
| ASV390 | NR_114094.1 | 98.566 | *Neobacillus novalis* | Hle2-NB-16 | 100 |
| ASV395 | NR_109462.1 | 98.51 | *Paenibacillus typhae* | Hn2-NB-17 | 98.94 |
| ASV401 | NR_104749.1 | 96.429 | *Mesobacillus subterraneus* | Sd1-NB-30 | 99.57 |
| ASV402 | NR_178542.1 | 96.867 | *Paenibacillus ihuae* | Hn1-YLS-11 | 99.51 |
| ASV403 | NR_043210.1 | 97.265 | *Litchfieldia alkalitelluris* | Sd2-BLB-28 | 100 |
| ASV404 | NR_171531.1 | 97.332 | *Siminovitchia acidinfaciens* | Sd1-TSA-10 | 99.93 |
| ASV406 | NR_043527.1 | 97.24 | *Sporosarcina soli* | Sd2-BLB-19 | 98.51 |
| ASV409 | NR_029109.1 | 97.665 | *Paenibacillus xylanilyticus* | Hb2-M17-6 | 99.69 |
| ASV411 | NR_136805.1 | 98.861 | *Marinithermofilum abyssi* | Nm3-RCM-13 | 97.67 |
| ASV415 | NR_113325.1 | 96.799 | *Clostridium tertium* | Hun4-DRCA-8 | 99.2 |
| ASV417 | NR_112628.1 | 98.697 | *Lysinibacillus fusiformis* | Hn2-RCM-7 | 99.07 |
| ASV418 | NR_043589.1 | 96.455 | *Peptostreptococcus stomatis* | Hb2-YLS-1 | 99.32 |
| ASV421 | NR_114920.1 | 97.619 | *Lysinibacillus macroides* | Hn2-YLS-13 | 100 |
| ASV422 | NR_178823.1 | 97.619 | *Clostridium thermarum* | Hle3-YCFA-12 | 97.37 |
| ASV425 | NR_042744.1 | 98.654 | *Virgibacillus kekensis* | Sx2-EMB-15 | 100 |
| ASV452 | NR_041293.1 | 98.846 | *Lentilactobacillus buchneri* | Sx2-DRCA-12 | 100 |
| ASV453 | NR_041293.1 | 96.449 | *Lentilactobacillus buchneri* | Sx2-DRCA-12 | 100 |
| ASV454 | NR_115116.1 | 96.393 | *Lentilactobacillus parabuchneri* | Sx2-DRCA-12 | 100 |
| ASV455 | NR_041293.1 | 98.788 | *Lentilactobacillus buchneri* | Sx2-DRCA-12 | 100 |
| ASV456 | NR_041830.1 | 98.306 | *Leuconostoc fallax* | Hle2-NB-16 | 100 |
| ASV473 | NR_117987.1 | 98.456 | *Lacticaseibacillus paracasei* | Nm3-M17-5 | 99.5 |
| ASV475 | NR_025880.1 | 97.668 | *Lacticaseibacillus paracasei* | Nm3-LB-6 | 97.37 |
| ASV501 | NR_180275.1 | 96.761 | *Companilactobacillus zhongbaensis* | Nm3-LB-6 | 100 |
| ASV565 | NR_042230.1 | 97.523 | *Lentilactobacillus kefiri* | Hle1-YLS-11 | 98.34 |
| ASV567 | NR_145916.1 | 98.478 | *Nocardioides glacieisoli* | Sx3-YLS-9 | 100 |
| ASV587 | NR_144696.1 | 98.885 | *Clostridium saudiense* | Hun7-LB-13 | 97.44 |
| ASV618 | NR_121710.1 | 97.773 | *Clostridium saccharobutylicum* | Nm3-TSA-4 | 97.62 |
| ASV628 | NR_115947.1 | 98.675 | *Paenibacillus donghaensis* | Sx3-EMB-20 | 97.33 |
| ASV642 | NR_149175.1 | 97.813 | *Bacillus mesophilus* | Sd1-NB-38 | 97.22 |
| ASV647 | NR_122090.1 | 98.189 | *Clostridium chromiireducens* | Sx3-YLS-9 | 100 |
| ASV649 | NR_042533.1 | 98.658 | *Companilactobacillus crustorum* | Hb3-MRS-6 | 98.79 |
| ASV651 | NR_026105.1 | 98.599 | *Clostridium puniceum* | Nm3-LB-18 | 97.62 |
| ASV665 | NR_144696.1 | 96.585 | *Clostridium saudiense* | Sd1-DRCA-21 | 100 |
| ASV669 | NR_025579.1 | 97.917 | *Levilactobacillus spicheri* | Tj-MRS-6 | 98.58 |
| ASV670 | NR_179391.1 | 98.886 | *Clostridium jeddahitimonense* | Hle1-TSA-6 | 97.44 |
| ASV677 | NR_158107.1 | 97.335 | *Niallia endozanthoxylica* | Nm2-M17-12 | 98.02 |
| ASV686 | NR_112632.1 | 97.147 | *Niallia circulans* | Sd1-DRCA-19 | 97.32 |
| ASV687 | NR_024817.1 | 98.577 | *Peribacillus asahii* | Hun7-TSA-6 | 97.43 |
| ASV692 | NR_148622.1 | 98.803 | *Paenibacillus etheri* | Sx3-EMB-20 | 99.21 |
| ASV701 | NR_180455.1 | 98.848 | *Rhodobacter xinxiangensis* | Nm2-RCM-16 | 97.3 |
| ASV713 | NR_112694.1 | 97.914 | *Ligilactobacillus pobuzihii* | Hun7-M17-4 | 97.8 |
| ASV753 | NR_043015.1 | 98.302 | *Metabacillus litoralis* | Sx1-BLB-17 | 99.08 |
| ASV756 | NR_151979.1 | 97.693 | *Niallia oryzisoli* | Sd1-DRCA-21 | 98.23 |
| ASV759 | NR_115947.1 | 98.605 | *Paenibacillus donghaensis* | Sx3-EMB-20 | 97.47 |
| ASV766 | NR_112728.1 | 98.576 | *Paenibacillus amylolyticus* | Hun4-BLB-11 | 99.72 |
| ASV778 | NR_025085.1 | 97.356 | *Paenibacillus granivorans* | Hn2-BLB-24 | 99.57 |
| ASV779 | NR_036850.1 | 98.573 | *Caryophanon latum* | Sd1-YLS-18 | 97.15 |
| ASV787 | NR_025045.1 | 98.523 | *Companilactobacillus kimchii* | Hb3-PYG-5 | 98.59 |
| ASV794 | NR_117690.2 | 93.144 | *Oxobacter pfennigii* | Sd1-NB-30 | 97.37 |
| ASV813 | NR_178486.1 | 98.346 | *Paenibacillus piscarius* | Hle3-LB-31 | 98.05 |
| ASV832 | NR_104768.1 | 97.84 | *Clostridium carboxidivorans* | Sx3-YLS-9 | 100 |
| ASV843 | NR_134019.1 | 96.752 | *Diaminobutyricibacter tongyongensis* | Sx3-YLS-9 | 97.44 |
| ASV847 | NR_145559.1 | 96.857 | *Paenibacillus populi* | Hn1-NB-21 | 97.26 |
| ASV853 | NR_147741.1 | 97.634 | *Paenibacillus terreus* | Sd2-BLB-24 | 97.11 |
| ASV855 | NR_108831.1 | 98.57 | *Peribacillus endoradicis* | Hle1-TSA-1 | 97.14 |
| ASV857 | NR_036950.1 | 98.769 | *Clostridium saccharoperbutylacetonicum* | Hle3-NB-14 | 97.62 |
| ASV870 | NR_126179.1 | 94.122 | *Clostridium swellfunianum* | Sd1-LB-15 | 97.73 |
| ASV886 | NR_025489.1 | 98.707 | *Paenibacillus agarexedens* | Hn2-TSA-30 | 99.86 |
| ASV888 | NR_114398.1 | 97.317 | *Companilactobacillus farciminis* | Hn2-MRS-1 | 97.57 |
| ASV889 | NR_148613.1 | 93.885 | *Paenibacillus cavernae* | Hn1-DRCA-1 | 99.43 |
| ASV899 | NR_112694.1 | 97.981 | *Ligilactobacillus pobuzihii* | Hun7-M17-4 | 97.87 |
| ASV908 | NR_112694.1 | 97.914 | *Ligilactobacillus pobuzihii* | Hun7-M17-4 | 97.8 |
| ASV911 | NR_112694.1 | 97.914 | *Ligilactobacillus pobuzihii* | Hun7-M17-4 | 97.87 |
| ASV914 | NR_180274.1 | 94.081 | *Companilactobacillus mishanensis* | Nm3-LB-6 | 100 |
| ASV920 | NR_042058.1 | 98.402 | *Pediococcus pentosaceus* | Hle2-MRS-14 | 98.59 |
| ASV930 | NR_024931.1 | 98.652 | *Bacillus spizizenii* | Hb1-TSA-5 | 98.78 |
| ASV934 | NR_116873.1 | 98.777 | *Priestia megaterium* | Sd2-BLB-1 | 99.04 |
| ASV936 | NR_112636.1 | 97.493 | *Priestia megaterium* | Nm2-EMB-2 | 99.44 |
| ASV941 | NR_024931.1 | 97.803 | *Bacillus spizizenii* | Hun4-NB-8 | 97.84 |
| ASV954 | NR_134073.1 | 98.043 | *Lysinibacillus halotolerans* | Nm2-LB-19 | 98.34 |
| ASV982 | NR_042195.1 | 98.259 | *Loigolactobacillus rennini* | Nm3-LB-6 | 100 |
| ASV984 | NR_042195.1 | 98.189 | *Loigolactobacillus rennini* | Nm3-LB-1 | 100 |
| ASV1016 | NR_025085.1 | 97.356 | *Paenibacillus granivorans* | Hn2-BLB-24 | 100 |
| ASV1032 | NR_115175.1 | 98.849 | *Paenibacillus massiliensis* | Hn1-NB-14 | 99.57 |
| ASV1039 | NR_025085.1 | 97.22 | *Paenibacillus granivorans* | Hn2-BLB-24 | 99.71 |
| ASV1049 | NR_158060.1 | 97.381 | *Secundilactobacillus pentosiphilus* | Sx1-YLS-15 | 97.27 |
| ASV1085 | NR_112693.1 | 98.319 | *Ligilactobacillus acidipiscis* | Hun7-LBS-1 | 98.79 |
| ASV1108 | NR_180275.1 | 98.362 | *Companilactobacillus zhongbaensis* | Hb3-MRS-6 | 98.17 |
| ASV1119 | NR_117973.1 | 98.725 | *Companilactobacillus futsaii* | Hb3-MRS-6 | 99.01 |
| ASV1125 | NR_041293.1 | 97.996 | *Lentilactobacillus buchneri* | Sx2-DRCA-12 | 100 |
| ASV1128 | NR_042243.1 | 97.648 | *Levilactobacillus hammesii* | Nm2-DRCA-3 | 97.46 |
| ASV1135 | NR_115271.1 | 97.763 | *Lentilactobacillus kefiri* | Hle1-YLS-11 | 98.11 |
| ASV1158 | NR_112632.1 | 97.147 | *Niallia circulans* | Sd1-DRCA-19 | 97.32 |
| ASV1172 | NR_029239.3 | 98.043 | *Clostridium chartatabidum* | Sx3-YLS-9 | 100 |
| ASV1175 | NR_112694.1 | 97.914 | *Ligilactobacillus pobuzihii* | Hun7-M17-4 | 97.8 |
| ASV1177 | NR_114978.1 | 97.667 | *Paucilactobacillus vaccinostercus* | Hb3-MRS-1 | 97.48 |
| ASV1180 | NR_029133.1 | 97.921 | *Lactiplantibacillus pentosus* | Sx3-TSA-8 | 98.2 |
| ASV1181 | NR_112694.1 | 97.847 | *Ligilactobacillus pobuzihii* | Hun7-M17-4 | 97.72 |
| ASV1189 | NR_041293.1 | 98.199 | *Lentilactobacillus buchneri* | Sx2-DRCA-12 | 100 |
| ASV1191 | NR_041293.1 | 98.131 | *Lentilactobacillus buchneri* | Nm3-LB-6 | 100 |
| ASV1192 | NR_042230.1 | 97.311 | *Lentilactobacillus kefiri* | Hle1-YLS-11 | 97.73 |
| ASV1195 | NR_042533.1 | 98.591 | *Companilactobacillus crustorum* | Hb3-MRS-6 | 98.72 |
| ASV1203 | NR_113338.1 | 98.859 | *Lactiplantibacillus plantarum* | Hb1-BLB-14 | 99.14 |
| ASV1204 | NR_113338.1 | 97.116 | *Lactiplantibacillus plantarum* | Hb1-BLB-14 | 97.28 |
| ASV1206 | NR_115308.1 | 97.368 | *Liquorilactobacillus uvarum* | Hb1-LBS-1 | 97.3 |
| ASV1210 | NR_042456.1 | 97.246 | *Levilactobacillus parabrevis* | Hb3-MRS-1 | 97.64 |
| ASV1214 | NR_113338.1 | 98.524 | *Lactiplantibacillus plantarum* | Hb3-SB-9 | 98.74 |
| ASV1216 | NR_114251.1 | 98.254 | *Levilactobacillus senmaizukei* | Sx3-RCM-7 | 98.08 |
| ASV1217 | NR_113338.1 | 98.054 | *Lactiplantibacillus plantarum* | Hb1-BLB-14 | 98.28 |
| ASV1221 | NR_042230.1 | 97.491 | *Lentilactobacillus kefiri* | Tj-MRS-11 | 97.77 |
| ASV1230 | NR_180275.1 | 94.789 | *Companilactobacillus zhongbaensis* | Nm3-LB-6 | 100 |
| ASV1250 | NR_042243.1 | 97.649 | *Levilactobacillus hammesii* | Nm2-DRCA-3 | 97.39 |
| ASV1257 | NR_025882.1 | 98.712 | *Paenibacillus amylolyticus* | Hun4-BLB-11 | 99.93 |
| ASV1265 | NR_112693.1 | 98.453 | *Ligilactobacillus acidipiscis* | Sd1-M17-46 | 98.76 |
| ASV1281 | NR_181100.1 | 98.858 | *Companilactobacillus pabuli* | Hb3-MRS-6 | 100 |
| ASV1293 | NR_027553.1 | 95.054 | *Bdellovibrio bacteriovorus* | Hle2-NB-16 | 97.3 |
| ASV1316 | NR_117073.1 | 97.462 | *Lactobacillus acetotolerans* | Nm3-LB-6 | 100 |
| ASV1364 | NR_029133.1 | 98.322 | *Lactiplantibacillus pentosus* | Sx3-M17-17 | 98.86 |
| ASV1365 | NR_145899.1 | 97.806 | *Lactiplantibacillus herbarum* | Hb3-MRS-1 | 98.34 |
| ASV1366 | NR_114398.1 | 97.651 | *Companilactobacillus farciminis* | Hn2-LBS-7 | 97.99 |
| ASV1367 | NR_115308.1 | 97.5 | *Liquorilactobacillus uvarum* | Nm3-LB-6 | 100 |
| ASV1368 | NR_181134.1 | 98.59 | *Levilactobacillus tujiorum* | Sx3-RCM-7 | 98.01 |
| ASV1371 | NR_145899.1 | 97.233 | *Lactiplantibacillus herbarum* | Nm3-LB-6 | 100 |
| ASV1385 | NR_112693.1 | 96.714 | *Ligilactobacillus acidipiscis* | Sd1-LBS-5 | 97.35 |
| ASV1418 | NR_025490.1 | 98.307 | *Paenibacillus agaridevorans* | Hn1-NB-21 | 99.49 |
| ASV1425 | NR_181131.1 | 97.443 | *Paenibacillus agri* | Hle3-LB-31 | 97.68 |
| ASV1428 | NR_025490.1 | 98.111 | *Paenibacillus agaridevorans* | Hn1-NB-21 | 99.5 |
| ASV1436 | NR_029239.3 | 97.973 | *Clostridium chartatabidum* | Sx3-YLS-9 | 100 |
| ASV1441 | NR_025489.1 | 98.844 | *Paenibacillus agarexedens* | Hn2-TSA-30 | 99.86 |
| ASV1444 | NR_025085.1 | 97.288 | *Paenibacillus granivorans* | Hn2-BLB-24 | 99.64 |
| ASV1450 | NR_025490.1 | 98.177 | *Paenibacillus agaridevorans* | Hn1-NB-21 | 99.93 |
| ASV1456 | NR_025085.1 | 97.153 | *Paenibacillus granivorans* | Hn2-BLB-24 | 99.93 |
| ASV1465 | NR_134112.1 | 98.832 | *Paenibacillus dongdonensis* | Hn1-YCFA-10 | 99.08 |
| ASV1471 | NR_025490.1 | 97.97 | *Paenibacillus agaridevorans* | Hn1-NB-21 | 98.92 |
| ASV1472 | NR_134112.1 | 98.832 | *Paenibacillus dongdonensis* | Hn1-YLS-31 | 99.16 |
| ASV1475 | NR_145559.1 | 98.077 | *Paenibacillus populi* | Hn1-NB-21 | 99.28 |
| ASV1476 | NR_025490.1 | 97.495 | *Paenibacillus agaridevorans* | Hn1-NB-21 | 97.98 |
| ASV1477 | NR_025085.1 | 97.488 | *Paenibacillus granivorans* | Hn2-BLB-24 | 99.56 |
| ASV1480 | NR_043015.1 | 98.845 | *Metabacillus litoralis* | Hb2-BLB-13 | 99.93 |
| ASV1481 | NR_180481.1 | 97.943 | *Bacillus salipaludis* | Hun7-YCFA-2 | 98.3 |
| ASV1484 | NR_178542.1 | 98.639 | *Paenibacillus ihuae* | Hn1-YCFA-2 | 99.93 |
| ASV1494 | NR_145559.1 | 98.695 | *Paenibacillus populi* | Hn1-NB-21 | 99.86 |
| ASV1495 | NR_181636.1 | 98.539 | *Paenibacillus tianjinensis* | Hn2-NB-17 | 98.42 |
| ASV1498 | NR_181636.1 | 98.466 | *Paenibacillus tianjinensis* | Sx3-EMB-20 | 98.27 |
| ASV1500 | NR_135705.1 | 98.566 | *Paenibacillus endophyticus* | Hn1-BLB-37 | 97.61 |
| ASV1502 | NR_025490.1 | 97.766 | *Paenibacillus agaridevorans* | Hn1-NB-21 | 98.92 |
| ASV1505 | NR_145559.1 | 98.077 | *Paenibacillus populi* | Hn1-NB-21 | 99.13 |
| ASV1507 | NR_181636.1 | 98.393 | *Paenibacillus tianjinensis* | Hn2-NB-17 | 98.35 |
| ASV1508 | NR_181131.1 | 97.443 | *Paenibacillus agri* | Hle3-LB-31 | 97.47 |
| ASV1510 | NR_181636.1 | 98.612 | *Paenibacillus tianjinensis* | Hn1-TSA-34 | 98.47 |
| ASV1512 | NR_181636.1 | 98.462 | *Paenibacillus tianjinensis* | Hn1-TSA-34 | 98.54 |
| ASV1514 | NR_025490.1 | 98.042 | *Paenibacillus agaridevorans* | Hn1-NB-21 | 99.64 |
| ASV1516 | NR_181636.1 | 98.533 | *Paenibacillus tianjinensis* | Hn1-TSA-34 | 98.61 |
| ASV1518 | NR_116955.1 | 97.551 | *Sporosarcina contaminans* | Sx1-LB-8 | 97.61 |
| ASV1521 | NR_024817.1 | 98.847 | *Peribacillus asahii* | Hun7-TSA-6 | 97.29 |
| ASV1527 | NR_025490.1 | 97.232 | *Paenibacillus agaridevorans* | Hn1-NB-21 | 98.78 |
| ASV1529 | NR_126179.1 | 98.248 | *Clostridium swellfunianum* | Nm3-TSA-21 | 97.3 |
| ASV1536 | NR_178753.1 | 98.556 | *Paenibacillus puernese* | Hb2-M17-6 | 99.39 |
| ASV1537 | NR_041377.1 | 98.102 | *Neobacillus pocheonensis* | Nm3-NB-16 | 97.91 |
| ASV1538 | NR_024817.1 | 98.169 | *Peribacillus asahii* | Hun4-DRCA-18 | 97.62 |
| ASV1541 | NR_114625.1 | 96.491 | *Paenibacillus turicensis* | Hn2-M17-8 | 99.71 |
| ASV1543 | NR_144724.1 | 94.386 | *Desnuesiella massiliensis* | Nm2-RCM-10 | 100 |
| ASV1544 | NR_133702.1 | 98.138 | *Lederbergia panacisoli* | Sd2-BLB-20 | 97.75 |
| ASV1545 | NR_148613.1 | 93.746 | *Paenibacillus cavernae* | Hn1-DRCA-1 | 99.43 |
| ASV1546 | NR_148613.1 | 94.019 | *Paenibacillus cavernae* | Hn1-DRCA-1 | 99.57 |
| ASV1548 | NR_109462.1 | 98.435 | *Paenibacillus typhae* | Hn1-YLS-11 | 98.8 |
| ASV1549 | NR_025490.1 | 97.569 | *Paenibacillus agaridevorans* | Hn1-NB-21 | 99.14 |
| ASV1552 | NR_181636.1 | 98.536 | *Paenibacillus tianjinensis* | Hn2-NB-17 | 98.56 |
| ASV1558 | NR_181636.1 | 98.32 | *Paenibacillus tianjinensis* | Hn1-TSA-34 | 98.39 |
| ASV1559 | NR_126271.1 | 98.179 | *Paenibacillus pinisoli* | Hn2-M17-6 | 99.64 |
| ASV1560 | NR_126271.1 | 98.381 | *Paenibacillus pinisoli* | Hn2-M17-6 | 99.22 |
| ASV1563 | NR_043527.1 | 98.033 | *Sporosarcina soli* | Hb1-BLB-25 | 97.89 |
| ASV1564 | NR_025489.1 | 98.776 | *Paenibacillus agarexedens* | Hn2-TSA-30 | 99.86 |
| ASV1566 | NR_126179.1 | 98.108 | *Clostridium swellfunianum* | Nm3-TSA-21 | 97.14 |
| ASV1569 | NR_112632.1 | 98.031 | *Niallia circulans* | Hle2-LB-5 | 99.36 |
| ASV1571 | NR_026149.1 | 98.541 | *Clostridium quinii* | Nm2-LB-18 | 100 |
| ASV1573 | NR_181636.1 | 98.32 | *Paenibacillus tianjinensis* | Hn1-TSA-34 | 98.4 |
| ASV1574 | NR_024817.1 | 98.512 | *Peribacillus asahii* | Hun4-DRCA-18 | 97.11 |
| ASV1576 | NR_109725.1 | 95.122 | *Melghirimyces thermohalophilus* | Nm3-RCM-13 | 97.67 |
| ASV1594 | NR_145559.1 | 98.42 | *Paenibacillus populi* | Hn1-NB-21 | 99.42 |
| ASV1596 | NR_158107.1 | 97.335 | *Niallia endozanthoxylica* | Hb1-BLB-20 | 98.09 |
| ASV1610 | NR_117690.2 | 94.66 | *Oxobacter pfennigii* | Sd2-DRCA-4 | 100 |
| ASV1620 | NR_147397.1 | 98.573 | *Bacillus marasmi* | Nm2-YCFA-4 | 98.23 |
| ASV1622 | NR_180481.1 | 98.723 | *Bacillus salipaludis* | Hun7-YCFA-3 | 99.35 |
| ASV1627 | NR_025490.1 | 97.436 | *Paenibacillus agaridevorans* | Hn1-NB-21 | 98.78 |
| ASV1631 | NR_115691.1 | 92.494 | *Gracilibacter thermotolerans* | Nm3-RCM-16 | 97.44 |
| ASV1640 | NR_178823.1 | 97.402 | *Clostridium thermarum* | Nm2-MRS-22 | 100 |
| ASV1641 | NR_181873.1 | 98.438 | *Pseudoneobacillus rhizosphaerae* | Nm3-NB-16 | 97.97 |
| ASV1642 | NR_102516.1 | 98.676 | *Clostridium saccharoperbutylacetonicum* | Hle3-NB-14 | 97.62 |
| ASV1659 | NR_144724.1 | 95.081 | *Desnuesiella massiliensis* | Hb2-NB-14 | 97.67 |
| ASV1675 | NR_180481.1 | 97.589 | *Bacillus salipaludis* | Hun7-RCM-12 | 97.95 |
| ASV1676 | NR_025490.1 | 97.502 | *Paenibacillus agaridevorans* | Hn1-NB-21 | 99.06 |
| ASV1691 | NR_117012.1 | 97.228 | *Paenibacillus uliginis* | Sx3-M17-1 | 97.05 |
| ASV1693 | NR_115578.1 | 98.654 | *Bacillus benzoevorans* | Sd1-DRCA-21 | 97.57 |
| ASV1700 | NR_178823.1 | 97.051 | *Clostridium thermarum* | Hle3-YCFA-12 | 97.37 |
| ASV1705 | NR_144724.1 | 94.67 | *Desnuesiella massiliensis* | Nm3-TSA-9 | 100 |
| ASV1707 | NR_180455.1 | 98.704 | *Rhodobacter xinxiangensis* | Nm2-RCM-16 | 97.3 |
| ASV1712 | NR_117690.2 | 93.333 | *Oxobacter pfennigii* | Hun4-DRCA-15 | 97.5 |
| ASV1718 | NR_029263.1 | 98.052 | *Clostridium intestinale* | Sx3-YLS-9 | 100 |
| ASV1726 | NR_117285.1 | 98.492 | *Cytobacillus oceanisediminis* | Hb2-LB-15 | 98.43 |
| ASV1733 | NR_026336.1 | 96.451 | *Clostridium vincentii* | Hb2-LB-11 | 100 |
| ASV1762 | NR_180120.1 | 98.051 | *Paracoccus endophyticus* | Nm2-RCM-16 | 100 |
| ASV1768 | NR_158107.1 | 97.546 | *Niallia endozanthoxylica* | Hb1-BLB-20 | 98.01 |
| ASV1769 | NR_113577.1 | 97.76 | *Paenibacillus alvei* | Nm2-YLS-11 | 97.11 |
| ASV1782 | NR_041293.1 | 97.333 | *Lentilactobacillus buchneri* | Sx2-DRCA-12 | 100 |
| ASV1783 | NR_109000.1 | 97.17 | *Lactiplantibacillus xiangfangensis* | Hb3-DRCA-6 | 97.41 |
| ASV1785 | NR_113338.1 | 98.321 | *Lactiplantibacillus plantarum* | Hb1-BLB-14 | 98.71 |
| ASV1786 | NR_109452.1 | 97.589 | *Levilactobacillus yonginensis* | Sx3-RCM-7 | 98.16 |
| ASV1787 | NR_042230.1 | 97.276 | *Lentilactobacillus kefiri* | Hle1-YLS-11 | 97.8 |
| ASV1788 | NR_113906.1 | 98.179 | *Enterococcus mundtii* | Sx1-MRS-9 | 97.83 |
| ASV1794 | NR_113338.1 | 97.122 | *Lactiplantibacillus plantarum* | Hb1-BLB-14 | 97.64 |
| ASV1796 | NR_117987.1 | 98.607 | *Lacticaseibacillus paracasei* | Nm3-M17-5 | 99.07 |
| ASV1797 | NR_117813.1 | 98.765 | *Lactiplantibacillus plantarum* | Hb1-BLB-5 | 99.13 |
| ASV1798 | NR_113906.1 | 98.719 | *Enterococcus mundtii* | Sx3-YCFA-6 | 100 |
| ASV1799 | NR_113906.1 | 96.97 | *Enterococcus mundtii* | Sx3-YCFA-6 | 100 |
| ASV1803 | NR_113332.1 | 96.722 | *Lacticaseibacillus rhamnosus* | Hun4-MRS-11 | 97.01 |
| ASV1806 | NR_029133.1 | 98.59 | *Lactiplantibacillus pentosus* | Hb3-SB-9 | 99.05 |
| ASV1807 | NR_113338.1 | 97.451 | *Lactiplantibacillus plantarum* | Hb1-BLB-14 | 97.56 |
| ASV1808 | NR_109452.1 | 97.376 | *Levilactobacillus yonginensis* | Hb1-TSA-13 | 97.79 |
| ASV1809 | NR_116238.1 | 98.672 | *Levilactobacillus brevis* | Sx3-RCM-7 | 99.34 |
| ASV1832 | NR_178542.1 | 98.503 | *Paenibacillus ihuae* | Hn1-YCFA-2 | 99.93 |
| ASV1833 | NR_025489.1 | 98.844 | *Paenibacillus agarexedens* | Hn2-TSA-30 | 100 |
| ASV1834 | NR_044524.1 | 98.847 | *Paenibacillus xylanexedens* | Hle3-EMB-8 | 99.58 |
| ASV1837 | NR_165702.1 | 98.852 | *Paenibacillus seodonensis* | Sd2-EMB-19 | 99.71 |
| ASV1850 | NR_025882.1 | 98.78 | *Paenibacillus amylolyticus* | Hun4-TSA-19 | 99.93 |
| ASV1852 | NR_179131.1 | 98.833 | *Paenibacillus panacihumi* | Hb2-TSA-13 | 99.86 |
| ASV1854 | NR_025085.1 | 97.288 | *Paenibacillus granivorans* | Hn2-BLB-24 | 99.64 |
| ASV1855 | NR_112728.1 | 98.712 | *Paenibacillus amylolyticus* | Hun4-TSA-22 | 99.79 |
| ASV1859 | NR_163642.1 | 98.7 | *Paenibacillus mobilis* | Hun4-TSA-19 | 99.71 |
| ASV1862 | NR_025882.1 | 98.714 | *Paenibacillus amylolyticus* | Hle3-LB-10 | 99.44 |
| ASV1868 | NR_113330.1 | 97.917 | *Oceanobacillus indicireducens* | Hun7-NB-6 | 100 |
| ASV1871 | NR_025882.1 | 98.646 | *Paenibacillus amylolyticus* | Hle3-LB-10 | 99.51 |
| ASV1874 | NR_145559.1 | 98.695 | *Paenibacillus populi* | Hn1-NB-21 | 99.78 |
| ASV1877 | NR_044525.1 | 98.78 | *Paenibacillus tundrae* | Hle3-M17-10 | 99.65 |
| ASV1883 | NR_025882.1 | 98.578 | *Paenibacillus amylolyticus* | Hle3-LB-10 | 99.44 |
| ASV1884 | NR_113330.1 | 97.917 | *Oceanobacillus indicireducens* | Hun7-NB-6 | 100 |
| ASV1892 | NR_043698.1 | 98.813 | *Clostridium tagluense* | Sx1-LB-3 | 97.44 |
| ASV1897 | NR_025490.1 | 97.698 | *Paenibacillus agaridevorans* | Hn1-NB-21 | 98.13 |
| ASV1901 | NR_112844.1 | 97.493 | *Sporosarcina luteola* | Sd1-RCM-10 | 97.51 |
| ASV1903 | NR_025085.1 | 97.42 | *Paenibacillus granivorans* | Hn2-BLB-24 | 99.49 |
| ASV1904 | NR_025882.1 | 98.578 | *Paenibacillus amylolyticus* | Hle3-YCFA-10 | 99.28 |
| ASV1912 | NR_151979.1 | 97.829 | *Niallia oryzisoli* | Hn1-YLS-4 | 98.43 |
| ASV1913 | NR_151979.1 | 97.626 | *Niallia oryzisoli* | Hb1-BLB-20 | 98.02 |
| ASV1918 | NR_025882.1 | 98.847 | *Paenibacillus amylolyticus* | Hun4-TSA-19 | 99.93 |
| ASV1919 | NR_181873.1 | 98.71 | *Pseudoneobacillus rhizosphaerae* | Nm3-NB-16 | 98.11 |
| ASV1922 | NR_025490.1 | 98.307 | *Paenibacillus agaridevorans* | Hn1-NB-21 | 99.49 |
| ASV1926 | NR_112632.1 | 97.215 | *Niallia circulans* | Sd1-DRCA-19 | 97.39 |
| ASV1929 | NR_043698.1 | 98.742 | *Clostridium tagluense* | Sx1-LB-3 | 97.44 |
| ASV1936 | NR_145559.1 | 97.601 | *Paenibacillus populi* | Hn1-NB-21 | 98.56 |
| ASV1937 | NR_145585.1 | 98.743 | *Peribacillus gossypii* | Sx3-NB-20 | 98.19 |
| ASV1938 | NR_149175.1 | 97.881 | *Bacillus mesophilus* | Sd1-NB-38 | 97.15 |
| ASV1941 | NR_025490.1 | 97.569 | *Paenibacillus agaridevorans* | Hn1-NB-21 | 99.14 |
| ASV1943 | NR_180378.1 | 97.831 | *Lysinibacillus antri* | Sd2-BLB-22 | 98.63 |
| ASV1946 | NR_025489.1 | 98.707 | *Paenibacillus agarexedens* | Hn2-TSA-30 | 99.71 |
| ASV1947 | NR_025490.1 | 97.571 | *Paenibacillus agaridevorans* | Hn1-NB-21 | 98.92 |
| ASV1948 | NR_025489.1 | 98.442 | *Paenibacillus agarexedens* | Hn2-TSA-30 | 99.28 |
| ASV1950 | NR_163642.1 | 98.702 | *Paenibacillus mobilis* | Hle3-LB-10 | 99.01 |
| ASV1956 | NR_114626.1 | 97.233 | *Paenibacillus turicensis* | Hn1-RCM-9 | 98.33 |
| ASV1960 | NR_145559.1 | 98.077 | *Paenibacillus populi* | Hn1-NB-21 | 98.92 |
| ASV1961 | NR_114625.1 | 97.23 | *Paenibacillus turicensis* | Hn1-RCM-9 | 99.56 |
| ASV1962 | NR_025489.1 | 97.97 | *Paenibacillus agarexedens* | Hb2-TSA-6 | 99.85 |
| ASV1965 | NR_043015.1 | 98.845 | *Metabacillus litoralis* | Hb2-BLB-13 | 99.79 |
| ASV1971 | NR_178542.1 | 98.571 | *Paenibacillus ihuae* | Hn1-YCFA-2 | 99.29 |
| ASV1979 | NR_133974.1 | 98.494 | *Peribacillus huizhouensis* | Sx1-NB-11 | 98.55 |
| ASV1980 | NR_151979.1 | 97.422 | *Niallia oryzisoli* | Hn1-YLS-4 | 98.05 |
| ASV1981 | NR_114625.1 | 96.556 | *Paenibacillus turicensis* | Hn2-M17-8 | 99.86 |
| ASV1985 | NR_025490.1 | 98.043 | *Paenibacillus agaridevorans* | Hn1-NB-21 | 99.42 |
| ASV1987 | NR_181753.1 | 96.606 | *Bacillus weihaiensis* | Sd2-MRS-4 | 97.79 |
| ASV1990 | NR_163642.1 | 98.769 | *Paenibacillus mobilis* | Hun4-TSA-19 | 99.64 |
| ASV1992 | NR_025489.1 | 98.707 | *Paenibacillus agarexedens* | Hn2-TSA-30 | 99.86 |
| ASV2000 | NR_025882.1 | 98.644 | *Paenibacillus amylolyticus* | Hun4-TSA-19 | 99.86 |
| ASV2012 | NR_135705.1 | 98.838 | *Paenibacillus endophyticus* | Hn1-NB-22 | 97.84 |
| ASV2013 | NR_025490.1 | 97.097 | *Paenibacillus agaridevorans* | Hn1-NB-21 | 98.42 |
| ASV2016 | NR_041533.1 | 98.858 | *Oceanobacillus caeni* | Hle2-NB-18 | 100 |
| ASV2019 | NR_044524.1 | 98.646 | *Paenibacillus xylanexedens* | Hle3-EMB-8 | 99.01 |
| ASV2021 | NR_041377.1 | 98.575 | *Neobacillus pocheonensis* | Hun7-YCFA-3 | 98.55 |
| ASV2024 | NR_145559.1 | 97.665 | *Paenibacillus populi* | Hn1-NB-21 | 98.85 |
| ASV2029 | NR_134005.1 | 95.083 | *Clostridium bornimense* | Nm2-EMB-26 | 97.44 |
| ASV2032 | NR_181636.1 | 98.559 | *Paenibacillus tianjinensis* | Hb1-YLS-9 | 98.87 |
| ASV2033 | NR_151979.1 | 97.695 | *Niallia oryzisoli* | Hn1-YLS-4 | 97.91 |
| ASV2036 | NR_113388.1 | 98.536 | *Clostridium beijerinckii* | Hun7-TSA-20 | 97.62 |
| ASV2037 | NR_158107.1 | 97.195 | *Niallia endozanthoxylica* | Hb1-BLB-20 | 97.87 |
| ASV2041 | NR_104504.1 | 92.681 | *Fervidicella metallireducens* | Hle1-RCM-7 | 97.5 |
| ASV2046 | NR_112632.1 | 97.153 | *Niallia circulans* | Sd1-DRCA-21 | 97.87 |
| ASV2048 | NR_145559.1 | 97.735 | *Paenibacillus populi* | Hn1-NB-21 | 98.77 |
| ASV2053 | NR_178823.1 | 97.051 | *Clostridium thermarum* | Hle3-NB-14 | 97.37 |
| ASV2060 | NR_109669.1 | 98.577 | *Ureibacillus chungkukjangi* | Nm2-TSA-13 | 98.5 |
| ASV2066 | NR_025170.1 | 98.784 | *Paenibacillus terrae* | Sx1-DRCA-18 | 98.2 |
| ASV2068 | NR_108870.1 | 91.349 | *Fonticella tunisiensis* | Nm3-NB-20 | 97.06 |
| ASV2073 | NR_102516.1 | 98.467 | *Clostridium saccharoperbutylacetonicum* | Hle3-NB-14 | 97.62 |
| ASV2074 | NR_117285.1 | 98.063 | *Cytobacillus oceanisediminis* | Nm2-M17-13 | 98.27 |
| ASV2076 | NR_146034.1 | 97.447 | *Cytobacillus depressus* | Hb1-BLB-20 | 98.15 |
| ASV2083 | NR_112632.1 | 97.352 | *Niallia circulans* | Hn1-YLS-4 | 98.2 |
| ASV2088 | NR_145585.1 | 97.556 | *Peribacillus gossypii* | Hun4-DRCA-18 | 97.68 |
| ASV2092 | NR_117690.2 | 93.616 | *Oxobacter pfennigii* | Hun7-M17-12 | 100 |
| ASV2125 | NR_025490.1 | 96.691 | *Paenibacillus agaridevorans* | Hn1-NB-21 | 97.84 |
| ASV2132 | NR_122090.1 | 98.468 | *Clostridium chromiireducens* | Sx3-YLS-9 | 100 |
| ASV2134 | NR_144724.1 | 95.429 | *Desnuesiella massiliensis* | Sd2-BLB-40 | 100 |
| ASV2142 | NR_104899.1 | 98.281 | *Hungatella xylanolytica* | Hb1-EMB-21 | 98.68 |
| ASV2148 | NR_036950.1 | 98.841 | *Clostridium saccharoperbutylacetonicum* | Hle3-NB-14 | 97.44 |
| ASV2164 | NR_145585.1 | 98.187 | *Peribacillus gossypii* | Hle2-NB-8 | 97.94 |
| ASV2177 | NR_117690.2 | 94.183 | *Oxobacter pfennigii* | Sx1-BLB-15 | 97.5 |
| ASV2178 | NR_144724.1 | 94.596 | *Desnuesiella massiliensis* | Nm2-RCM-10 | 100 |
| ASV2180 | NR_026100.1 | 98.206 | *Clostridium celerecrescens* | Hb1-LB-20 | 98.91 |
| ASV2181 | NR_145559.1 | 98.008 | *Paenibacillus populi* | Hn1-NB-21 | 98.2 |
| ASV2199 | NR_151979.1 | 97.354 | *Niallia oryzisoli* | Hun7-RCM-12 | 98.1 |
| ASV2201 | NR_144724.1 | 95.151 | *Desnuesiella massiliensis* | Sd2-BLB-40 | 100 |
| ASV2203 | NR_112632.1 | 97.622 | *Niallia circulans* | Hun7-RCM-12 | 98.68 |
| ASV2205 | NR_036950.1 | 98.406 | *Clostridium saccharoperbutylacetonicum* | Hle3-NB-14 | 97.62 |
| ASV2210 | NR_025490.1 | 97.901 | *Paenibacillus agaridevorans* | Hn1-NB-21 | 98.85 |
| ASV2213 | NR_178823.1 | 96.014 | *Clostridium thermarum* | Hle3-YCFA-12 | 97.44 |
| ASV2223 | NR_134006.1 | 95.399 | *Clostridium huakuii* | Hun7-TSA-20 | 100 |
| ASV2240 | NR_025490.1 | 97.63 | *Paenibacillus agaridevorans* | Hn1-NB-21 | 98.77 |
| ASV2242 | NR_165702.1 | 98.709 | *Paenibacillus seodonensis* | Hb2-TSA-13 | 99.43 |
| ASV2256 | NR_180378.1 | 98.171 | *Lysinibacillus antri* | Nm2-LB-19 | 98.56 |
| ASV2267 | NR_024695.1 | 98.641 | *Neobacillus niacini* | Hun7-YCFA-3 | 98.92 |
| ASV2286 | NR_117690.2 | 91.367 | *Oxobacter pfennigii* | Sx1-BLB-15 | 97.44 |
| ASV2301 | NR_158107.1 | 97.127 | *Niallia endozanthoxylica* | Hb1-BLB-20 | 97.79 |
| ASV2304 | NR_114879.1 | 98.544 | *Clostridium bowmanii* | Nm3-BLB-21 | 97.5 |
| ASV2323 | NR_133974.1 | 98.016 | *Peribacillus huizhouensis* | Sx3-NB-17 | 97.56 |
| ASV2332 | NR_117689.2 | 92.266 | *Oxobacter pfennigii* | Sx3-BLB-5 | 97.5 |
| ASV2350 | NR_109459.1 | 94.737 | *Pseudalkalibacillus berkeleyi* | Hle1-RCM-11 | 97.37 |
| ASV2351 | NR_178823.1 | 95.649 | *Clostridium thermarum* | Sx3-RCM-15 | 100 |
| ASV2385 | NR_029133.1 | 97.181 | *Lactiplantibacillus pentosus* | Hb1-BLB-5 | 97.6 |
| ASV2387 | NR_145899.1 | 97.806 | *Lactiplantibacillus herbarum* | Hb3-MRS-1 | 98.34 |
| ASV2392 | NR_029133.1 | 98.791 | *Lactiplantibacillus pentosus* | Hb1-BLB-5 | 99.34 |
| ASV2396 | NR_181134.1 | 98.522 | *Levilactobacillus tujiorum* | Sx3-RCM-7 | 98.08 |
| ASV2399 | NR_041293.1 | 97.8 | *Lentilactobacillus buchneri* | Sx2-DRCA-12 | 100 |
| ASV2416 | NR_025085.1 | 97.22 | *Paenibacillus granivorans* | Hn2-BLB-24 | 99.86 |
| ASV2422 | NR_114625.1 | 96.494 | *Paenibacillus turicensis* | Hn1-RCM-9 | 99.71 |
| ASV2427 | NR_114625.1 | 97.095 | *Paenibacillus turicensis* | Hn1-DRCA-7 | 99.71 |
| ASV2429 | NR_044524.1 | 98.644 | *Paenibacillus xylanexedens* | Hle3-TSA-4 | 99.43 |
| ASV2436 | NR_117034.1 | 96.755 | *Paenibacillus anaericanus* | Hn1-RCM-9 | 98.41 |
| ASV2449 | NR_181100.1 | 98.12 | *Companilactobacillus pabuli* | Sx3-MRS-3 | 98.44 |
| ASV2490 | NR_178542.1 | 98.3 | *Paenibacillus ihuae* | Hn1-YCFA-2 | 99.22 |
| ASV2497 | NR_025085.1 | 97.488 | *Paenibacillus granivorans* | Hn2-BLB-24 | 99.56 |
| ASV2512 | NR_181873.1 | 98.846 | *Pseudoneobacillus rhizosphaerae* | Nm3-NB-16 | 98.04 |
| ASV2516 | NR_113777.1 | 98.78 | *Neobacillus niacini* | Hb2-TSA-22 | 99.14 |
| ASV2517 | NR_025490.1 | 97.502 | *Paenibacillus agaridevorans* | Hn1-NB-21 | 99.21 |
| ASV2519 | NR_158107.1 | 96.989 | *Niallia endozanthoxylica* | Hle2-TSA-22 | 97.87 |
| ASV2521 | NR_114625.1 | 97.363 | *Paenibacillus turicensis* | Hn1-RCM-9 | 100 |
| ASV2524 | NR_114625.1 | 97.368 | *Paenibacillus turicensis* | Hn1-RCM-9 | 98.7 |
| ASV2526 | NR_151979.1 | 97.493 | *Niallia oryzisoli* | Hn1-YLS-4 | 98.06 |
| ASV2530 | NR_145559.1 | 97.94 | *Paenibacillus populi* | Hn1-NB-21 | 98.99 |
| ASV2531 | NR_029239.3 | 97.764 | *Clostridium chartatabidum* | Sx3-YLS-9 | 100 |
| ASV2532 | NR_025490.1 | 97.701 | *Paenibacillus agaridevorans* | Hn1-NB-21 | 97.48 |
| ASV2537 | NR_181553.1 | 95.37 | *Clostridium thailandense* | Hle3-YCFA-12 | 97.44 |
| ASV2541 | NR_149175.1 | 97.949 | *Bacillus mesophilus* | Sd1-NB-38 | 97.29 |
| ASV2553 | NR_025490.1 | 96.888 | *Paenibacillus agaridevorans* | Hn1-NB-21 | 97.41 |
| ASV2554 | NR_043220.1 | 97.689 | *Paenibacillus harenae* | Hb2-TSA-6 | 97.32 |
| ASV2556 | NR_042756.1 | 97.094 | *Paenibacillus barengoltzii* | Sx3-YCFA-9 | 97.25 |
| ASV2560 | NR_114625.1 | 96.7 | *Paenibacillus turicensis* | Hn1-RCM-9 | 98.62 |
| ASV2569 | NR_158107.1 | 97.546 | *Niallia endozanthoxylica* | Hb1-BLB-20 | 98.01 |
| ASV2572 | NR_041377.1 | 98.642 | *Neobacillus pocheonensis* | Hun7-YCFA-2 | 98.52 |
| ASV2577 | NR_148613.1 | 93.746 | *Paenibacillus cavernae* | Hn1-DRCA-1 | 98.65 |
| ASV2593 | NR_114085.1 | 98.289 | *Neobacillus drentensis* | Hb2-NB-3 | 98.68 |
| ASV2616 | NR_041468.1 | 96.804 | *Lentilactobacillus parafarraginis* | Sx1-YLS-15 | 97.6 |
| ASV2617 | NR_181100.1 | 97.047 | *Companilactobacillus pabuli* | Hn2-LBS-7 | 97.22 |
| ASV2618 | NR_115271.1 | 97.588 | *Lentilactobacillus kefiri* | Hle1-YLS-11 | 97.73 |
| ASV2623 | NR_029133.1 | 98.522 | *Lactiplantibacillus pentosus* | Hb3-SB-9 | 98.9 |
| ASV2625 | NR_029133.1 | 98.255 | *Lactiplantibacillus pentosus* | Sx3-M17-17 | 98.79 |
| ASV2634 | NR_041293.1 | 98.266 | *Lentilactobacillus buchneri* | Sx2-DRCA-12 | 100 |
| ASV2635 | NR_041293.1 | 97.931 | *Lentilactobacillus buchneri* | Sx2-DRCA-12 | 100 |
| ASV2637 | NR_041293.1 | 98.2 | *Lentilactobacillus buchneri* | Sx2-DRCA-12 | 100 |
| ASV2638 | NR_117987.1 | 97.265 | *Lacticaseibacillus paracasei* | Nm3-M17-5 | 97.64 |
| ASV2639 | NR_029133.1 | 97.317 | *Lactiplantibacillus pentosus* | Hb1-BLB-5 | 97.6 |
| ASV2650 | NR_112690.1 | 97.467 | *Lactiplantibacillus plantarum* | Nm3-LB-6 | 100 |
| ASV2664 | NR_113338.1 | 97.586 | *Lactiplantibacillus plantarum* | Hb1-BLB-14 | 97.99 |
| ASV2680 | NR_126193.1 | 97.368 | *Companilactobacillus furfuricola* | Sx1-MRS-9 | 97.83 |
| ASV2692 | NR_041293.1 | 97.667 | *Lentilactobacillus buchneri* | Sx2-DRCA-12 | 100 |
| ASV2693 | NR_042230.1 | 97.419 | *Lentilactobacillus kefiri* | Tj-MRS-11 | 97.69 |
| ASV2694 | NR_041293.1 | 98.732 | *Lentilactobacillus buchneri* | Sx2-DRCA-12 | 100 |
| ASV2696 | NR_029133.1 | 97.72 | *Lactiplantibacillus pentosus* | Hb1-BLB-5 | 98.03 |
| ASV2698 | NR_041293.1 | 98.599 | *Lentilactobacillus buchneri* | Sx2-DRCA-12 | 100 |
| ASV2701 | NR_041293.1 | 98.133 | *Lentilactobacillus buchneri* | Sx2-DRCA-12 | 100 |
| ASV2705 | NR_041293.1 | 97.999 | *Lentilactobacillus buchneri* | Sx2-DRCA-12 | 100 |
| ASV2709 | NR_113338.1 | 97.653 | *Lactiplantibacillus plantarum* | Hb1-BLB-14 | 98.06 |
| ASV2711 | NR_041293.1 | 98.597 | *Lentilactobacillus buchneri* | Sx2-DRCA-12 | 100 |
| ASV2721 | NR_115116.1 | 98.51 | *Lentilactobacillus parabuchneri* | Sx2-DRCA-12 | 100 |
| ASV2728 | NR_042230.1 | 97.594 | *Lentilactobacillus kefiri* | Hle1-YLS-11 | 98.26 |
| ASV2729 | NR_041584.1 | 97.113 | *Levilactobacillus senmaizukei* | Hb3-MRS-9 | 97.44 |
| ASV2731 | NR_029133.1 | 98.658 | *Lactiplantibacillus pentosus* | Sx3-TSA-8 | 99.03 |
| ASV2732 | NR_180275.1 | 98.789 | *Companilactobacillus zhongbaensis* | Sx3-MRS-3 | 97.74 |
| ASV2736 | NR_029133.1 | 98.388 | *Lactiplantibacillus pentosus* | Hb1-BLB-5 | 98.62 |
| ASV2754 | NR_025085.1 | 97.019 | *Paenibacillus granivorans* | Hn2-BLB-24 | 99.64 |
| ASV2765 | NR_074830.1 | 97.621 | *Conexibacter woesei* | Nm3-MRS-6 | 97.14 |
| ASV2771 | NR_179290.1 | 98.421 | *Companilactobacillus huachuanensis* | Sx3-MRS-15 | 98.7 |
| ASV2774 | NR_180286.1 | 97.139 | *Levilactobacillus angrenensis* | Hb3-MRS-9 | 97.59 |
| ASV2775 | NR_126193.1 | 97.069 | *Companilactobacillus furfuricola* | Nm3-LB-6 | 97.62 |
| ASV2776 | NR_025085.1 | 97.22 | *Paenibacillus granivorans* | Hn2-BLB-24 | 99.57 |
| ASV2780 | NR_113906.1 | 98.179 | *Enterococcus mundtii* | Nm2-YLS-3 | 97.96 |
| ASV2793 | NR_115116.1 | 97.848 | *Lentilactobacillus parabuchneri* | Hle1-MRS-6 | 99.13 |
| ASV2796 | NR_041293.1 | 98.532 | *Lentilactobacillus buchneri* | Sx2-DRCA-12 | 100 |
| ASV2804 | NR_112691.1 | 97.619 | *Liquorilactobacillus mali* | Sx2-DRCA-12 | 100 |
| ASV2823 | NR_026491.1 | 98.401 | *Clostridium disporicum* | Hle3-NB-14 | 100 |
| ASV2826 | NR_158107.1 | 97.265 | *Niallia endozanthoxylica* | Hb1-BLB-20 | 97.8 |
| ASV2849 | NR_133974.1 | 98.631 | *Peribacillus huizhouensis* | Sx1-NB-11 | 98.7 |
| ASV2859 | NR_158107.1 | 97.826 | *Niallia endozanthoxylica* | Hb1-BLB-20 | 98.37 |
| ASV2864 | NR_144696.1 | 96.446 | *Clostridium saudiense* | Hle3-NB-14 | 100 |
| ASV2876 | NR_179391.1 | 98.747 | *Clostridium jeddahitimonense* | Hle1-TSA-6 | 97.44 |
| ASV2886 | NR_043527.1 | 97.764 | *Sporosarcina soli* | Hb1-BLB-25 | 97.61 |
| ASV2887 | NR_146034.1 | 97.378 | *Cytobacillus depressus* | Hb1-BLB-20 | 98.44 |
| ASV2892 | NR_151979.1 | 96.811 | *Niallia oryzisoli* | Hb1-BLB-20 | 97.73 |
| ASV2900 | NR_151979.1 | 97.693 | *Niallia oryzisoli* | Hn1-YLS-4 | 98.28 |
| ASV2912 | NR_146034.1 | 97.447 | *Cytobacillus depressus* | Hb1-BLB-20 | 98.23 |
| ASV2917 | NR_137360.1 | 98.779 | *Mesobacillus campisalis* | Sd2-YLS-14 | 98.01 |
| ASV2921 | NR_158107.1 | 97.546 | *Niallia endozanthoxylica* | Hb1-BLB-20 | 97.94 |
| ASV2925 | NR_158107.1 | 97.475 | *Niallia endozanthoxylica* | Hb1-BLB-20 | 97.87 |
| ASV2936 | NR_151979.1 | 97.215 | *Niallia oryzisoli* | Hb1-BLB-20 | 97.51 |
| ASV2939 | NR_180510.1 | 97.639 | *Niallia alba* | Sd1-DRCA-19 | 97.83 |
| ASV2942 | NR_043527.1 | 97.835 | *Sporosarcina soli* | Sx1-LB-8 | 97.9 |
| ASV2945 | NR_151979.1 | 96.402 | *Niallia oryzisoli* | Hb1-BLB-20 | 97.38 |
| ASV2957 | NR_151979.1 | 97.015 | *Niallia oryzisoli* | Hb1-BLB-20 | 97.8 |
| ASV2961 | NR_116552.1 | 97.657 | *Ureibacillus sinduriensis* | Nm2-TSA-13 | 97.66 |
| ASV2964 | NR_041377.1 | 98.709 | *Neobacillus pocheonensis* | Hun7-YCFA-3 | 98.84 |
| ASV2969 | NR_134073.1 | 97.065 | *Lysinibacillus halotolerans* | Nm2-TSA-22 | 97.38 |
| ASV2978 | NR_040792.1 | 97.764 | *Lederbergia lenta* | Sd2-BLB-20 | 97.4 |
| ASV2987 | NR_136779.1 | 98.438 | *Lysinibacillus alkaliphilus* | Nm2-LB-19 | 97.69 |
| ASV2990 | NR_040792.1 | 98.711 | *Lederbergia lenta* | Sd2-BLB-20 | 99.58 |
| ASV2995 | NR_042274.1 | 98.236 | *Mesobacillus foraminis* | Sd2-YLS-14 | 99.79 |
| ASV2997 | NR_104923.1 | 98.297 | *Sporosarcina pasteurii* | Hb1-EMB-22 | 97 |
| ASV3002 | NR_151979.1 | 97.085 | *Niallia oryzisoli* | Hb1-BLB-20 | 98.16 |
| ASV3003 | NR_113881.1 | 98.174 | *Lysinibacillus odysseyi* | Sd1-M17-21 | 97.61 |
| ASV3008 | NR_117012.1 | 97.295 | *Paenibacillus uliginis* | Sx3-M17-1 | 97.26 |
| ASV3015 | NR_026336.1 | 96.451 | *Clostridium vincentii* | Sx1-LB-3 | 97.44 |
| ASV3017 | NR_144741.1 | 98.442 | *Bacillus mediterraneensis* | Sd2-YLS-14 | 97.09 |
| ASV3029 | NR_151979.1 | 97.422 | *Niallia oryzisoli* | Hb1-BLB-20 | 98.3 |
| ASV3036 | NR_025651.1 | 85.726 | *Anaerotignum lactatifermentans* | Nm3-BLB-20 | 97.37 |
| ASV3040 | NR_112632.1 | 97.079 | *Niallia circulans* | Sd1-YLS-23 | 98.24 |
| ASV3042 | NR_179659.1 | 96.809 | *Lysinibacillus timonensis* | Sx1-BLB-12 | 97.32 |
| ASV3052 | NR_115947.1 | 98.121 | *Paenibacillus donghaensis* | Sx3-EMB-20 | 98.27 |
| ASV3053 | NR_116955.1 | 97.817 | *Sporosarcina contaminans* | Nm2-BLB-12 | 99 |
| ASV3055 | NR_024817.1 | 97.898 | *Peribacillus asahii* | Sx3-NB-17 | 97.92 |
| ASV3060 | NR_181758.1 | 94.536 | *Clostridium simiarum* | Hun7-TSA-20 | 100 |
| ASV3066 | NR_116552.1 | 98.584 | *Ureibacillus sinduriensis* | Nm2-TSA-13 | 98.57 |
| ASV3071 | NR_135864.1 | 98.641 | *Ureibacillus acetophenoni* | Sd2-BLB-22 | 99.15 |
| ASV3072 | NR_043527.1 | 98.643 | *Sporosarcina soli* | Nm2-BLB-12 | 98.52 |
| ASV3079 | NR_181636.1 | 98.466 | *Paenibacillus tianjinensis* | Sx3-EMB-20 | 98.35 |
| ASV3087 | NR_132611.1 | 95.427 | *Tenuibacillus halotolerans* | Hun7-NB-6 | 100 |
| ASV3092 | NR_112628.1 | 97.087 | *Lysinibacillus fusiformis* | Sd2-RCM-7 | 97.36 |
| ASV3097 | NR_040792.1 | 97.897 | *Lederbergia lenta* | Sd2-BLB-20 | 98.24 |
| ASV3100 | NR_114093.1 | 98.302 | *Neobacillus bataviensis* | Sd2-YLS-3 | 98.55 |
| ASV3102 | NR_180162.1 | 98.578 | *Paenibacillus paeoniae* | Hn2-M17-6 | 97.93 |
| ASV3105 | NR_134171.1 | 95.349 | *Salinithrix halophila* | Nm3-YCFA-10 | 97.3 |
| ASV3113 | NR_171531.1 | 98.777 | *Siminovitchia acidinfaciens* | Nm3-NB-15 | 98.78 |
| ASV3114 | NR_179433.1 | 97.627 | *Paenibacillus rubinfantis* | Hn1-M17-11 | 97.53 |
| ASV3115 | NR_181636.1 | 98.022 | *Paenibacillus tianjinensis* | Hn2-NB-17 | 97.84 |
| ASV3117 | NR_179393.1 | 97.498 | *Neobacillus massiliamazoniensis* | Sd2-YLS-14 | 98.17 |
| ASV3118 | NR_117285.1 | 98.422 | *Cytobacillus oceanisediminis* | Hb2-LB-15 | 98.43 |
| ASV3120 | NR_116552.1 | 97.949 | *Ureibacillus sinduriensis* | Nm2-TSA-19 | 98.02 |
| ASV3138 | NR_178823.1 | 96.91 | *Clostridium thermarum* | Hle3-YCFA-12 | 97.37 |
| ASV3141 | NR_178489.1 | 97.976 | *Bacillus mesophilum* | Sd2-DRCA-18 | 98.24 |
| ASV3142 | NR_025626.1 | 97.897 | *Fredinandcohnia humi* | Sd1-M17-37 | 98.14 |
| ASV3143 | NR_126179.1 | 98.178 | *Clostridium swellfunianum* | Nm3-TSA-21 | 97.3 |
| ASV3144 | NR_114085.1 | 98.221 | *Neobacillus drentensis* | Hle3-TSA-26 | 98.26 |
| ASV3151 | NR_118411.1 | 97.727 | *Hazenella coriacea* | Hun7-NB-10 | 97.56 |
| ASV3163 | NR_029239.3 | 97.278 | *Clostridium chartatabidum* | Nm2-RCM-18 | 97.96 |
| ASV3177 | NR_145557.1 | 95.198 | *Oceanobacillus damuensis* | Nm3-LB-6 | 100 |
| ASV3186 | NR_115578.1 | 98.371 | *Bacillus benzoevorans* | Hb1-BLB-20 | 97.01 |
| ASV3193 | NR_145558.2 | 98.458 | *Oceanobacillus rekensis* | Sx2-EMB-15 | 100 |
| ASV3203 | NR_117285.1 | 98.625 | *Cytobacillus oceanisediminis* | Sx2-BLB-8 | 98.34 |
| ASV3204 | NR_044828.1 | 97.948 | *Bacillus benzoevorans* | Sd1-DRCA-21 | 97.58 |
| ASV3208 | NR_041293.1 | 97.997 | *Lentilactobacillus buchneri* | Sx2-DRCA-12 | 100 |
| ASV3214 | NR_115116.1 | 98.593 | *Lentilactobacillus parabuchneri* | Sx2-DRCA-12 | 100 |
| ASV3226 | NR_042514.1 | 96.789 | *Levilactobacillus namurensis* | Hb1-MRS-8 | 97.51 |
| ASV3227 | NR_042514.1 | 97.793 | *Levilactobacillus namurensis* | Hb1-MRS-8 | 98.58 |
| ASV3237 | NR_041468.1 | 97.603 | *Lentilactobacillus parafarraginis* | Sx1-YLS-15 | 98.44 |
| ASV3241 | NR_115116.1 | 97.93 | *Lentilactobacillus parabuchneri* | Sx2-DRCA-12 | 100 |
| ASV3245 | NR_041659.1 | 96.939 | *Lentilactobacillus rapi* | Sx2-DRCA-12 | 100 |
| ASV3246 | NR_149175.1 | 97.813 | *Bacillus mesophilus* | Sd1-NB-38 | 97.15 |
| ASV3255 | NR_041293.1 | 97.667 | *Lentilactobacillus buchneri* | Sx2-DRCA-12 | 100 |
| ASV3266 | NR_116238.1 | 98.183 | *Levilactobacillus brevis* | Tj-MRS-6 | 98.44 |
| ASV3268 | NR_115271.1 | 97.763 | *Lentilactobacillus kefiri* | Hle1-YLS-11 | 98.26 |
| ASV3269 | NR_042230.1 | 97.563 | *Lentilactobacillus kefiri* | Tj-MRS-11 | 97.85 |
| ASV3283 | NR_113338.1 | 98.322 | *Lactiplantibacillus plantarum* | Hb1-BLB-14 | 98.92 |
| ASV3292 | NR_104277.1 | 97.822 | *Paenibacillus daejeonensis* | Sd1-YCFA-29 | 97.97 |
| ASV3294 | NR_115271.1 | 97.589 | *Lentilactobacillus kefiri* | Hle1-YLS-11 | 98.11 |
| ASV3297 | NR_025045.1 | 98.591 | *Companilactobacillus kimchii* | Hb3-PYG-5 | 98.66 |
| ASV3310 | NR_116303.1 | 97.485 | *Paenibacillus camelliae* | Hn2-BLB-24 | 99.36 |
| ASV3311 | NR_109370.1 | 97.789 | *Companilactobacillus heilongjiangensis* | Sx2-DRCA-12 | 100 |
| ASV3312 | NR_025490.1 | 97.969 | *Paenibacillus agaridevorans* | Hn1-NB-21 | 99.13 |
| ASV3319 | NR_113338.1 | 97.055 | *Lactiplantibacillus plantarum* | Hb1-BLB-14 | 97.56 |
| ASV3322 | NR_136785.1 | 97.252 | *Lactiplantibacillus plajomi* | Hb1-BLB-14 | 97.63 |
| ASV3323 | NR_041293.1 | 97.2 | *Lentilactobacillus buchneri* | Sx2-DRCA-12 | 100 |
| ASV3324 | NR_145899.1 | 97.233 | *Lactiplantibacillus herbarum* | Nm3-LB-6 | 100 |
| ASV3325 | NR_113338.1 | 98.456 | *Lactiplantibacillus plantarum* | Sx1-DRCA-11 | 98.87 |
| ASV3326 | NR_113338.1 | 98.189 | *Lactiplantibacillus plantarum* | Hb1-BLB-14 | 98.42 |
| ASV3327 | NR_112690.1 | 98.08 | *Lactiplantibacillus plantarum* | Hb3-MRS-1 | 98.26 |
| ASV3328 | NR_181100.1 | 98.052 | *Companilactobacillus pabuli* | Sx3-MRS-3 | 98.37 |
| ASV3329 | NR_113338.1 | 98.724 | *Lactiplantibacillus plantarum* | Hb1-BLB-14 | 99.07 |
| ASV3330 | NR_025447.1 | 97.395 | *Lactiplantibacillus paraplantarum* | Hb3-MRS-1 | 98.42 |
| ASV3331 | NR_113338.1 | 96.51 | *Lactiplantibacillus plantarum* | Hb3-MRS-6 | 97.05 |
| ASV3334 | NR_112690.1 | 97.563 | *Lactiplantibacillus plantarum* | Hb3-MRS-1 | 98.19 |
| ASV3337 | NR_158107.1 | 97.265 | *Niallia endozanthoxylica* | Hb1-BLB-20 | 97.94 |
| ASV3338 | NR_163642.1 | 98.837 | *Paenibacillus mobilis* | Hle3-EMB-8 | 99.58 |
| ASV3348 | NR_113777.1 | 98.845 | *Neobacillus niacini* | Hun7-YCFA-3 | 99.42 |
| ASV3357 | NR_146034.1 | 97.308 | *Cytobacillus depressus* | Hb1-BLB-20 | 98.37 |
| ASV3361 | NR_158107.1 | 97.546 | *Niallia endozanthoxylica* | Hb1-BLB-20 | 98.09 |
| ASV3366 | NR_158107.1 | 97.475 | *Niallia endozanthoxylica* | Hb1-BLB-20 | 97.94 |
| ASV3367 | NR_165702.1 | 98.852 | *Paenibacillus seodonensis* | Hle3-M17-10 | 99.79 |
| ASV3369 | NR_163642.1 | 98.701 | *Paenibacillus mobilis* | Hun4-TSA-19 | 99.5 |
| ASV3371 | NR_165702.1 | 98.852 | *Paenibacillus seodonensis* | Hun4-NB-17 | 99.72 |
| ASV3374 | NR_133974.1 | 98.563 | *Peribacillus huizhouensis* | Sx3-NB-20 | 98.55 |
| ASV3380 | NR_025489.1 | 98.707 | *Paenibacillus agarexedens* | Hn2-TSA-30 | 99.78 |
| ASV3383 | NR_151979.1 | 96.676 | *Niallia oryzisoli* | Hb1-BLB-20 | 97.66 |
| ASV3385 | NR_158107.1 | 97.195 | *Niallia endozanthoxylica* | Hb1-BLB-20 | 97.87 |
| ASV3389 | NR_025085.1 | 96.339 | *Paenibacillus granivorans* | Hn2-BLB-24 | 98.04 |
| ASV3390 | NR_158107.1 | 97.337 | *Niallia endozanthoxylica* | Sd1-DRCA-21 | 98.01 |
| ASV3391 | NR_025489.1 | 98.103 | *Paenibacillus agarexedens* | Hn2-TSA-30 | 99.57 |
| ASV3398 | NR_112632.1 | 97.153 | *Niallia circulans* | Sd1-DRCA-21 | 97.79 |
| ASV3401 | NR_158107.1 | 97.546 | *Niallia endozanthoxylica* | Hb1-BLB-20 | 98.16 |
| ASV3403 | NR_043527.1 | 97.696 | *Sporosarcina soli* | Hb1-BLB-25 | 97.54 |
| ASV3404 | NR_025489.1 | 98.639 | *Paenibacillus agarexedens* | Hb2-TSA-6 | 99.93 |
| ASV3409 | NR_025489.1 | 98.442 | *Paenibacillus agarexedens* | Hn2-TSA-30 | 99.2 |
| ASV3415 | NR_151979.1 | 96.404 | *Niallia oryzisoli* | Hb1-BLB-20 | 97.44 |
| ASV3425 | NR_025489.1 | 98.639 | *Paenibacillus agarexedens* | Hb2-TSA-6 | 99.78 |
| ASV3426 | NR_181873.1 | 98.71 | *Pseudoneobacillus rhizosphaerae* | Nm3-NB-16 | 98.04 |
| ASV3435 | NR_025258.1 | 97.835 | *Lysinibacillus odysseyi* | Nm2-TSA-13 | 97.8 |
| ASV3437 | NR_024695.1 | 98.505 | *Neobacillus niacini* | Hun7-YCFA-3 | 98.92 |
| ASV3441 | NR_025489.1 | 98.038 | *Paenibacillus agarexedens* | Hn2-TSA-30 | 99.93 |
| ASV3443 | NR_117474.1 | 98.371 | *Peribacillus frigoritolerans* | Sx1-NB-11 | 98.7 |
| ASV3446 | NR_144696.1 | 96.237 | *Clostridium saudiense* | Nm3-TSA-21 | 100 |
| ASV3450 | NR_133974.1 | 98.154 | *Peribacillus huizhouensis* | Sx3-NB-20 | 98.4 |
| ASV3451 | NR_180378.1 | 97.833 | *Lysinibacillus antri* | Sd2-BLB-22 | 98.48 |
| ASV3452 | NR_040792.1 | 98.643 | *Lederbergia lenta* | Sd2-BLB-20 | 98.45 |
| ASV3453 | NR_043527.1 | 98.44 | *Sporosarcina soli* | Sx1-LB-8 | 98.16 |
| ASV3455 | NR_151979.1 | 97.829 | *Niallia oryzisoli* | Hn1-YLS-4 | 98.2 |
| ASV3461 | NR_179391.1 | 98.677 | *Clostridium jeddahitimonense* | Hle1-TSA-6 | 97.44 |
| ASV3462 | NR_137360.1 | 98.847 | *Mesobacillus campisalis* | Sd2-YLS-14 | 98.08 |
| ASV3470 | NR_151979.1 | 97.761 | *Niallia oryzisoli* | Sd1-DRCA-21 | 98.31 |
| ASV3475 | NR_151979.1 | 97.895 | *Niallia oryzisoli* | Hn1-YLS-4 | 98.28 |
| ASV3477 | NR_042274.1 | 98.236 | *Mesobacillus foraminis* | Sd2-YLS-14 | 99.93 |
| ASV3481 | NR_156041.1 | 98.645 | *Mesobacillus maritimus* | Hun7-YCFA-2 | 97.26 |
| ASV3492 | NR_044828.1 | 97.663 | *Bacillus benzoevorans* | Hb1-BLB-20 | 97.09 |
| ASV3504 | NR_112632.1 | 97.218 | *Niallia circulans* | Sd1-DRCA-21 | 97.65 |
| ASV3506 | NR_025258.1 | 98.311 | *Lysinibacillus odysseyi* | Nm2-TSA-19 | 97.61 |
| ASV3507 | NR_024695.1 | 97.758 | *Neobacillus niacini* | Nm3-NB-16 | 98.67 |
| ASV3508 | NR_146034.1 | 97.792 | *Cytobacillus depressus* | Hb1-BLB-20 | 98.3 |
| ASV3509 | NR_043015.1 | 98.641 | *Metabacillus litoralis* | Hb2-BLB-13 | 98.8 |
| ASV3511 | NR_029109.1 | 98.779 | *Paenibacillus xylanilyticus* | Hb2-M17-6 | 99.85 |
| ASV3513 | NR_025258.1 | 98.108 | *Lysinibacillus odysseyi* | Nm2-TSA-19 | 97.55 |
| ASV3518 | NR_117285.1 | 96.95 | *Cytobacillus oceanisediminis* | Hb2-M17-8 | 97.28 |
| ASV3522 | NR_135705.1 | 98.496 | *Paenibacillus endophyticus* | Hn1-BLB-37 | 97.61 |
| ASV3524 | NR_116955.1 | 97.82 | *Sporosarcina contaminans* | Sx1-LB-8 | 97.9 |
| ASV3527 | NR_109068.1 | 98.252 | *Neobacillus ginsengisoli* | Nm3-NB-16 | 99.65 |
| ASV3528 | NR_165716.1 | 98.349 | *Lysinibacillus yapensis* | Nm2-LB-19 | 98.13 |
| ASV3531 | NR_112632.1 | 97.288 | *Niallia circulans* | Nm2-M17-13 | 97.69 |
| ASV3532 | NR_043293.1 | 96.962 | *Paenibacillus alkaliterrae* | Hn2-TSA-30 | 97.25 |
| ASV3534 | NR_029239.3 | 97.486 | *Clostridium chartatabidum* | Nm2-RCM-18 | 98.11 |
| ASV3535 | NR_133702.1 | 98.276 | *Lederbergia panacisoli* | Sd2-BLB-20 | 97.82 |
| ASV3536 | NR_136779.1 | 98.37 | *Lysinibacillus alkaliphilus* | Nm2-LB-19 | 97.62 |
| ASV3539 | NR_113777.1 | 98.098 | *Neobacillus niacini* | Nm3-NB-16 | 98.32 |
| ASV3542 | NR_104923.1 | 97.481 | *Sporosarcina pasteurii* | Sd2-BLB-19 | 97.05 |
| ASV3548 | NR_116552.1 | 97.602 | *Ureibacillus sinduriensis* | Nm2-TSA-22 | 97.81 |
| ASV3549 | NR_114085.1 | 98.792 | *Neobacillus drentensis* | Sd1-NB-21 | 99.14 |
| ASV3551 | NR_042383.1 | 98.883 | *Shouchella plakortidis* | Hn1-BLB-2 | 99.06 |
| ASV3560 | NR_024817.1 | 97.965 | *Peribacillus asahii* | Hun7-TSA-6 | 97.85 |
| ASV3574 | NR_036951.1 | 98.626 | *Clostridium saccharobutylicum* | Sx3-YLS-9 | 100 |
| ASV3575 | NR_145585.1 | 97.626 | *Peribacillus gossypii* | Hun4-DRCA-18 | 97.61 |
| ASV3587 | NR_151979.1 | 97.013 | *Niallia oryzisoli* | Hb1-BLB-20 | 97.8 |
| ASV3588 | NR_025882.1 | 98.849 | *Paenibacillus amylolyticus* | Hle3-LB-10 | 99.58 |
| ASV3589 | NR_044538.1 | 98.492 | *Robertmurraya korlensis* | Sx2-BLB-8 | 97.43 |
| ASV3595 | NR_026336.1 | 97.006 | *Clostridium vincentii* | Hle3-NB-14 | 100 |
| ASV3599 | NR_114085.1 | 98.507 | *Neobacillus drentensis* | Hun7-YCFA-3 | 98.99 |
| ASV3600 | NR_109140.1 | 98.187 | *Mesobacillus persicus* | Sd1-NB-30 | 97.8 |
| ASV3601 | NR_112632.1 | 97.351 | *Niallia circulans* | Sd1-DRCA-21 | 97.79 |
| ASV3602 | NR_144741.1 | 98.374 | *Bacillus mediterraneensis* | Hun7-YCFA-2 | 97.04 |
| ASV3611 | NR_156041.1 | 98.813 | *Mesobacillus maritimus* | Sd2-YLS-14 | 97.35 |
| ASV3615 | NR_134171.1 | 93.023 | *Salinithrix halophila* | Hun7-YCFA-1 | 100 |
| ASV3618 | NR_104741.1 | 96.655 | *Clostridium tarantellae* | Hun4-LB-25 | 100 |
| ASV3619 | NR_151979.1 | 97.559 | *Niallia oryzisoli* | Sd1-DRCA-21 | 98.38 |
| ASV3621 | NR_151979.1 | 98.438 | *Niallia oryzisoli* | Hun7-RCM-12 | 98.09 |
| ASV3623 | NR_108831.1 | 98.43 | *Peribacillus endoradicis* | Sx1-LB-2 | 97.37 |
| ASV3625 | NR_028920.1 | 97.255 | *Clostridium uliginosum* | Sd1-YCFA-18 | 100 |
| ASV3637 | NR_144724.1 | 94.589 | *Desnuesiella massiliensis* | Hle3-NB-14 | 97.44 |
| ASV3640 | NR_041377.1 | 98.166 | *Neobacillus pocheonensis* | Nm3-NB-16 | 98.67 |
| ASV3641 | NR_029239.3 | 97.275 | *Clostridium chartatabidum* | Hun4-DRCA-15 | 97.62 |
| ASV3642 | NR_151979.1 | 97.217 | *Niallia oryzisoli* | Hb1-BLB-20 | 97.58 |
| ASV3644 | NR_112632.1 | 97.692 | *Niallia circulans* | Nm2-YCFA-4 | 98.26 |
| ASV3647 | NR_113244.1 | 98.818 | *Clostridium butyricum* | Hle3-NB-14 | 97.62 |
| ASV3654 | NR_147397.1 | 97.76 | *Bacillus marasmi* | Hun7-YCFA-2 | 98.22 |
| ASV3655 | NR_148273.1 | 98.236 | *Bacillus haikouensis* | Hun7-TSA-20 | 97.65 |
| ASV3656 | NR_117012.1 | 97.365 | *Paenibacillus uliginis* | Sx3-M17-1 | 97.34 |
| ASV3658 | NR_134073.1 | 97.273 | *Lysinibacillus halotolerans* | Sx1-BLB-12 | 97.61 |
| ASV3659 | NR_112625.1 | 98.432 | *Cohnella panacarvi* | Nm2-TSA-13 | 97.56 |
| ASV3661 | NR_044428.1 | 98.847 | *Paenibacillus taichungensis* | Hn1-TSA-11 | 99.21 |
| ASV3663 | NR_145559.1 | 98.352 | *Paenibacillus populi* | Hn1-NB-21 | 99.35 |
| ASV3664 | NR_145585.1 | 98.394 | *Peribacillus gossypii* | Sx3-NB-17 | 98.07 |
| ASV3667 | NR_041491.1 | 97.262 | *Paenibacillus nanensis* | Hn2-M17-6 | 97.63 |
| ASV3682 | NR_151979.1 | 97.015 | *Niallia oryzisoli* | Hb1-BLB-20 | 98.09 |
| ASV3684 | NR_144724.1 | 95.649 | *Desnuesiella massiliensis* | Hle2-YLS-12 | 97.44 |
| ASV3699 | NR_147741.1 | 97.831 | *Paenibacillus terreus* | Sd2-LB-12 | 99.43 |
| ASV3705 | NR_041491.1 | 97.127 | *Paenibacillus nanensis* | Hn2-M17-6 | 97.98 |
| ASV3708 | NR_109116.1 | 98.886 | *Metabacillus halosaccharovorans* | Sd2-DRCA-4 | 99.34 |
| ASV3719 | NR_114085.1 | 98.58 | *Neobacillus drentensis* | Sd2-M17-18 | 98.73 |
| ASV3725 | NR_159149.1 | 97.147 | *Paenibacillus albidus* | Hn2-NB-17 | 98.12 |
| ASV3726 | NR_042648.1 | 97.031 | *Solibacillus cecembensis* | Sd1-YLS-18 | 97.22 |
| ASV3727 | NR_026336.1 | 96.797 | *Clostridium vincentii* | Hb2-LB-21 | 100 |
| ASV3729 | NR_149252.1 | 98.043 | *Fredinandcohnia onubensis* | Sx2-YCFA-17 | 97.76 |
| ASV3731 | NR_041491.1 | 96.92 | *Paenibacillus nanensis* | Hn2-M17-6 | 97.77 |
| ASV3732 | NR_044828.1 | 98.089 | *Bacillus benzoevorans* | Nm2-M17-13 | 97.55 |
| ASV3747 | NR_044538.1 | 98.152 | *Robertmurraya korlensis* | Hun7-RCM-12 | 97.94 |
| ASV3748 | NR_149778.1 | 98.7 | *Paenibacillus cucumis* | Hb2-BLB-25 | 98.99 |
| ASV3751 | NR_158107.1 | 97.759 | *Niallia endozanthoxylica* | Hb1-BLB-20 | 97.94 |
| ASV3752 | NR_116851.1 | 98.237 | *Priestia koreensis* | Hun7-RCM-12 | 97.73 |
| ASV3753 | NR_136792.1 | 98.335 | *Robertmurraya crescens* | Hn2-LB-19 | 97.54 |
| ASV3771 | NR_112632.1 | 97.624 | *Niallia circulans* | Nm2-M17-13 | 97.98 |
| ASV3775 | NR_116552.1 | 97.24 | *Ureibacillus sinduriensis* | Sd2-BLB-22 | 98.27 |
| ASV3776 | NR_135864.1 | 98.439 | *Ureibacillus acetophenoni* | Sd2-BLB-22 | 98.93 |
| ASV3786 | NR_117028.1 | 96.198 | *Caldibacillus thermoamylovorans* | Nm2-M17-10 | 97.06 |
| ASV3789 | NR_144724.1 | 95.168 | *Desnuesiella massiliensis* | Hle3-NB-14 | 97.44 |
| ASV3792 | NR_145633.1 | 97.519 | *Cytobacillus solani* | Sd2-DRCA-18 | 98.01 |
| ASV3793 | NR_025373.1 | 97.763 | *Margalitia shackletonii* | Hn2-DRCA-1 | 98.02 |
| ASV3806 | NR_126270.1 | 98.561 | *Oceanobacillus luteolus* | Nm3-LB-6 | 100 |
| ASV3812 | NR_113881.1 | 98.307 | *Lysinibacillus odysseyi* | Nm2-TSA-22 | 97.96 |
| ASV3818 | NR_148658.1 | 97.401 | *Paenibacillus radicis* | Hb2-TSA-6 | 97.67 |
| ASV3819 | NR_025490.1 | 98.172 | *Paenibacillus agaridevorans* | Hn1-NB-21 | 98.56 |
| ASV3820 | NR_042274.1 | 98.375 | *Mesobacillus foraminis* | Sd2-YLS-14 | 97.86 |
| ASV3824 | NR_041293.1 | 97.2 | *Lentilactobacillus buchneri* | Sx2-DRCA-12 | 100 |
| ASV3832 | NR_145559.1 | 98.489 | *Paenibacillus populi* | Hn1-NB-21 | 99.49 |
| ASV3834 | NR_114625.1 | 96.561 | *Paenibacillus turicensis* | Hn2-M17-8 | 99.78 |
| ASV3835 | NR_025490.1 | 97.908 | *Paenibacillus agaridevorans* | Hn1-NB-21 | 99.28 |
| ASV3836 | NR_118463.1 | 97.614 | *Paracoccus siganidrum* | Hb1-EMB-21 | 100 |
| ASV3847 | NR_029133.1 | 98.859 | *Lactiplantibacillus pentosus* | Hb3-SB-9 | 99.29 |
| ASV3848 | NR_029133.1 | 97.72 | *Lactiplantibacillus pentosus* | Tj-MRS-5 | 98.05 |
| ASV3849 | NR_041293.1 | 97.333 | *Lentilactobacillus buchneri* | Sx2-DRCA-12 | 100 |
| ASV3850 | NR_042230.1 | 97.634 | *Lentilactobacillus kefiri* | Tj-MRS-11 | 97.77 |
| ASV3851 | NR_041293.1 | 97.6 | *Lentilactobacillus buchneri* | Sx2-DRCA-12 | 100 |
| ASV3853 | NR_029133.1 | 98.322 | *Lactiplantibacillus pentosus* | Tj-MRS-5 | 98.7 |
| ASV3854 | NR_041293.1 | 96.8 | *Lentilactobacillus buchneri* | Nm3-LB-6 | 100 |
| ASV3855 | NR_041293.1 | 98.532 | *Lentilactobacillus buchneri* | Sx2-EMB-15 | 100 |
| ASV3856 | NR_041293.1 | 98.199 | *Lentilactobacillus buchneri* | Hle1-MRS-6 | 99.21 |
| ASV3857 | NR_145899.1 | 97.307 | *Lactiplantibacillus herbarum* | Hb3-MRS-1 | 98.03 |
| ASV3858 | NR_029133.1 | 97.517 | *Lactiplantibacillus pentosus* | Hb3-PYG-8 | 97.43 |
| ASV3859 | NR_112690.1 | 98.364 | *Lactiplantibacillus plantarum* | Nm3-LB-6 | 100 |
| ASV3863 | NR_025045.1 | 98.322 | *Companilactobacillus kimchii* | Hb3-PYG-5 | 98.38 |
| ASV3869 | NR_041293.1 | 98.532 | *Lentilactobacillus buchneri* | Sx2-DRCA-12 | 100 |
| ASV3870 | NR_041293.1 | 98.397 | *Lentilactobacillus buchneri* | Sx2-DRCA-12 | 100 |
| ASV3872 | NR_042677.1 | 97.561 | *Liquorilactobacillus cacaonum* | Nm3-M17-5 | 97.21 |
| ASV3873 | NR_116238.1 | 98.322 | *Levilactobacillus brevis* | Tj-MRS-6 | 98.66 |
| ASV3874 | NR_041293.1 | 96.6 | *Lentilactobacillus buchneri* | Sx2-DRCA-12 | 100 |
| ASV3875 | NR_042230.1 | 97.801 | *Lentilactobacillus kefiri* | Hb3-MRS-8 | 98.11 |
| ASV3881 | NR_041293.1 | 98.798 | *Lentilactobacillus buchneri* | Sx2-TSA-22 | 98.99 |
| ASV3887 | NR_041468.1 | 98.33 | *Lentilactobacillus parafarraginis* | Sx1-RCM-4 | 98.63 |
| ASV3888 | NR_041468.1 | 98.331 | *Lentilactobacillus parafarraginis* | Sx1-RCM-4 | 98.7 |
| ASV3893 | NR_113777.1 | 97.692 | *Neobacillus niacini* | Nm3-NB-16 | 98.11 |
| ASV3894 | NR_043557.1 | 98.632 | *Clostridium aciditolerans* | Sx3-YLS-9 | 100 |
| ASV3895 | NR_122090.1 | 98.05 | *Clostridium chromiireducens* | Sx3-YLS-9 | 100 |
| ASV3905 | NR_113338.1 | 98.794 | *Lactiplantibacillus plantarum* | Hb1-BLB-14 | 99.43 |
| ASV3906 | NR_113338.1 | 97.189 | *Lactiplantibacillus plantarum* | Hb1-BLB-14 | 97.71 |
| ASV3913 | NR_029133.1 | 97.25 | *Lactiplantibacillus pentosus* | Hb3-DRCA-6 | 97.55 |
| ASV3914 | NR_041293.1 | 97.067 | *Lentilactobacillus buchneri* | Nm3-LB-6 | 100 |
| ASV3916 | NR_029133.1 | 98.456 | *Lactiplantibacillus pentosus* | Hb1-BLB-5 | 98.83 |
| ASV3917 | NR_041293.1 | 98.397 | *Lentilactobacillus buchneri* | Hle1-TSA-33 | 98.4 |
| ASV3920 | NR_134112.1 | 98.687 | *Paenibacillus dongdonensis* | Hb2-BLB-21 | 99.06 |
| ASV3921 | NR_114625.1 | 97.301 | *Paenibacillus turicensis* | Hn1-RCM-9 | 98.62 |
| ASV3925 | NR_025490.1 | 98.579 | *Paenibacillus agaridevorans* | Hn1-NB-21 | 99.57 |
| ASV3943 | NR_025045.1 | 98.456 | *Companilactobacillus kimchii* | Hb3-PYG-5 | 98.52 |
| ASV3946 | NR_114398.1 | 97.653 | *Companilactobacillus farciminis* | Sx3-MRS-3 | 98.38 |
| ASV3952 | NR_112693.1 | 98.117 | *Ligilactobacillus acidipiscis* | Hun1-MRS-2 | 98.71 |
| ASV3963 | NR_026140.1 | 98.779 | *Alkalihalobacillus clausii* | Sd2-BLB-23 | 99.93 |
| ASV3965 | NR_026336.1 | 98.677 | *Clostridium vincentii* | Sd1-DRCA-21 | 100 |
| ASV3966 | NR_115116.1 | 98.51 | *Lentilactobacillus parabuchneri* | Sx2-DRCA-12 | 100 |
| ASV3972 | NR_116238.1 | 98.531 | *Levilactobacillus brevis* | Hb1-TSA-13 | 98.79 |
| ASV3973 | NR_151979.1 | 97.149 | *Niallia oryzisoli* | Hb1-BLB-20 | 97.73 |
| ASV3980 | NR_042057.1 | 98.469 | *Pediococcus acidilactici* | Sx3-M17-14 | 98.85 |
| ASV3981 | NR_114398.1 | 97.649 | *Companilactobacillus farciminis* | Sx3-MRS-15 | 98.16 |
| ASV3982 | NR_159230.1 | 97.047 | *Companilactobacillus musae* | Hb3-MRS-6 | 97.81 |
| ASV3987 | NR_113338.1 | 96.714 | *Lactiplantibacillus plantarum* | Tj-SB-4 | 97.01 |
| ASV3988 | NR_109452.1 | 98.085 | *Levilactobacillus yonginensis* | Sx3-RCM-7 | 98.75 |
| ASV3989 | NR_029133.1 | 98.724 | *Lactiplantibacillus pentosus* | Hb3-MRS-1 | 99.29 |
| ASV3990 | NR_116238.1 | 97.66 | *Levilactobacillus brevis* | Hb1-M17-1 | 98.18 |
| ASV3992 | NR_145899.1 | 97.579 | *Lactiplantibacillus herbarum* | Hb3-MRS-1 | 98.03 |
| ASV3994 | NR_116238.1 | 97.209 | *Levilactobacillus brevis* | Hb1-M17-2 | 97.74 |
| ASV4005 | NR_041293.1 | 97.4 | *Lentilactobacillus buchneri* | Sx2-DRCA-12 | 100 |
| ASV4006 | NR_041293.1 | 97 | *Lentilactobacillus buchneri* | Sx2-DRCA-12 | 100 |
| ASV4007 | NR_042230.1 | 97.491 | *Lentilactobacillus kefiri* | Tj-MRS-11 | 97.77 |
| ASV4008 | NR_145899.1 | 97.158 | *Lactiplantibacillus herbarum* | Hb3-MRS-1 | 97.87 |
| ASV4009 | NR_041293.1 | 98.666 | *Lentilactobacillus buchneri* | Sx2-DRCA-12 | 100 |
| ASV4011 | NR_113338.1 | 97.586 | *Lactiplantibacillus plantarum* | Hb1-BLB-14 | 97.78 |
| ASV4012 | NR_113338.1 | 98.792 | *Lactiplantibacillus plantarum* | Hb1-BLB-14 | 99.28 |
| ASV4013 | NR_112691.1 | 97.561 | *Liquorilactobacillus mali* | Hb1-LBS-1 | 97.66 |
| ASV4014 | NR_112690.1 | 96.727 | *Lactiplantibacillus plantarum* | Hb3-MRS-1 | 97.24 |
| ASV4016 | NR_181100.1 | 96.917 | *Companilactobacillus pabuli* | Hb3-MRS-6 | 98.04 |
| ASV4021 | NR_041293.1 | 97.198 | *Lentilactobacillus buchneri* | Sx2-DRCA-12 | 100 |
| ASV4022 | NR_179290.1 | 98.421 | *Companilactobacillus huachuanensis* | Sx3-MRS-15 | 98.7 |
| ASV4024 | NR_126193.1 | 97.368 | *Companilactobacillus furfuricola* | Sx3-YCFA-6 | 100 |
| ASV4031 | NR_180275.1 | 97.849 | *Companilactobacillus zhongbaensis* | Hb3-PYG-5 | 97.1 |
| ASV4034 | NR_114621.1 | 97.502 | *Paenibacillus turicensis* | Hn1-RCM-9 | 99.2 |
| ASV4038 | NR_179290.1 | 97.918 | *Companilactobacillus huachuanensis* | Hn2-LBS-6 | 98.4 |
| ASV4041 | NR_181134.1 | 98.59 | *Levilactobacillus tujiorum* | Sx3-NB-1 | 97.86 |
| ASV4044 | NR_136785.1 | 96.722 | *Lactiplantibacillus plajomi* | Hb1-BLB-14 | 97.21 |
| ASV4045 | NR_113338.1 | 98.727 | *Lactiplantibacillus plantarum* | Hb1-BLB-14 | 99.35 |
| ASV4054 | NR_042230.1 | 97.634 | *Lentilactobacillus kefiri* | Tj-MRS-11 | 97.93 |
| ASV4057 | NR_041293.1 | 97.467 | *Lentilactobacillus buchneri* | Sx2-EMB-15 | 100 |
| ASV4059 | NR_029133.1 | 97.787 | *Lactiplantibacillus pentosus* | Hb1-BLB-5 | 98.11 |
| ASV4061 | NR_041293.1 | 98.664 | *Lentilactobacillus buchneri* | Hb3-MRS-8 | 98.69 |
| ASV4062 | NR_136785.1 | 97.118 | *Lactiplantibacillus plajomi* | Hb1-BLB-14 | 97.2 |
| ASV4066 | NR_113338.1 | 98.725 | *Lactiplantibacillus plantarum* | Hb1-BLB-14 | 99.21 |
| ASV4072 | NR_041293.1 | 97.397 | *Lentilactobacillus buchneri* | Hb3-MRS-8 | 97.39 |
| ASV4089 | NR_113338.1 | 98.322 | *Lactiplantibacillus plantarum* | Hb1-BLB-14 | 98.78 |
| ASV4090 | NR_145899.1 | 97.307 | *Lactiplantibacillus herbarum* | Nm3-LB-6 | 100 |
| ASV4092 | NR_042230.1 | 97.348 | *Lentilactobacillus kefiri* | Hle1-YLS-11 | 97.81 |
| ASV4093 | NR_113338.1 | 98.59 | *Lactiplantibacillus plantarum* | Hb1-BLB-14 | 98.99 |
| ASV4116 | NR_178248.1 | 97.248 | *Companilactobacillus formosensis* | Hn2-LBS-7 | 97.22 |
| ASV4119 | NR_042514.1 | 98.118 | *Levilactobacillus namurensis* | Hb1-MRS-8 | 98.23 |
| ASV4141 | NR_041293.1 | 97.267 | *Lentilactobacillus buchneri* | Sx2-DRCA-12 | 100 |
| ASV4142 | NR_042514.1 | 97.383 | *Levilactobacillus namurensis* | Hb1-MRS-8 | 97.38 |
| ASV4145 | NR_136785.1 | 96.656 | *Lactiplantibacillus plajomi* | Hb1-BLB-14 | 97.13 |
| ASV4148 | NR_113906.1 | 97.104 | *Enterococcus mundtii* | Sx1-MRS-9 | 97.83 |
| ASV4152 | NR_115116.1 | 98.51 | *Lentilactobacillus parabuchneri* | Sx2-DRCA-12 | 100 |
| ASV4153 | NR_042230.1 | 97.419 | *Lentilactobacillus kefiri* | Hle1-YLS-11 | 97.81 |
| ASV4154 | NR_041293.1 | 97.999 | *Lentilactobacillus buchneri* | Sx2-DRCA-12 | 100 |
| ASV4158 | NR_041293.1 | 96.933 | *Lentilactobacillus buchneri* | Sx2-DRCA-12 | 100 |
| ASV4163 | NR_180289.1 | 97.077 | *Levilactobacillus tongjiangensis* | Hb1-M17-1 | 97.3 |
| ASV4164 | NR_136785.1 | 97.319 | *Lactiplantibacillus plajomi* | Hb1-BLB-14 | 97.2 |
| ASV4165 | NR_109452.1 | 97.66 | *Levilactobacillus yonginensis* | Hb3-MRS-1 | 97.71 |
| ASV4175 | NR_042243.1 | 97.649 | *Levilactobacillus hammesii* | Nm2-DRCA-3 | 97.25 |
| ASV4179 | NR_025882.1 | 98.781 | *Paenibacillus amylolyticus* | Hle3-LB-10 | 99.51 |
| ASV4190 | NR_036950.1 | 98.406 | *Clostridium saccharoperbutylacetonicum* | Hle3-NB-14 | 97.62 |
| ASV4191 | NR_025243.1 | 98.485 | *Cedecea davisae* | Hb1-NB-21 | 97.11 |
| ASV4196 | NR_113777.1 | 98.845 | *Neobacillus niacini* | Hun7-YCFA-3 | 99.57 |
| ASV4206 | NR_117285.1 | 97.99 | *Cytobacillus oceanisediminis* | Sd1-DRCA-19 | 98.9 |
| ASV4208 | NR_041293.1 | 97.2 | *Lentilactobacillus buchneri* | Sx2-DRCA-12 | 100 |
| ASV4209 | NR_041293.1 | 98.399 | *Lentilactobacillus buchneri* | Sx2-DRCA-12 | 100 |
| ASV4218 | NR_116873.1 | 98.129 | *Priestia megaterium* | Hun7-M17-17 | 98.35 |
| ASV4219 | NR_113265.1 | 97.493 | *Bacillus subtilis* | Hle1-YLS-6 | 98.08 |
| ASV4222 | NR_024696.1 | 98.845 | *Bacillus vallismortis* | Nm2-NB-19 | 98.79 |
| ASV4224 | NR_024931.1 | 97.874 | *Bacillus spizizenii* | Hun4-NB-8 | 97.92 |
| ASV4230 | NR_041293.1 | 97.6 | *Lentilactobacillus buchneri* | Sx2-DRCA-12 | 100 |
| ASV4231 | NR_029133.1 | 98.792 | *Lactiplantibacillus pentosus* | Hb3-SB-9 | 99.21 |
| ASV4232 | NR_145899.1 | 97.233 | *Lactiplantibacillus herbarum* | Nm3-LB-6 | 100 |
| ASV4241 | NR_041293.1 | 98.065 | *Lentilactobacillus buchneri* | Sx2-DRCA-12 | 100 |
| ASV4262 | NR_115116.1 | 98.013 | *Lentilactobacillus parabuchneri* | Sx2-DRCA-12 | 100 |
| ASV4264 | NR_112693.1 | 97.915 | *Ligilactobacillus acidipiscis* | Sd1-MRS-1 | 98.61 |

| **Table S4. Sanger sequencing results for novel strains of alBM** | | | |  |  |  |  |  |
| --- | --- | --- | --- | --- | --- | --- | --- | --- |
| **Direct ID** | **NCBI accession** | **NCBI blast** | | | | **Local Blast** | | |
|  |  | **16S rRNA**  **gene sequence**  **identity (%)** | **Taxonomy** | **Query length** | **E-value** | **alBM ID** | **Local identity (%)** | **Query length** |
| ASV336 | NR_025085.1 | 96.17 | *Paenibacillus* sp. | 1478 | 0 | Sd2-BLB-15 | 97.41 | 1392 |
| ASV350 | NR_114625.1 | 96.51 | *Paenibacillus* sp. | 1475 | 0 | Hn1-DRCA-7 | 99.43 | 1395 |
| ASV351 | NR_114625.1 | 94.44 | *Paenibacillus* sp. | 1475 | 0 | Hn1-RCM-10 | 99.72 | 1478 |
| ASV794 | NR_117690.2 | 93.14 | *Oxobacter* sp. | 1436 | 0 | Hun4-DRCA-18 | 100.00 | 1383 |
| ASV889 | NR_148613.1 | 93.89 | *Paenibacillus* sp. | 1467 | 0 | Hn1-DRCA-1 | 99.43 | 1400 |
| ASV1541 | NR_114625.1 | 96.49 | *Paenibacillus* sp. | 1475 | 0 | Hn1-RCM-10 | 99.65 | 1478 |
| ASV1545 | NR_148613.1 | 93.75 | *Paenibacillus* sp. | 1467 | 0 | Hn1-DRCA-1 | 99.43 | 1400 |
| ASV1546 | NR_148613.1 | 94.02 | *Paenibacillus* sp. | 1467 | 0 | Hn1-DRCA-1 | 99.57 | 1400 |
| ASV1610 | NR_117690.2 | 94.66 | *Oxobacter* sp. | 1435 | 0 | Hun4-DRCA-18 | 100.00 | 1383 |
| ASV1981 | NR_114625.1 | 96.56 | *Paenibacillus* sp. | 1475 | 0 | Hn1-YLS-16 | 99.78 | 1466 |
| ASV2092 | NR_117690.2 | 93.62 | *Oxobacter* sp. | 1437 | 0 | Hun4-DRCA-18 | 100.00 | 1383 |
| ASV2177 | NR_117690.2 | 94.18 | *Oxobacter* sp. | 1435 | 0 | Hun4-DRCA-18 | 100.00 | 1383 |
| ASV2332 | NR_117689.2 | 92.27 | *Oxobacter* sp. | 1448 | 0 | Hun4-DRCA-18 | 100.00 | 1383 |
| ASV2422 | NR_114625.1 | 96.49 | *Paenibacillus* sp. | 1476 | 0 | Hn1-RCM-10 | 99.51 | 1478 |
| ASV2436 | NR_117034.1 | 96.76 | *Paenibacillus* sp. | 1476 | 0 | Hn1-DRCA-7 | 98.26 | 1395 |
| ASV2560 | NR_114625.1 | 96.70 | *Paenibacillus* sp. | 1475 | 0 | Hn1-RCM-10 | 98.53 | 1478 |
| ASV2577 | NR_148613.1 | 93.75 | *Paenibacillus* sp. | 1467 | 0 | Hn1-DRCA-1 | 98.65 | 1400 |
| ASV3618 | NR_104741.1 | 96.66 | *Clostridium* sp. | 1432 | 0 | Hun4-DRCA-18 | 97.44 | 1383 |
| ASV3686 | NR_137216.1 | 86.87 | *Brassicibacter* sp. | 1442 | 0 | Hn1-DRCA-1 | 97.44 | 1400 |
| ASV3834 | NR_114625.1 | 96.56 | *Paenibacillus* sp. | 1476 | 0 | Hn1-RCM-10 | 99.65 | 1478 |

| **Table S5. Statistics and quality-control information for the sequence data** | | | | | | | | | | | | | | | | | | |
| --- | --- | --- | --- | --- | --- | --- | --- | --- | --- | --- | --- | --- | --- | --- | --- | --- | --- | --- |
| **Sample** | **Raw**  **Reads** | **Raw**  **Bases** | **Average**  **Length** | **Raw**  **Q20**  **(%)** | **Raw**  **Q30**  **(%)** | **Raw GC**  **content**  **(%)** | **Clean**  **Reads** | **Clean**  **Bases** | **Average**  **Length** | **Clean**  **Q20**  **(%)** | **Clean**  **Q30**  **(%)** | **Clean**  **GC content**  **(%)** | **Contigs** | **Contigs**  **bases**  **(bp)** | **Min**  **(bp)** | **Max**  **(bp)** | **N50**  **(bp）** | **N9**  **(bp)** |
| A1 | 113732214 | 17173564314 | 151 | 0.9711 | 0.9222 | 0.4924 | 112010818 | 16886560713 | 150 | 0.9753 | 0.9275 | 0.4921 | 579463 | 391731133 | 300 | 150250 | 701 | 354 |
| A11 | 101654530 | 15349834030 | 151 | 0.9673 | 0.9136 | 0.4582 | 100159414 | 15102510593 | 150 | 0.9715 | 0.9189 | 0.4579 | 303542 | 224951498 | 300 | 371836 | 806 | 362 |
| A13 | 117987704 | 17816143304 | 151 | 0.9685 | 0.9159 | 0.4398 | 116265280 | 17539205503 | 150 | 0.9727 | 0.9212 | 0.4394 | 20069 | 20792333 | 300 | 209550 | 1796 | 381 |
| A15 | 107551740 | 16240312740 | 151 | 0.9661 | 0.9111 | 0.4427 | 105833724 | 15966862620 | 150 | 0.9706 | 0.9166 | 0.4421 | 29467 | 30075478 | 300 | 209551 | 1671 | 384 |
| A19 | 104331252 | 15754019052 | 151 | 0.9667 | 0.9132 | 0.4999 | 102520462 | 15455873232 | 150 | 0.9716 | 0.9194 | 0.4995 | 701415 | 500239585 | 300 | 179101 | 767 | 356 |
| A23 | 111719050 | 16869576550 | 151 | 0.9664 | 0.9119 | 0.4312 | 109825550 | 16553696457 | 150 | 0.9712 | 0.9178 | 0.4304 | 183364 | 175969001 | 300 | 364330 | 1346 | 384 |
| A25 | 119458962 | 18038303262 | 151 | 0.9761 | 0.9345 | 0.5257 | 117878340 | 17720047800 | 150 | 0.98 | 0.9394 | 0.5255 | 654814 | 444264812 | 300 | 170120 | 715 | 352 |
| A27 | 93603108 | 14134069308 | 151 | 0.971 | 0.9245 | 0.5367 | 91731928 | 13741848927 | 149 | 0.9766 | 0.9316 | 0.5351 | 676783 | 459746521 | 300 | 441956 | 710 | 351 |
| A35 | 112213238 | 16944198938 | 151 | 0.9698 | 0.918 | 0.4861 | 110834984 | 16706979670 | 150 | 0.9732 | 0.9223 | 0.4861 | 461153 | 335063927 | 300 | 628538 | 778 | 360 |
| A37 | 111373794 | 16817442894 | 151 | 0.9671 | 0.9134 | 0.4178 | 109521296 | 16516613943 | 150 | 0.9719 | 0.9193 | 0.4172 | 114434 | 119963271 | 300 | 140551 | 1699 | 399 |
| A39 | 105522298 | 15933866998 | 151 | 0.9688 | 0.9172 | 0.4634 | 104012148 | 15635343243 | 150 | 0.973 | 0.9224 | 0.4628 | 307243 | 243451786 | 300 | 362777 | 920 | 363 |
| A41 | 111302322 | 16806650622 | 151 | 0.9764 | 0.9343 | 0.5481 | 109963980 | 16492519393 | 149 | 0.9799 | 0.9389 | 0.5483 | 764914 | 460189941 | 300 | 235888 | 596 | 340 |
| A43 | 108470032 | 16378974832 | 151 | 0.9739 | 0.9292 | 0.5104 | 106911116 | 16089280814 | 150 | 0.9781 | 0.9344 | 0.5101 | 589497 | 383296266 | 300 | 413503 | 670 | 347 |
| A47 | 93028636 | 14047324036 | 151 | 0.9707 | 0.9194 | 0.4666 | 92018900 | 13875006281 | 150 | 0.9738 | 0.9233 | 0.4665 | 433085 | 341167210 | 300 | 625500 | 904 | 371 |
| A49 | 93305394 | 14089114494 | 151 | 0.97 | 0.9197 | 0.4674 | 91917706 | 13857846333 | 150 | 0.9743 | 0.9251 | 0.467 | 403363 | 328165130 | 300 | 545085 | 944 | 376 |
| A53 | 102283930 | 15444873430 | 151 | 0.9718 | 0.9239 | 0.4697 | 100805668 | 15196166004 | 150 | 0.9759 | 0.929 | 0.469 | 452532 | 342709404 | 300 | 389460 | 840 | 362 |
| A61 | 84284986 | 12727032886 | 151 | 0.974 | 0.9246 | 0.452 | 83187314 | 12535352032 | 150 | 0.9777 | 0.929 | 0.4514 | 247587 | 190704272 | 300 | 182197 | 877 | 365 |
| A65 | 96965522 | 14641793822 | 151 | 0.977 | 0.9308 | 0.4186 | 95827252 | 14447249345 | 150 | 0.9803 | 0.9347 | 0.4176 | 79563 | 94250909 | 300 | 415505 | 2306 | 420 |
| A66 | 83389520 | 12591817520 | 151 | 0.9739 | 0.9241 | 0.3898 | 82338522 | 12407732417 | 150 | 0.9779 | 0.929 | 0.3888 | 108978 | 106312511 | 300 | 287543 | 1236 | 410 |
| A67 | 96147242 | 14518233542 | 151 | 0.9778 | 0.9317 | 0.4338 | 95207882 | 14365431181 | 150 | 0.9805 | 0.935 | 0.4336 | 215134 | 192447152 | 300 | 274559 | 1130 | 385 |
| A72 | 86254368 | 13024409568 | 151 | 0.9731 | 0.9203 | 0.4124 | 85406620 | 12887040019 | 150 | 0.9758 | 0.9235 | 0.4118 | 66501 | 93205443 | 300 | 416765 | 3387 | 466 |
| A78 | 100771250 | 15216458750 | 151 | 0.9764 | 0.9291 | 0.4375 | 99651262 | 15036405058 | 150 | 0.9794 | 0.9328 | 0.4369 | 222736 | 197743066 | 300 | 292787 | 1106 | 382 |
| A89 | 96976260 | 14643415260 | 151 | 0.9801 | 0.9387 | 0.505 | 96131436 | 14484639201 | 150 | 0.9826 | 0.9417 | 0.5047 | 623282 | 475014377 | 300 | 189960 | 857 | 363 |
| A90 | 124475720 | 18795833720 | 151 | 0.9753 | 0.9273 | 0.5045 | 123264630 | 18566009036 | 150 | 0.9781 | 0.9307 | 0.5043 | 790623 | 574330970 | 300 | 294962 | 809 | 353 |
| A91 | 84647464 | 12781767064 | 151 | 0.9708 | 0.9142 | 0.3883 | 83886376 | 12649008533 | 150 | 0.9733 | 0.9173 | 0.3878 | 42487 | 51868712 | 300 | 259335 | 2651 | 415 |
| A93 | 98031396 | 14802740796 | 151 | 0.9714 | 0.9167 | 0.4096 | 97054674 | 14612278413 | 150 | 0.9743 | 0.9201 | 0.4088 | 94725 | 94057949 | 300 | 275450 | 1526 | 383 |
| A98 | 97092246 | 14660929146 | 151 | 0.9719 | 0.9196 | 0.4855 | 95974096 | 14477093246 | 150 | 0.9752 | 0.9235 | 0.4852 | 514394 | 385809994 | 300 | 223419 | 845 | 358 |
| A101 | 104604238 | 15795239938 | 151 | 0.9719 | 0.9195 | 0.4781 | 103411176 | 15595664395 | 150 | 0.9751 | 0.9233 | 0.4778 | 550882 | 420920161 | 300 | 230811 | 873 | 360 |
| A102 | 90136836 | 13610662236 | 151 | 0.9696 | 0.9135 | 0.4309 | 89166360 | 13451269570 | 150 | 0.9728 | 0.9172 | 0.4306 | 232664 | 195109790 | 300 | 174429 | 1027 | 364 |
| A112 | 96440392 | 14562499192 | 151 | 0.9692 | 0.9136 | 0.4657 | 95184368 | 14357953630 | 150 | 0.9729 | 0.9179 | 0.4652 | 510233 | 389015364 | 300 | 153071 | 873 | 358 |

| **Table S6. Detailed information on collected alfalfa silage** | | | |
| --- | --- | --- | --- |
| **Sample ID** | **Location** | **Silage Time** | **Temperature** |
| A1 | Hebei | 2021-06 | 25℃ |
| A2 | Hebei | 2021-06 | 25℃ |
| A3 | Hebei | 2021-06 | 25℃ |
| A4 | Hebei | 2021-06 | 25℃ |
| A5 | Hebei | 2021-06 | 25℃ |
| A6 | Hebei | 2021-06 | 25℃ |
| A7 | Hebei | 2021-06 | 25℃ |
| A8 | Hebei | 2021-06 | 25℃ |
| A9 | Hebei | 2021-06 | 25℃ |
| A10 | Hebei | 2021-06 | 25℃ |
| A11 | Hebei | 2021-06 | 25℃ |
| A12 | Hebei | 2021-06 | 25℃ |
| A13 | Hebei | 2021-06 | 25℃ |
| A14 | Hebei | 2021-06 | 25℃ |
| A15 | Hebei | 2021-06 | 25℃ |
| A16 | Hebei | 2021-06 | 25℃ |
| A17 | Hebei | 2021-06 | 25℃ |
| A18 | Hebei | 2021-06 | 25℃ |
| A19 | Inner Mongolia | 2021-08 | 22℃ |
| A20 | Inner Mongolia | 2021-08 | 22℃ |
| A21 | Inner Mongolia | 2021-08 | 22℃ |
| A22 | Inner Mongolia | 2021-08 | 22℃ |
| A23 | Inner Mongolia | 2021-08 | 22℃ |
| A24 | Inner Mongolia | 2021-08 | 22℃ |
| A25 | Tianjin | 2021-06 | 26℃ |
| A26 | Tianjin | 2021-06 | 26℃ |
| A27 | Henan | 2022-05 | 22℃ |
| A28 | Henan | 2022-05 | 22℃ |
| A29 | Henan | 2022-05 | 22℃ |
| A30 | Henan | 2022-05 | 22℃ |
| A31 | Henan | 2022-05 | 22℃ |
| A32 | Henan | 2022-05 | 22℃ |
| A33 | Henan | 2022-05 | 22℃ |
| A34 | Henan | 2022-05 | 22℃ |
| A35 | Henan | 2022-05 | 22℃ |
| A36 | Henan | 2022-05 | 22℃ |
| A37 | Shandong | 2022-05 | 21℃ |
| A38 | Shandong | 2022-05 | 21℃ |
| A39 | Shandong | 2022-05 | 21℃ |
| A40 | Shandong | 2022-05 | 21℃ |
| A41 | Hunan | 2022-06 | 25℃ |
| A42 | Hunan | 2022-06 | 25℃ |
| A43 | Hunan | 2022-06 | 25℃ |
| A44 | Hunan | 2022-06 | 25℃ |
| A45 | Hunan | 2022-06 | 25℃ |
| A46 | Hunan | 2022-06 | 25℃ |
| A47 | Hunan | 2022-06 | 25℃ |
| A48 | Hunan | 2022-06 | 25℃ |
| A49 | Hunan | 2022-06 | 25℃ |
| A50 | Hunan | 2022-06 | 25℃ |
| A51 | Hunan | 2022-06 | 25℃ |
| A52 | Hunan | 2022-06 | 25℃ |
| A53 | Hunan | 2022-06 | 25℃ |
| A54 | Hunan | 2022-06 | 25℃ |
| A55 | Hunan | 2022-06 | 25℃ |
| A56 | Hunan | 2022-06 | 25℃ |
| A57 | Hunan | 2022-06 | 25℃ |
| A58 | Hunan | 2022-06 | 25℃ |
| A59 | Inner Mongolia | 2022-08 | 20℃ |
| A60 | Inner Mongolia | 2022-08 | 20℃ |
| A61 | Inner Mongolia | 2022-08 | 20℃ |
| A62 | Inner Mongolia | 2022-08 | 20℃ |
| A63 | Inner Mongolia | 2022-08 | 20℃ |
| A64 | Inner Mongolia | 2022-08 | 20℃ |
| A65 | Shanxi | 2022-06 | 25℃ |
| A66 | Shanxi | 2022-06 | 25℃ |
| A67 | Shanxi | 2022-06 | 25℃ |
| A68 | Shanxi | 2022-06 | 25℃ |
| A69 | Shanxi | 2022-06 | 25℃ |
| A70 | Shanxi | 2022-06 | 25℃ |
| A71 | Shanxi | 2022-06 | 25℃ |
| A72 | Shanxi | 2022-06 | 25℃ |
| A73 | Shanxi | 2022-06 | 25℃ |
| A74 | Shanxi | 2022-06 | 25℃ |
| A75 | Shanxi | 2022-06 | 25℃ |
| A76 | Shanxi | 2022-06 | 25℃ |
| A77 | Shanxi | 2022-06 | 25℃ |
| A78 | Shanxi | 2022-06 | 25℃ |
| A79 | Shanxi | 2022-06 | 25℃ |
| A80 | Shanxi | 2022-06 | 25℃ |
| A81 | Shanxi | 2022-06 | 25℃ |
| A82 | Inner Mongolia | 2022-08 | 22℃ |
| A83 | Inner Mongolia | 2022-08 | 22℃ |
| A84 | Inner Mongolia | 2022-08 | 22℃ |
| A85 | Inner Mongolia | 2022-08 | 22℃ |
| A86 | Inner Mongolia | 2022-08 | 22℃ |
| A87 | Inner Mongolia | 2022-08 | 22℃ |
| A88 | Inner Mongolia | 2022-08 | 22℃ |
| A89 | Inner Mongolia | 2022-08 | 22℃ |
| A90 | Inner Mongolia | 2022-08 | 22℃ |
| A91 | Inner Mongolia | 2022-08 | 22℃ |
| A92 | Inner Mongolia | 2022-08 | 22℃ |
| A93 | Inner Mongolia | 2022-08 | 22℃ |
| A94 | Inner Mongolia | 2022-08 | 22℃ |
| A95 | Inner Mongolia | 2022-08 | 22℃ |
| A96 | Inner Mongolia | 2022-08 | 22℃ |
| A97 | Inner Mongolia | 2022-08 | 22℃ |
| A98 | Hebei | 2022-07 | 29℃ |
| A99 | Hebei | 2022-07 | 29℃ |
| A100 | Hebei | 2022-07 | 29℃ |
| A101 | Hebei | 2022-07 | 29℃ |
| A102 | Hebei | 2022-07 | 29℃ |
| A103 | Hebei | 2022-07 | 29℃ |
| A104 | Hebei | 2022-07 | 29℃ |
| A105 | Hebei | 2022-07 | 29℃ |
| A106 | Hebei | 2022-07 | 29℃ |
| A107 | Hebei | 2022-07 | 29℃ |
| A108 | Hebei | 2022-07 | 29℃ |
| A109 | Hebei | 2022-07 | 29℃ |
| A110 | Hebei | 2022-07 | 29℃ |
| A111 | Hebei | 2022-07 | 29℃ |
| A112 | Hebei | 2022-07 | 29℃ |
| A113 | Hebei | 2022-07 | 29℃ |
| A114 | Hebei | 2022-07 | 29℃ |
| A115 | Hebei | 2022-07 | 29℃ |

| **Tbale S7 Microbial diversity, pH and organic acids of samples from different regions** | | | | | | |
| --- | --- | --- | --- | --- | --- | --- |
| **Location** | **Shannon** | **pH** | **LA**  **(g/kg DM)** | **AA**  **(g/kg DM)** | **PA**  **(g/kg DM)** | **BA**  **(g/kg DM)** |
| Hebei | 2.98 b | 4.68 bc | 47.50 ab | 16.22 b | 4.12 d | 1.31 b |
| Inner Mongolia | 3.25 b | 4.72 bc | 46.54 ab | 27.82 ab | 16.50 b | 1.05 b |
| Tianjin | 2.83 b | 4.73 bc | 54.91 a | 25.76 ab | 7.04 cd | 3.23 a |
| Henan | 3.04 b | 4.70 bc | 36.64 bc | 18.79 b | 12.62 bc | 1.08 b |
| Shandong | 4.31 a | 5.14 a | 29.87c | 22.75 ab | 9.97 bcd | 2.36 ab |
| Hunan | 2.99 b | 4.93 ab | 48.07 ab | 36.50 a | 12.91 bc | 2.49 ab |
| Shanxi | 2.92 b | 4.54 c | 53.32a | 34.09 a | 27.78 a | 1.64 ab |
| LA, lactic acid; AA, acetic acid; PA, propionic acid; BA, butyric acid | | | | | | |

Different lowercase letters in the same line (a-d) indicate a significant difference (*P* < 0.05).
